# Severely Bent Dinitrogen Bridging in Highly Preorganized Dinuclear Cobalt Complexes Featuring an Intricate Electronic Structure

**DOI:** 10.1021/jacsau.5c00129

**Published:** 2025-06-20

**Authors:** Yue Wang, Shweta Singh, Andreas Meyer, Sebastian Dechert, Sandeep K. Gupta, Serhiy Demeshko, Vera Krewald, Franc Meyer

**Affiliations:** † Institute of Inorganic Chemistry, 9375 University of Göttingen, Tammannstrasse 4, D-37077 Göttingen, Germany; ‡ Fachbereich Chemie, Quantenchemie, 26536 Technische Universität Darmstadt, Peter-Grünberg-Str. 4, D-64287 Darmstadt, Germany; § 28282 Max Planck Institute for Multidisciplinary Sciences, Am Fassberg 11, D-37077 Göttingen, Germany; ∥ Institute of Physical Chemistry, University of Göttingen, Tammannstrasse 6, D-37077 Göttingen, Germany; ⊥ Department of Chemistry, Indian Institute of Technology Delhi, New Delhi 110016, India; # International Center for Advanced Studies of Energy Conversion (ICASEC), University of Göttingen, Tammannstrasse 6, D-37077 Göttingen , Germany

**Keywords:** dinitrogen complexes, cobalt complexes, structural constraints, electronic structure, CASSCF calculations, dinitrogen silylation

## Abstract

Preorganized bimetallic complexes could open up new avenues of cooperative substrate activation and transformation of inert dinitrogen (N_2_). While the most common structural motif in synthetic dinuclear N_2_ complexes is a linear M–N–N–M unit, only a few examples of bent geometries reminiscent of the proposed N_2_ binding modes in the Haber–Bosch process or the M-cluster of Nitrogenase have been reported. Exploiting the structural constraints imposed by a compartmental pyrazolato/β-diketiminato hybrid ligand platform (L^3̵–^), we here report a series of dicobalt­(I) complexes [LCo_2_(N_2_)]^−^ hosting N_2_ within the preorganized bimetallic cleft with extremely acute Co-Ct_N2_-Co angles of around 123.5° (Ct_N2_ is the N_2_ centroid). A detailed electronic structure analysis using wave function methods shows that the ground state of diamagnetic [LCo_2_(N_2_)]^−^ may not be a simple closed-shell singlet, but rather of multiconfigurational nature subject to spin–orbit coupling. Co–N–N–Co bending significantly decreases overlap of metal d-orbitals with the in-plane p­(N) orbitals, yet the N_2_ substrate is substantially more activated (*ν̃*_NN_ ≈ 1900 cm^–1^) than in most Co^I^ complexes with end-on bound N_2_ or linear Co–N–N–Co arrangement; no coactivation by the alkali cation K^+^ is observed for [LCo_2_(N_2_)]^−^. Reversible oxidation gives an unusual mixed-valent complex [LCo^I^Co^II^(N_2_)] in which the highly bent Co–N–N–Co core is retained. [LCo_2_(N_2_)]­[K­(THF)_2_] is found to cocrystallize with KBEt_3_H, indicating that the putative [LCo_2_(N_2_H)]^2–^ has a very low hydricity. In presence of KC_8_ and Me_3_SiCl, complex [LCo_2_(N_2_)]^−^ serves as (pre)­catalyst for the reductive silylation of N_2_ into N­(SiMe_3_)_3_. We discuss the implications of the highly exposed, “*cis*-bent” N_2_ unit for onward reactivity.

## Graphical abstract

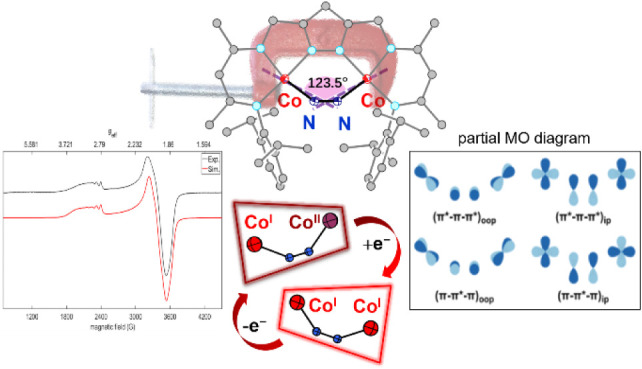

## Introduction

Multinuclear transition metal sites are important in man-made and natural dinitrogen fixation. Dinitrogen can bind in end-on fashion at a single metal ion or as a bridging unit between two or more metal ions.^1^ In the Haber–Bosch process, the binding and activation of N_2_ at the catalyst surface is thought to involve a bent μ_1,2_-bridging intermediate between two metal sites prior to N–N bond cleavage (α-N_2_; Figure 1 top).^2^ For the M-cluster of Nitrogenase, end-on and bridging N_2_ binding modes have been suggested, including substrate activation in a bent bridging fashion.^3^ On the other hand, synthetic model complexes with two or more transition metal ions that are capable of activating or splitting dinitrogen are predominantly characterized by linear M–N–N–M arrangements,^4,5^ whose bonding motifs have recently been systematically classified.^6^ Unusual bent geometries have been reported for a few dinuclear complexes of early to mid transition metals, e.g., Zr (M–N–N angle θ = 144.1°, angle involving the dinitrogen centroid M–Ct_N2_–M φ = 127.0°),^7^ V (θ = 162.4°, φ = 155.9°)^8^ and Cr (θ = 162.0°, φ = 155.1°).^9^ Only more recently, iron complexes with bent Fe–N–N–Fe units have become available (Figure 1). Mindiola and coworkers found this motif in bis­(pyrrolyl)­pyridine-supported dinuclear Fe^II^ complexes **I** with φ = 154.0° and φ = 157.2° depending on the solvent used during crystallization, and φ = 148.5° upon 2-fold reduction of the ligands.^10^ In Tomson’s ^3^PDI_2_-supported complexes **II**, Fe–Ct_N2_–Fe angles of φ = 150.1° and φ = 151.7° depending on the ligand substituent were observed.^11^ Compartmental ligands with two arene-linked β-diketiminato (BDI) binding sites are the basis of the *S* = 7/2 iron dimer **III** reported by Murray and coworkers^12^ as well as the unsymmetric type **IV** diiron dinitrogen complexes recently reported by Xi and coworkers;^13^ the former features φ = 158.5° or 159.1° depending on the cation,^12^ and the latter could be isolated in two oxidation states with Fe–Ct_N2_–Fe angles in the range 154° – 158°.^13^ These examples highlight that a bent M–N–N–M motif can be achieved in molecular complexes of suitable dinucleating ligand scaffolds, yet the degree of bending is also influenced by steric constraints of the ligands as well as more subtle environmental effects. Overall, the Fe–Ct_N2_–Fe angles in the mentioned diiron complexes with constrained geometry are found in the range 150–158° (Figure 1), much less acute than the 130° estimated for the Fe–Ct_N2_–Fe angle of the α-N_2_ intermediate that is key to the N–N bond activation in the Haber-Bosch process.^2,11^

**Figure 1:**
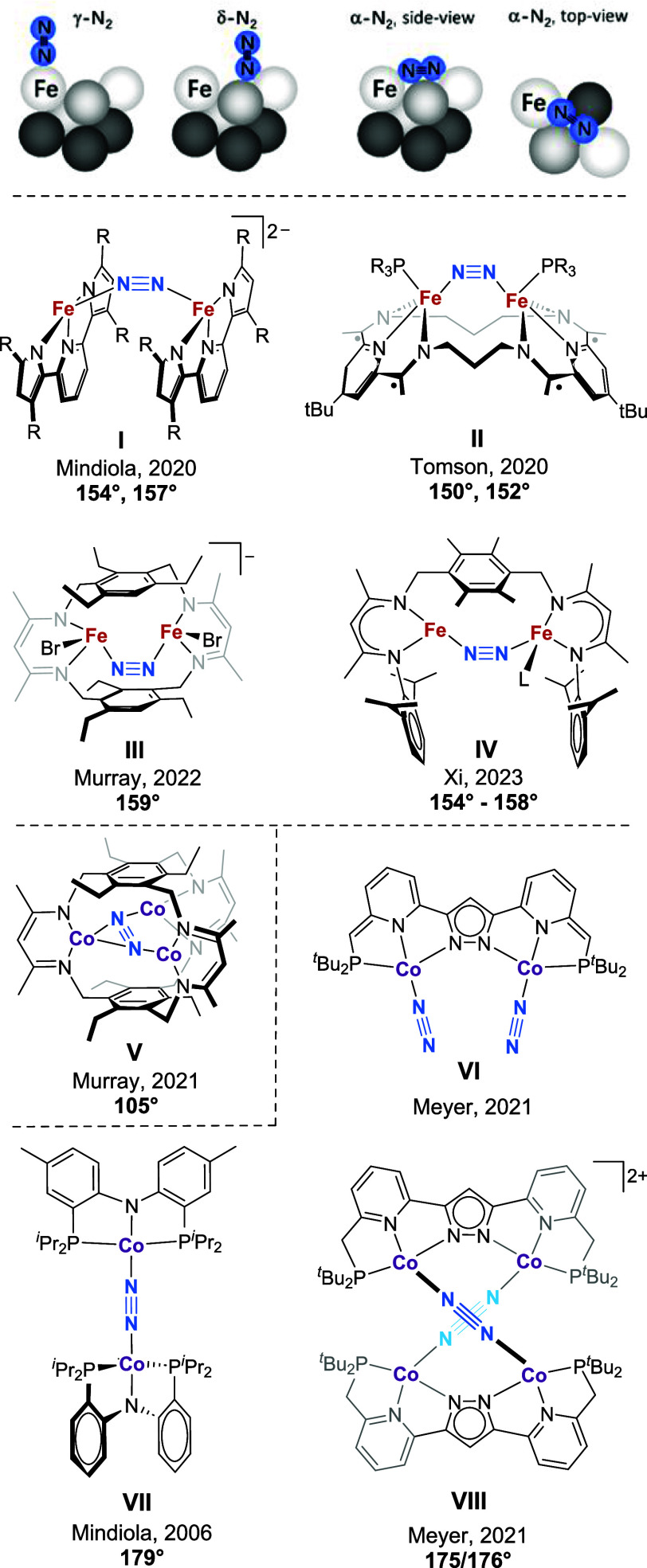
Proposed binding motifs of N_2_ on the Fe(111) surface of the Haber–Bosch catalyst (top), dinuclear Fe complexes **I** – **IV** with bent μ_1,2_-η^1^:η^1^-N_2_ (*R* = *t*Bu in **I**, *R* = Ph, Me in **II**, L = Et_2_O, THF, DMAP in **IV**), tricobalt complex **V** with μ_3_-η^1^:η^2^:η^1^-N_2_, and previously reported pyrazolato-based dicobalt­(I) dinitrogen complexes **VI** and **VIII** and linear dicobalt­(I) complex **VII**; for **I** – **V**, **VII** and **VIII** angles M–Ct_N2_–M are given (see text).

A more complex situation with severely bent M–N–N–M fragments can be found in few trinuclear complexes that feature a μ_3_-η^1^:η^2^:η^1^ coordination mode of bound N_2_, as reported for Ti^14^ and for Murray’s Cu_3_ ^15^ and Co_3_ ^16^ complexes of a tris­(β-diketiminato) cyclophane encapsulating N_2_ within their internal cavity (**V**, Figure 1). Considering just the Co–(μ_1,2_-η^1^:η^1^-N_2_)–Co fragment, **V** shows a very acute Co–Ct_N2_–Co angle of 105° at a short Co···Co distance of 3.66 Å; the N_2_ was found disordered in the crystal and NMR data indicated a low barrier for in-plane N_2_ rotation in the cavity. Spectroscopic and computational analysis revealed that the third Co^I^ significantly increases the extent of N_2_ activation via covalent Co−η^2^(N_2_) interaction. The ground state of **V** has been reported as an apparent triplet due to moderate antiferromagnetic coupling within the Co–(μ_1,2_-η^1^:η^1^-N_2_)–Co subunit.^16^

Alkali metals are found in the vicinity of the bridging N_2_ ligands in several of the examples with bent M–N–N–M motifs or variants thereof. For **I**, computational studies indicated that K^+^ ions are essential to maintain the bent N_2_ coordination.^10^ Holland and coworkers invoked alkali metals for N_2_ splitting,^17^ mirroring the utility of potassium as a promotor in the Haber–Bosch catalyst.^18^ The role of alkali metals may be a simple electrostatic stabilization of the formed complex in the given environment or an active electronic enhancement of the N_2_ bond weakening.

BDI ligands are particularly prominent in molecular chemistry aiming at N_2_ activation (including **III** – **V**),^19^ and we recently introduced a dinucleating ligand scaffold [L]^3–^ featuring a central pyrazolate bridge and two BDI chelate arms.^20^ This ligand forms highly preorganized dinickel­(II) complexes capable of binding various small molecules in a constrained nonlinear geometry within the bimetallic cleft.^21,22^ Here we present the first dicobalt complexes of [L]^3–^, specifically the synthesis, isolation and comprehensive characterization of a dicobalt­(I)-dinitrogen complex and its 1e^–^ oxidized mixed-valent congener featuring the most acute M–Ct_N2_–M angles documented in the literature so far. The degree of bending appears to be independent of coactivation by alkali metal ions, and a detailed computational analysis revealed an intricate electronic structure with pronounced multiconfigurational character.

## Results and Discussion

### Extremely Bent Dicobalt­(I) Dinitrogen Complexes

Deprotonation of the proligand H_3_L with KHMDS (3.0 equiv) and subsequent reaction with CoBr_2_ formed LCo_2_Br (**1**; Scheme 1) in up to 46% crystalline yield. In **1** the cobalt ions reside in a square planar environment with τ_4_ values in the ranges 0.11–0.12 for both Co1 and Co2 (*viz*. τ_4_ = 1 for perfect tetrahedral geometry and τ_4_ = 0 for perfect square planar geometry).^23^ The Co···Co distance of 3.89 Å is slightly elongated compared to the Ni···Ni distance of 3.81 Å in LNi_2_Br;^20^ details of the molecular structure of **1** are provided in the SI. The ^1^H NMR spectrum measured in THF-*d*_8_ indicated a paramagnetic nature of **1**, and variable-temperature magnetic susceptibility data recorded on a powdered crystalline sample corroborated the presence of two low-spin Co^II^ ions (*S* = 1/2, *g* = 2.65)^24^ that are antiferromagnetically coupled (*J* = −39.2 cm^–1^ for Ĥ=−2JS1^S2^+gμBB⃗(S1⃗+S2⃗) ) to give a diamagnetic (*S*_T_ = 0) ground state (Figure S32).

**Scheme 1:**
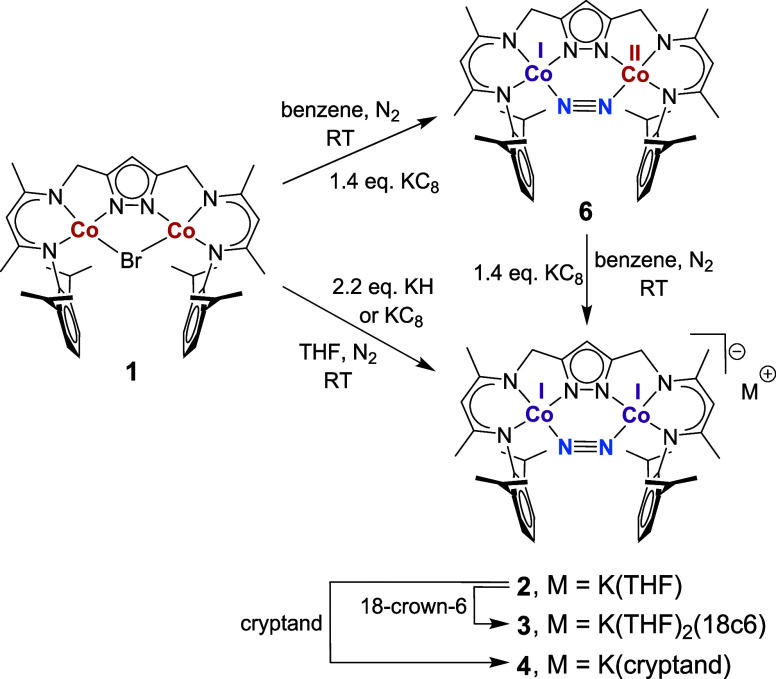
Synthesis of Dicobalt–Dinitrogen Complexes 2–4 and 6

Chemical reduction of **1** with KH or KC_8_ (2.2 equiv) under N_2_ atmosphere resulted in a color change from red to purple, and the dicobalt dinitrogen complex [LCo_2_(N_2_)]­[K­(THF)] (**2**) was isolated in the form of dark crystals in 55% yield (Scheme 1). When 18-crown-6 (1.2 equiv) was added to the reaction mixture, a navy-blue colored solution was produced from which dark crystals of [LCo_2_(N_2_)]­[K­(THF)_2_(18c6)] (**3**) were collected in good yield (64%). Similarly, the addition of [2,2,2]­cryptand gave [LCo_2_(N_2_)]­[K­(cryptand)] (**4**), albeit the crystalline material was contaminated with ∼ 5% of the starting complex **1** (see SI for details).

Complexes **2** – **4** were characterized by single crystal X-ray diffraction (SC-XRD), and in all cases the bound N_2_ is found in extremely bent μ_1,2_-η^1^:η^1^ bridging mode within the bimetallic pocket (Figure 2), spanning the two cobalt ions with Co···Co distances of around 4.02 Å which is similar to the metal···metal distance of around 4.1 Å for top-layer iron atoms of the Fe(111) surface relevant to the Haber-Bosch process.^2,11^ In case of **4**, the complex is cocrystallized with **1** in a ratio of 0.95­(**4**):0.05­(**1**). All cobalt ions adopt a roughly square planar coordination geometry (τ_4_ values in the range 0.12 – 0.14). The three complexes differ in the location of the cation: while **3** and **4** feature separated ion pairs, in **2** the K^+^ (with one additional THF) is situated above two neighboring pyrazolate rings (Figure 3) with distances between K^+^ and the centroids of the pyrazolates Ct_py_–K^+^ in the range 2.889 – 3.053 Å, which is in the typical range known for cation−π interactions of K^+^ with aromatic systems.^25^ Similar η^5^ coordination of K^+^ to a pyrazolate group has previously been reported in a few anionic pyrazolato-bridged bimetallic complexes.^26^ It should be noted that in many dinickel­(II) complexes of [L]^3–^, such as the peroxido complex [KLNi_2_(O_2_)],^21^ the K^+^ is held via cation-π bonding between the two aryl flaps of the ligand scaffold, exhibiting favorable side-on interaction with the dianionic substrate within the bimetallic pocket. The preference of the K^+^ for π-interaction with the pyrazolate may suggest a rather small Co→N_2_ charge transfer and minor negative charge accumulation on the bound N_2_ in **2**.

**Figure 2:**
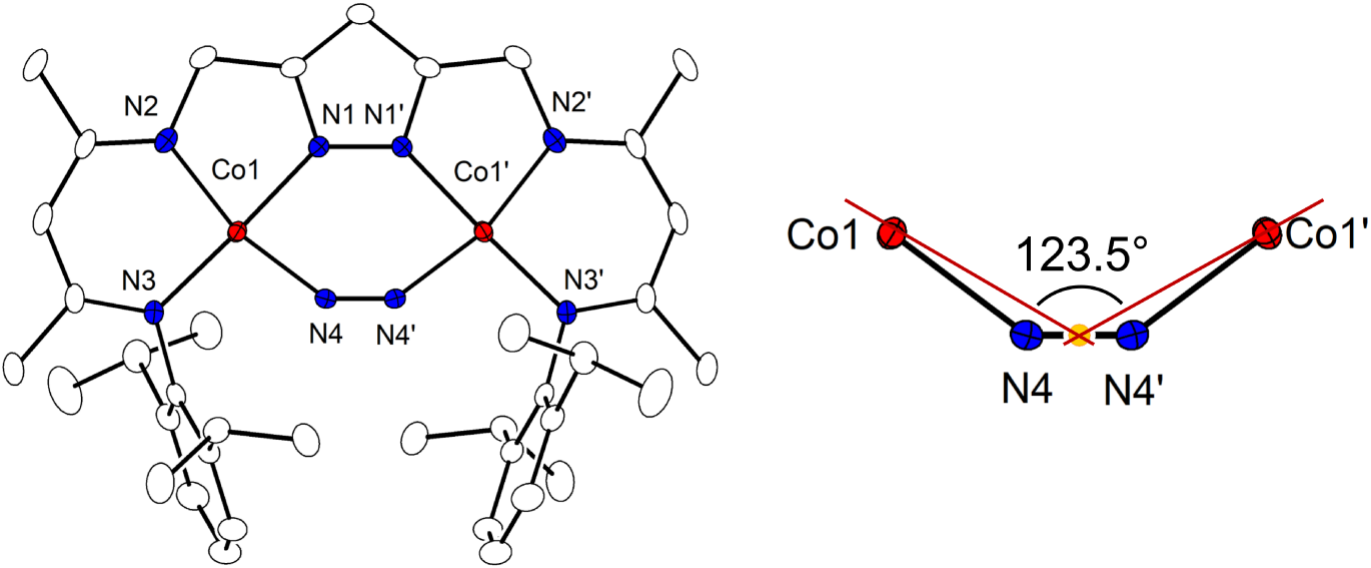
Left: Molecular structure of the anion of **3** (30% probability thermal ellipsoids); all hydrogen atoms and the counterion are omitted for clarity. Right: Co-Ct_N2_-Co angle defining the bent μ_1,2_-η^1^:η^1^-N_2_ binding mode. Symmetry transformation used to generate equivalent atoms: (’) 1/2–*x*, 1/2–*y*, *z*.

**Figure 3:**
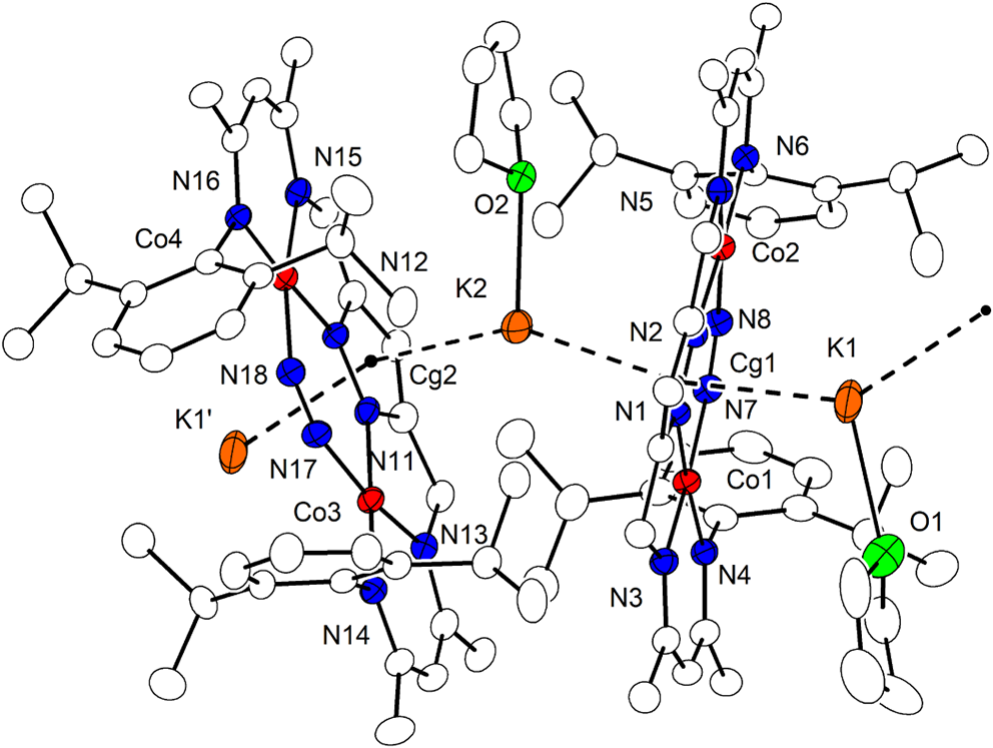
Molecular structure of **2** (30% probability thermal ellipsoids) emphasizing the 1D chain arrangement resulting from π-interaction of the K^+^ with the pyrazolate groups; all hydrogen atoms are omitted for clarity. Symmetry transformation used to generate equivalent atoms: (’) 1+x, y, z. Cg is defined as the centroid of the five pyrazolate ring atoms.

Inspection of the N_2_ ligand in **2** – **4** suggests that it is moderately activated as indicated by the N–N distances of 1.144(10) and 1.135(9) Å in **2** (two independent [LCo_2_(N_2_)]^−^ in the asymmetric unit), 1.138(5) Å in **3**, and 1.148 (2) Å in **4** (Table 1), which is slightly longer than in free N_2_ (1.098 Å).^27^ Most notable is the extremely acute Co-Ct_N2_-Co angle that is found in the narrow range 123.3° – 123.6° for **2** – **4**, much smaller than in the recently reported diiron dinitrogen complexes **I** – **IV** with a bent Fe–(μ_1,2_-η^1^:η^1^-N_2_)–Fe arrangement (Figure 1, Table 1). The disordered N_2_ in the cavity of the tris­(β-diketiminato) cyclophane Co_3_ complex **V** represents a special case since it features an additional η^2^-bound Co ion that reduces the substrate to the diazene level.^16^ The close similarity of the core structures of **2** – **4** suggests a negligible influence of the K^+^ cation and whether it is η^5^-bound to the pyrazolate or not.

**Table 1:** Selected Metrical Parameters and IR Frequencies for Constrained M–N_2_–M Cores

	*d*(N–N) [Å]	∠ M-Ct_N2_-M [°]	ν (N–N) [cm^–1^]; solid
K_2_ **I** ^10^	1.189(2)^a^	154.0^a^	1696
	1.187(9)^b^	157.2^b^	
K[(18-crown-6)K]**I** ^10^	1.223(2)	148.5	1654
**II** (*R* = Ph)^11^	1.139(3)	151.7	1959
**II** (*R* = Me)^11^	1.135(3)	150.1	2003
[K(18-c-6)]**III** ^12^	1.155(10)	159.1	1934
[K(cryp)]**III** ^12^	1.164(6)	158.5	1932
**IV** ^13^	1.170(4) −1.189(3)	154.0−158.0	1773–1780
**2** ^c^	1.135(9)/ 1.144(10)	123.3/123.4	1901
**3**	1.138(5)	123.5	1905
**4**	1.148(2)	123.6	1899
**6**	1.015(4)^d^	119.7	1953

a crystallized from toluene.

b crystallized from pentane.

c two crystallographically independent LCo_2_(N_2_) moieties in the asymmetric unit.

d the very short N–N bond is likely an artifact due to trace amounts of cocrystallized LCo_2_Br (**1**).

The location of the K^+^ has some moderate effect on the electronic absorptions as evidenced by UV–vis spectra of solid **2** and **3** that show maxima at 565 and 591 nm, respectively (Figure S27; tentatively assigned to metal-to-ligand charge transfer transitions). For **3** in THF solution the λ_max_ is essentially identical (594 nm) while a significant change is observed upon dissolving **2** (λ_max_ = 584 nm), suggesting that in THF solution the K^+^ may dissociate from, or change its location at the anion [LCo_2_(N_2_)]^−^ (Figure S26).

IR spectra of the solid compounds show N–N stretches at 1901 cm^–1^ (**2**), 1905 cm^–1^ (**3**) and 1899 cm^–1^ (**4**), slightly lower than N–N stretching frequencies of diiron complexes **II** and **III** (Table 1) and in line with moderate activation of the N_2_ substrate (c*f*. *ν̃*_NN_ = 2359 cm^–1^ for free N_2_).^27^ Band assignment for **3** was confirmed by ^15^N_2_ isotope labeling showing a shift to 1845 cm^–1^ (Δ­(^15^N_2_^14^N_2_) = –61 cm^–1^, *ν̃*(^14^N^14^N)/ *ν̃*(^15^N^15^N) = 1.033, calculated 1.035 for an isolated harmonic N–N oscillator). It should be noted that mononuclear Co^I^ complexes with end-on bound N_2_ mostly show much higher *ν̃*_NN_ in the range 1970 – 2100 cm^–1^,^28,29^ and the same is true for dinuclear Co^I^–NN–Co^I^ systems.^29–32^ *ν̃*_NN_ = 2000 cm^–1^ was found for complex **VIII** (Figure 1)^31^ that has pyrazolato-bridged Co^I^ ions, and also the dicobalt complex **VII** ^29^ and related systems^32^ composed of two square planar Co^I^ subunits with anionic pincer ligands and a common linear Co–NN–Co arrangement (Figure 1) showed a much less activated N_2_ substrate (*ν̃*_NN_ = 2024 cm^–1^ for **VII**). This suggests a comparatively pronounced reductive N_2_ activation in **2** – **4**. Unfortunately, no value for the N–N stretch had been obtained for Holland’s linear Co^I^–NN–Co^I^ complex with BDI capping ligands and T-shaped three-coordinate metal ions.^33^ In case of Murray’s trinuclear {Co_3_N_2_} complex **V** the N–N stretching vibrations are much lower at 1717/1752 cm^–1^ reflecting the diazene character due to the additional side-on bound cobalt ion.^16^

The similarity of the N–N stretches for solid **2** – **4** confirms that the location of the K^+^ cation, either above the pyrazole ring or separated from the anion, does hardly influence the bonding situation of the N_2_ unit. While the K^+^ ion does not significantly influence the degree of N_2_ activation in the present case, for Fe, Co and Ni dinitrogen complexes with parent BDI ligands, reduction of the N_2_ substrate is often accompanied by close interaction with alkali metal ions.^33,34^ For complex **I**, interaction with K^+^ was even found to be essential to achieve a bent M–(μ_1,2_-N_2_)–M unit.^10^

Solution IR spectra of **2**, **3** and **4** recorded in THF under N_2_ atmosphere display an intense N–N stretching band at around 1909 cm^–1^, which is close to the values recorded for solid material (Figure S19). However, additional absorptions at higher energies (2040 cm^–1^ for **2**, 2050 cm^–1^ for **4**) are observed. These newly formed IR bands are similar to those of the related dicobalt­(I) complex **VI** featuring two tridentate PNN pincer compartments and a pyrazolato bridge: in that case, IR bands at 2009 cm^–1^ and 2032 cm^–1^ were assigned to the antisymmetric and symmetric N–N stretches of the two end-on bound N_2_ ligands.^31^ DFT calculations (BP86/ZORA-def2-TZVP, see SI for details) support that the additional absorptions for **2** – **4** in solution result from the conversion of [LCo_2_(N_2_)]^−^ into a new species [LCo_2_(N_2_)_2_]^−^ (**5**) in which each metal ion coordinates one N_2_ molecule end-on. The calculated antisymmetric and symmetric stretching modes for such a dicobalt­(I) complex **5** are found at 2060 and 2064 cm^–1^ (cf. 2032 and 2046 cm^–1^ for **VI**, both **5** and **VI** are calculated at the same level of theory). We note that binding the second N_2_ molecule to [LCo_2_(N_2_)]^−^ is enthalpically favored (with Δ*H*_calc_(**2**+N_2_⇌**5**) = – 4.6 kcal/mol) but entropically burdened (with Δ*G*_calc_(**2**+N_2_⇌**5**) = +5.07 kcal/mol) at room temperature.

### Redox Chemistry and Mixed-Valent Dicobalt­(I/II) Dinitrogen Complex

Cyclic voltammetry (CV) of **3** in THF under N_2_ atmosphere revealed two redox events, an almost reversible oxidation at −2.00 V and a quasi-reversible reduction at −2.46 V (*E*_1/2_, vs Fc^+/0^ at 100 mVs^–1^, Figure 4a); similar potentials are found for **2** in THF solution (−1.97 V and −2.43 V vs Fc^+/0^ at 100 mVs^–1^; Figure S36), confirming that the encapsulation of K^+^ by 18-crown-6 in **3** has a negligible effect. While the very low potentials made any handling of these sensitive compounds challenging and the quasi-reversible nature of the cathodic process prevented identification of the reduced species, the reversibility of the anodic process prompted experiments toward isolating the oxidized formal Co^I^Co^II^ congener (**6**). Indeed, monitoring the oxidation of **3** by IR spectro-electrochemistry (IR-SEC; Figure 4b) showed the decrease of the N–N stretching band for **3** at 1909 cm^–1^ and the concomitant appearance of a new band at 2002 cm^–1^ for an oxidized dicobalt dinitrogen complex in THF. This interconversion is mostly reversible as subsequent rereduction reverts the spectral changes (Figure S24), although with the appearance of an additional band at 2048 cm^–1^, possibly indicating formation of species [LCo_2_(N_2_)_2_]^−^ (**5**).

**Figure 4:**
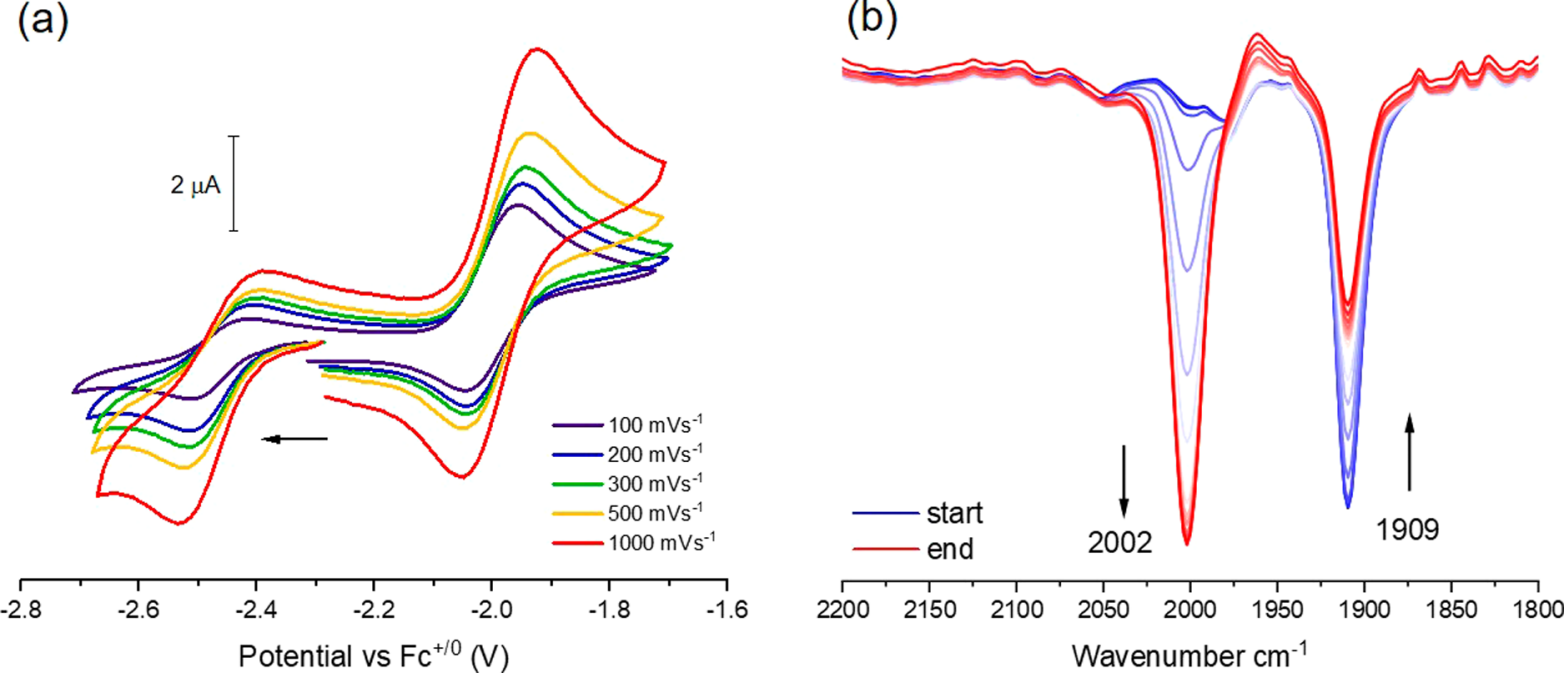
(a) Cyclic voltammogram of **3** (1 mM in THF), using a glassy carbon (3 mm outer diameter) working electrode (WE), a Pt wire counter electrode (CE), a Pt wire reference electrode (RE) and 0.2 M (*^n^* Bu_4_N)­PF_6_ as the supporting electrolyte; (b) SEC-IR spectral monitoring of the oxidation of **3** (3.0 mM of **3** in 0.2 M (*^n^* Bu_4_N)­PF_6_ solution in THF; WE: Pt mesh, CE: Pt wire and RE: Ag wire; scan rate 10 mV/s).

Chemically, the formal Co^I^Co^II^ complex **6** is best prepared directly from the dicobalt­(II) precursor **1** by careful reduction with just slightly more than 1 equiv of KC_8_ in benzene (Scheme 1). When chemical reductions of **1** with KC_8_ are conducted in THF or toluene, a rapid color change is observed and **2** is obtained as a major product. Performing the same reduction in benzene, however, the reaction is much slower, which allows for the isolation of **6** in fair yield (61%) when using 1.4 eq. of KC_8_. Further addition of 1.4 eq. of KC_8_ to the solution of **6** then leads to the dicobalt­(I) product **2** (Scheme 1). The molecular structure of **6** in solid state was elucidated by SC-XRD and revealed that the overall pyrazolate-based core structure with the two cobalt ions in roughly square-planar {N_4_} coordination environment is essentially identical to those of the anions of **2** – **4** (Figure 5; **6** features a crystallographic 2-fold rotation axis). Inspection of the atom distances reveals that the Co–N bonds involving the ligand scaffold [L]^3–^ are slightly shorter in **6** (Table 2), as might be expected from a higher average metal oxidation state, and also the Co···Co separation is slightly shortened. In contrast, the Co–N4 bonds involving the N_2_ substrate in **6** are clearly longer than in **3**, suggesting decreased Co­(d)→N_2_(π*) backbonding and likely also weak N_2_ binding. This results in an extremely small Co-Ct_N2_-Co angle of 119.7°. The N_2_ itself appears to exhibits an N–N bond length of 1.015(4) Å, even shorter than free N_2_, but this is likely an artifact from trace amounts of cocrystallized LCo_2_Br (**1**) (the contribution of **1** is small so that it could not be refined as disorder as in case of **4**; note that also the Co-Ct_N2_-Co angle may be influenced by trace amounts of cocrystallized **1**); as a result, the N4–N4’ bond from the XRD data is not reliable. While a significant number of Co^I^/N_2_ complexes have been reported,^28,29,31^ Co^II^ complexes (or mixed-valent Co^I^Co^II^ complexes) with bound N_2_ are rare.^16,35^

**Figure 5:**
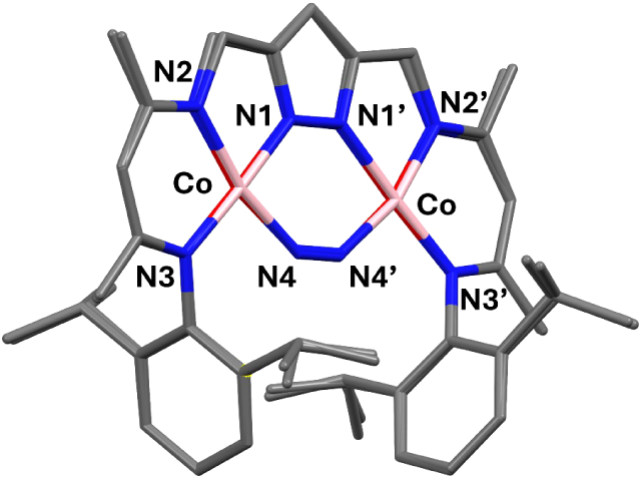
Structural overlay of **6** and the anion of **3** in ball–stick mode.

**Table 2:** Selected Metric Parameters for the Dicobalt Cores of 3 and 6 (Both with Twofold Symmetry)

	**3**	**6**
*d*(Co···Co) [Å]	4.0240(6)	3.9766(5)
*d*(Co–N1) [Å]	1.910(3)	1.8834(18)
*d*(Co–N2) [Å]	1.883(3)	1.8755(18)
*d*(Co–N3) [Å]	1.883(3)	1.8730(19)
*d*(Co–N4) [Å]	1.803(3)	1.878(2)
∠ Co-Ct_N2_-Co [°]	123.5	119.7

The ATR-IR spectrum of solid **6** shows a prominent band at 1953 cm^–1^ for the N–N stretch; assignment is confirmed by ^15^N_2_ isotope labeling showing a shift to 1890 cm^–1^ (Δ­(^15^N_2_^14^N_2_) = –63 cm^–1^, *ν̃* (^14^N^14^N)/*ν̃* (^15^N^15^N) = 1.033). The shift to higher energy by around 50 cm^–1^ compared to the N–N stretches of **2** – **4** (Figures S16–S18, Table 1) is in accordance with the structural data and decreased Co­(d)→N_2_(π*) backbonding after oxidation. Still, the degree of N_2_-activation in **6** appears to be surprisingly strong if compared to *ν̃*_NN_ = 2145 cm^–1^ for a square-planar low-spin Co^II^ complex with end-on bound N_2_.^35^ A solution of **6** in toluene shows essentially the same IR absorption at 1954 cm^–1^ (Figure S22), indicating that the molecular structure with its symmetric Co–(μ_1,2_-η^1^:η^1^-N_2_)–Co core remains intact. However, this N–N stretch is 50 cm^–1^ lower in energy than the 2003 cm^–1^ observed upon oxidation of **3** in THF during the IR-SEC experiment. Indeed, dissolving crystalline **6** in THF under N_2_ atmosphere gives an IR band at 2002 cm^–1^, suggesting a different N_2_ binding mode in THF solution. DFT calculations predict a value of 1983 cm^–1^ for **6**, while binding of a second dinitrogen molecule to give hypothetical [LCo_2_(N_2_)_2_] (**6·N**_**2**_) results in symmetric and antisymmetric stretches at 2078 cm^–1^ and 2122 cm^–1^, respectively (see Table S11). In a structure [LCo_2_(N_2_)­(THF)] where a THF molecule is bound to one cobalt site, rendering the N_2_ bound in end-on fashion to the other cobalt ion, a stretching frequency of 2026 cm^–1^ is obtained (**6·THF** in Table S13). This provides a reasonable explanation for the experimental observation. Ultimately, complex **6** is not stable in THF, evidenced by the gradual disappearance of the IR band at 2002 cm^–1^ (Figure S23); it reacts within around 1 h to give a new but unknown paramagnetic species, likely reflecting substitution of the weakly bound end-on N_2_.

### NMR, EPR, and Magnetic Properties

NMR spectroscopy (for **2**, **3**; Figures S2–S9) and SQUID magnetometry (for **3**; Figure 6) show that dicobalt­(I) complexes **2** and **3** (and likely also **4**) are diamagnetic in solution and in solid state. ^1^H and ^13^C NMR spectra indicate C_2v_ symmetry of the [LCo_2_(N_2_)]^−^ anion in THF-*d*_8_. While ^1^H NMR signals are sharp in the case of the separated ion pair **3**, they are broader and temperature dependent in case of **2** (Figures S2, S3), providing further evidence that the location of K^+^ with respect to the [LCo_2_(N_2_)]^−^ anion may be dynamic for **2** in THF.

**Figure 6:**
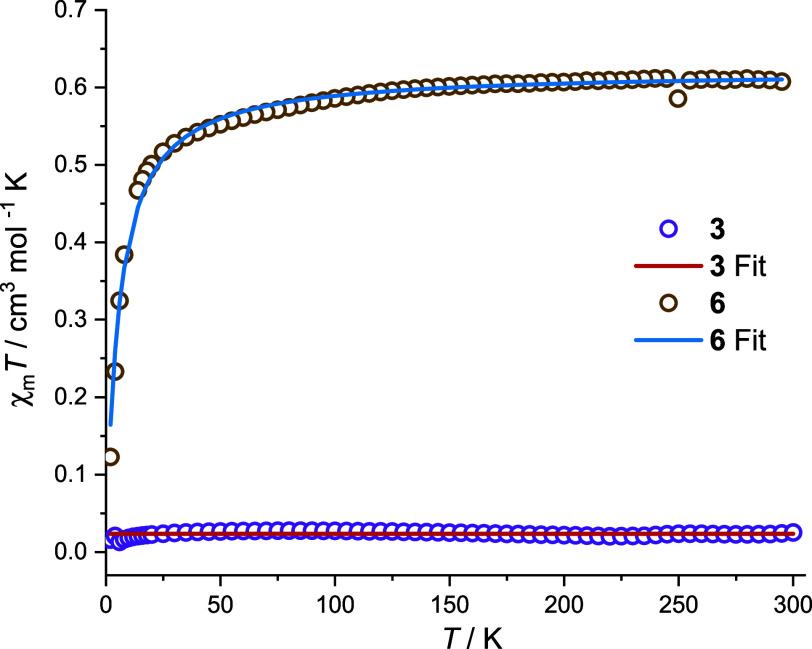
Variable-temperature χ_m_ *T* plots for **3** and **6**. The solid lines are the best fits; in case of **3**, the data were simulated assuming *S* = 0 with 1.3% paramagnetic impurities of S = 3/2.

The ^1^H NMR spectrum of neutral **6** in C_6_D_6_ confirms its paramagnetic nature and shows signals in the broad range from +90 to −65 ppm; their number is in accordance with *C*_2v_ symmetry in solution. SQUID magnetometry of solid **6** shows an almost constant χ_m_ *T* value of ∼ 0.6 cm^3^ mol^–1^ K in the temperature range from rt to around 100 K (Figure 6). At even lower temperatures the χ_m_ *T* value drops to reach 0.12 cm^3^ mol^–1^ K at 2 K. The data can be well simulated assuming an *S*_T_ = 1/2 ground state with *g* = 2.58 and intermolecular interactions with a Weiss temperature Θ = – 5.6 K (see Supporting Information for more details).

The *S*_T_ = 1/2 ground state of **6** in frozen toluene solution at 12 K has been further analyzed using continuous wave X-band electron paramagnetic resonance (CW-EPR) spectroscopy (Figure 7). The spectrum appears slightly rhombic with effective *g* values of ∼3.1 and ∼2.0–1.9. The low field absorption (*g*_eff_ = ∼3.1) reveals a partly resolved hyperfine interaction, whereas no hyperfine interaction is resolved on the high field side. A similar spectrum was previously observed for a square planar, mononuclear, low-spin (*S* = 1/2) Co^II^ complex.^36^ The high similarity of the two spectra indicates that **6** is best described by a localized Co^I^Co^II^ situation on the EPR time scale. To simulate the EPR spectrum, we followed the analysis by McGarvey^37^ and Hitchman^38^ and found *g* = [3.13, 1.90, 1.98] and a hyperfine tensor of *A*(Co) = [320, −85, 100] MHz. We note that the ordering of the tensor elements as well the negative sign of the second *A* tensor element only derives from Hitchman’s analysis but cannot be deduced from our data. To reproduce the asymmetric shape observed on the low field feature we had to assume a correlated distribution of *g* and *A* values, which mostly affects *g*_1_ and *A*_1_ and agrees with Hitchman’s analysis. We found a normal distribution of *g*_1_ and *A*_1_ values centered on the values mentioned above and with standard deviations of Δ*g*_1_ = 0.067 and Δ*A*_1_ = 60 MHz. Finally, we note the presence of a weak signal at *g*_eff_ = ∼ 6.0. This feature is attributed to a minor impurity (see SI for more details).

**Figure 7:**
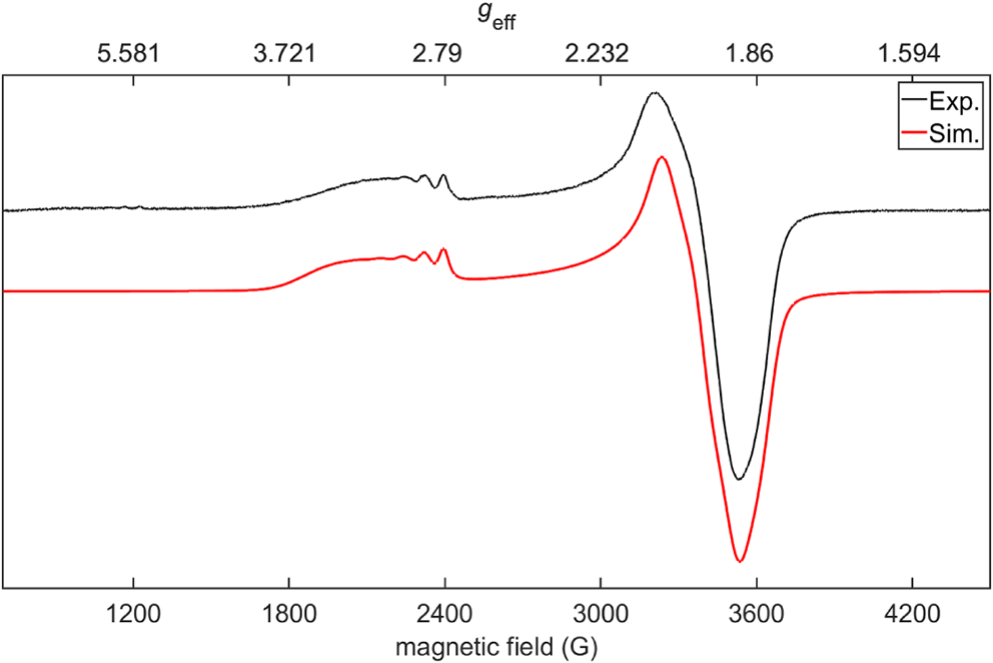
CW-EPR spectrum of **6** in frozen toluene solution, recorded at 12 K. Instrumental parameters for EPR measurement: ν = 9.375 GHz, modulation amplitude = 4 G, MW power = 10.02 mW. Simulation parameters: *S* = 1/2, *g*_1_ = 3.13, *g*_2_ = 1.90, *g*_3_ = 1.98. Hyperfine coupling to a single Co nucleus with *A*_1_ = 320 MHz, *A*_2_ = −85 MHz, *A*_3_ = 100 MHz, peak-to-peak line width parameters are 20 and 20 G for Gaussian and Lorentzian contribution, respectively. To reproduce the asymmetric line shape, a distribution of *g* and *A* values was assumed (see text). Simulation performed with pepper routine of EasySpin.^39^.

### Computational Electronic Structure Analysis

For the anion [LCo_2_(N_2_)]^−^, density functional theory calculations (BP86/ZORA-def2-TZVP, see SI for details) predict a closed-shell singlet state as the ground state, with the lowest triplet state ca. 10 kcal/mol higher in Gibbs free energy. The geometry of the singlet state reproduces the structure of the anions of **2** and **3** well, whereas a significantly worse match is obtained for a fully relaxed geometry with a triplet electronic structure (distances of singlet and triplet, Co···Co: 4.016 Å and 3.949 Å, Co–N: 1.775 Å and 1.820 Å, N–N: 1.158 Å and 1.181 Å; angle Co-Ct_N2_-Co: 121.8° and 114.7°, see SI for details). Positioning one or two potassium cation(s) with explicit THF solvent molecules above the pyrazolate as in the crystal structure of **2** has marginal effects on the obtained relaxed structures.

The experimental UV–vis spectrum of solid **3** as well as of **2** and **3** in THF solution (where in case of **2**, K^+^ is assumed to be solvent separated) shows a broad absorption at around 590 nm. All attempts to predict the UV–vis spectrum with time-dependent DFT with and without spin–orbit coupling failed (see SI for details). The calculated UV–vis spectrum of the closed shell singlet state misses the characteristic absorption peak in the region of 590 nm. One reason for this discrepancy might be the known difficulties of TD-DFT in reproducing charge-transfer transitions.^40^

A calculation of the fractional occupation number weighted electron density (FOD),^41^ which captures static electron correlation effects, shows significant delocalization at the Co and N centers (see SI for details). We were thus prompted to revisit the electronic structure of [LCo_2_(N_2_)]^−^ with wave function approaches. Coupled cluster calculations (using the DLPNO approach) confirm that the singlet state is strongly stabilized (see SI) with no indication of multiconfigurational character. A further evaluation of the electronic structure used complete active space calculations with subsequent *N*-electron valence perturbation theory considering spin–orbit coupling effects (SOC-CASSCF/NEVPT2). To design an appropriate active space, the unusual bonding situation of the bent Co–N–N–Co unit in **2**–**4** was analyzed. In the more common linear M–N–N–M binding motifs, the five metal d-orbitals and three nitrogen p-orbitals form a set of four σ-, eight π- and four δ-orbitals with 0–3 nodal planes.^6,42^ In a hypothetical linear Co^I^–N–N–Co^I^ system with local square-planar coordination environments, the qualitative MO diagram has split d­(xz)/d­(yz)-dominated orbitals since only the d­(xz) orbitals can interact with the dinitrogen π-orbitals whereas the d­(yz) orbitals are nonbonding with respect to the N_2_ unit. The second set of π-orbitals is formed with the d­(xy) orbitals, see Figure 8a, that are much more destabilized in a square-planar ligand field than for instance in an octahedral ligand field. Upon bending the Co–N–N–Co unit, the overlap of some metal d-orbitals with the nitrogen p-orbitals decreases. The SOC-CASSCF/NEVPT2 calculations indicate that the electronic structure of [LCo_2_(N_2_)]^−^ is not a simple closed shell singlet, as has been observed in another recently published N_2_-bridged dichromium complex.^43^ The multireference ground state predicted for the dicobalt complex at this level of theory is the result of spin–orbit coupling between singlet and triplet configurations that themselves have multiconfigurational character. The lowest-lying singlet roots have singly occupied orbitals of d­(z^2^) and (π*-N_2_)_ip_ character (see SI for details). Clearly, a closed-shell singlet ground state as predicted by single-reference DFT is an extremely poor representation of the multireference electronic structure found at the CASSCF level of theory. At the same time, the open-shell nature of the lowest singlet roots makes transparent why all attempts to obtain a broken-symmetry solution failed: there is significant overlap between the metal and nitrogen orbitals carrying the unpaired electrons, thus facilitating a collapse of the broken-symmetry wave function to the closed-shell solution during the SCF cycles. A dedicated quantum chemistry study will be needed to further elucidate which level of theory best represents the electronic structure of [LCo_2_(N_2_)]^−^, which is potentially more complex than a simple closed-shell singlet.

**Figure 8:**
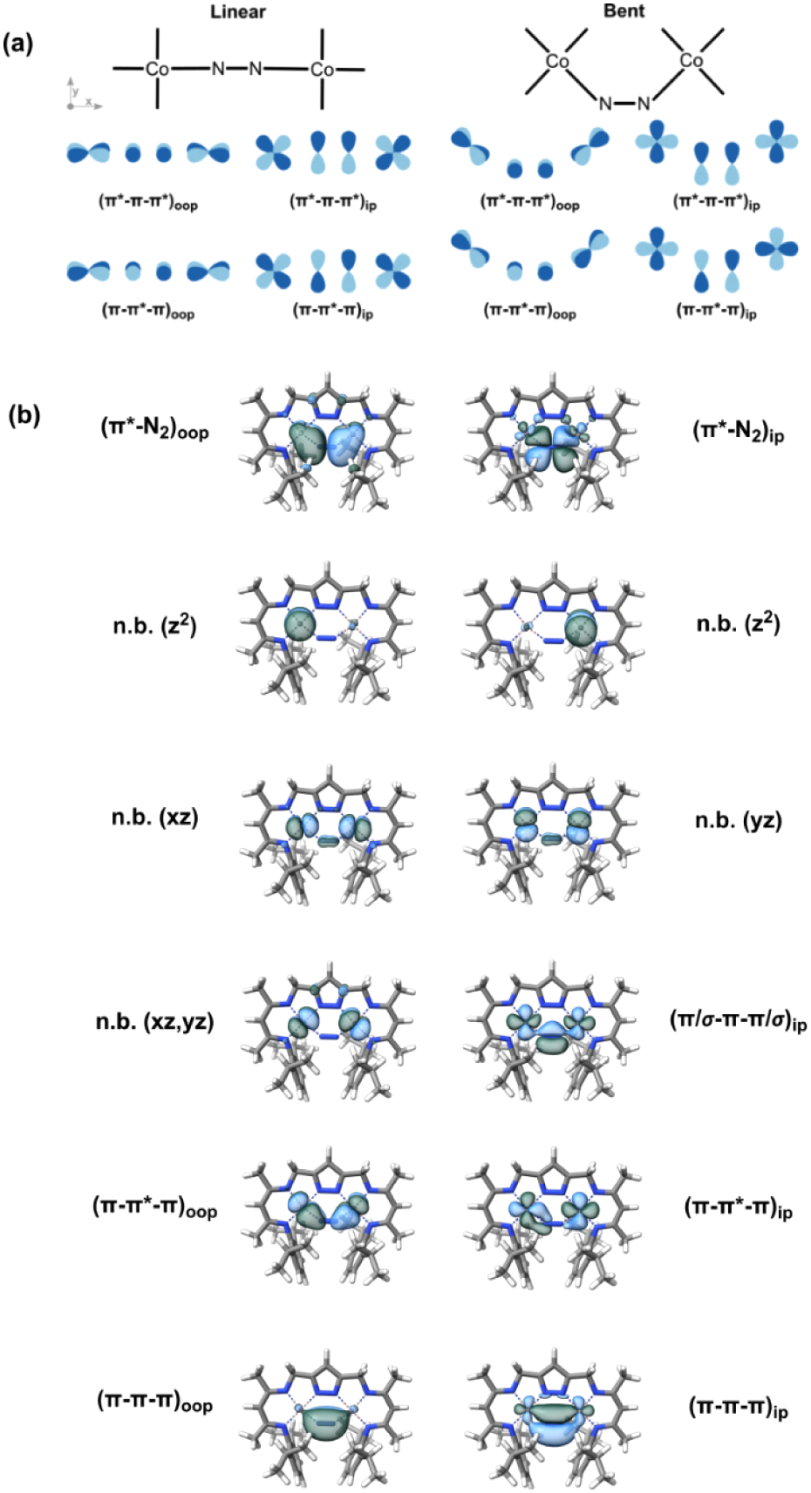
(a) Qualitative partial molecular orbital diagrams for Co^I^ in a square-planar environment in a linear (left) and bent (right) Co^I^–N–N–Co^I^ unit; shown are representative AO interactions of the π-system. (b) Active space orbitals of CASSCF calculations with 20 electrons in 12 orbitals, (20, 12), for complex [LCo_2_(N_2_)]^−^.

For the neutral, mixed-valent complex **6**, CASSCF/NEVPT2 calculations using the crystal structure with relaxed hydrogen atom positions indicate a complicated electronic structure for which no suitable active space for a detailed quantum chemical analysis could be identified so far (see SI for details).

### Chemical Properties of the Dicobalt Dinitrogen Complexes

All dicobalt dinitrogen complexes reported in this work, **2** – **4** and **6**, are very air and moisture sensitive. In preliminary reactivity studies we have examined possible hydride transfer onto the constrained μ_1,2_-N_2_ substrate in **2** (it should be noted though that the intrinsic properties of the bent μ_1,2_-N_2_ substrate may be partly concealed by the binding of a second N_2_ molecule to give **5** in solution, vide supra). To that end, complex LCo_2_Br (**1**) in THF solution under N_2_ was treated with an excess of KBEt_3_H, generating **2** in situ. From the reaction mixture the unusual adduct [LCo_2_(N_2_)]­[K­(THF)_2_]·KBEt_3_H (**2**(THF)·KBEt_3_H) could be isolated in crystalline form. While the quality of the crystallographic structure determination is moderate and does not allow for detailed discussion, the constitution of **2**(THF)·KBEt_3_H is clearly established (see Figure S57). It shows that the core structure of **2**, viz. the anion [LCo_2_(N_2_)]^−^ with a K^+^ located above the pyrazolate ring, is retained, but a second K^+^ is found above a BDI subunit and these entities are now linked via two [BEt_3_H]^−^ anions. The presence of superhydride anions besides the intact [LCo_2_(N_2_)]^−^ complex (*ν̃*_NN_ = 1914 cm^–1^ for **2**(THF)•KBEt_3_H; see Figure S20) indicates that the strong hydride donor [BEt_3_H]^−^ (Δ*G*°_H–_ = 26 kcal/mol)^44^ is not sufficient for hydride transfer onto the bent dinitrogen ligand in **2**, and that the putative [LCo_2_(N_2_H)] ^2^− should have an even lower hydricity.

When reacted with KC_8_ and Me_3_SiCl (2000 equiv. each) under N_2_ atmosphere at –80 °C in THF for 2 h followed by stirring for 22 h at rt, **2** produces around 200 equiv of N­(SiMe_3_)_3_ (or NH_4_^+^ after acid hydrolysis, 30.5% yield with respect to Me_3_SiCl). The yields of N­(SiMe_3_)_3_ are comparable with the ones reported for a cyclophane-based Co_3_ complex (a precursor of **V**)^45^ or the pyrazolato-based dicobalt complex **VI** (cf. Figure 1)^31^ as well as for the most active mononuclear Co catalysts for this silylation reaction,^46,47^ demonstrating that the present constrained dicobalt scaffold is suited for onward reductive activation leading to N–N bond cleavage. Direct production of NH_3_ by treating **2** with a suitable combination of reductant and acid is challenging because of the sensitivity of **2** toward commonly used acids such as [HLut]­BAr^F^_4_ (BAr^F^_4_^–^ is [B­(C_6_H_3_(CF_3_)_2_-3,5)_4_]^−^) or [HPCy_3_]­PF_6_, possibly because of backbone protonation at the β-diketiminato subunits of the ligand scaffold, as observed earlier for dinickel complexes of the same ligand.^26b^ Further reactivity studies with **2** and **6** are ongoing.

## Summary and Conclusions

This report details the synthesis and characterization of a set of novel dicobalt dinitrogen complexes **2**–**4** and **6** where the N_2_ substrate is found in an unusually bent bonding situation featuring the most acute M–Ct_N2_–M angles for dinuclear μ_1,2_-N_2_ complexes reported to date; **2**–**4** and **6** also represent the first dicobalt complexes with a μ_1,2_-N_2_ ligand in nonlinear geometry. This is enforced by the structural constraints imposed by the pyrazolato/BDI hybrid ligand that creates a preorganized pocket with rather fixed Co···Co separation of ∼ 4 Å for hosting N_2_ in a highly bent μ_1,2_ mode between the two Co ions. Geometrically the resulting structures closely resemble the bent μ_1,2_-bridging α-N_2_ intermediate that precedes N–N bond cleavage on the Fe(111) surface of the Haber-Bosch catalyst. This structural motif with acute Co-Ct_N2_-Co angle is retained even after 1e^–^ oxidation of the dicobalt­(I) systems (**2** – **4**) to the unusual mixed-valent Co^I^Co^II^ species **6**.

The bent Co–N–N–Co arrangement significantly decreases overlap of metal d-orbitals with the in-plane p­(N) orbitals, yet in the formal dicobalt­(I) complex anion [LCo_2_(N_2_)]^−^ the N_2_ is substantially activated with *ν̃*_NN_ ≈ 1900 cm^–1^; this value is significantly lower than in most Co^I^ complexes with end-on bound N_2_ and also lower than in most dicobalt­(I) dinitrogen complexes with the common linear Co–N–N–Co arrangement. A quite pronounced degree of N_2_ activation is also observed for mixed valent **6** if compared with known Co^II^/N_2_ complexes. Structural and spectroscopic data evidence that a K^+^ cation does not exert any significant influence on the degree of N_2_ activation in **2** – **4**, i.e., it does not act cooperatively. While [LCo_2_(N_2_)]^−^ is diamagnetic, electronic structure analyses with single- and multireference quantum chemistry methods show a potentially more complex picture. DFT and DLPNO–CC calculations yield a closed-shell singlet as the ground state, whereas SOC-CASSCF/NEVPT2 calculations indicate that the lowest-lying states may have multiconfigurational nature.

The analysis of [LCo_2_(N_2_)]^−^ shows that it is not straightforward to translate the high degree of M–N–N–M bending and the substantial N_2_ activation into specific onward reactivity of the bound N_2_ substrate, which also depends on the local ligand field splitting of the transition metals, the occupation of orbitals with bonding and antibonding character with respect to the N–N interaction, and the degree of covalency of the metal–nitrogen interaction. Regardless of the degree of activation, a more exposed dinitrogen unit may be more susceptible to onward reactivity, providing a strong motivation to further study bent M–N–N–M motifs. The in-plane π system in the bent unit is reminiscent of lone pairs. While proper, occupied lone pairs may enable protonation or reactions with other electrophiles, it remains to be demonstrated whether the type of complex presented here is more susceptible to electron donating reactants.

The bent M–N–N–M motif may be viewed as the beginning of a reaction path that is complementary to the “zigzag” transition states often invoked in thermal reactivity,^5^ or the computational suggestion by Reiher and coworkers^48,49^ to photochemically access a “zigzag” intermediate. In either case, the onset of lone pair formation is expected upon “trans-bending” the M–N–N–M unit, implying reactivity on opposite sides of the dinitrogen unit. The “cis-bending” in the present complexes [LCo_2_(N_2_)]^−^ (and prior diiron examples **I** – **IV** shown in Figure 1)^10–13^ prepares the dinitrogen unit for concerted reactivity from the same side. We thus suggest that the pronounced exposition of the extremely bent μ_1,2_-N_2_ unit in the present complexes [LCo_2_(N_2_)]^−^ may ready the system for novel onward reactivity, which is currently under investigation in our laboratories.

## Supplementary Material





## Notes

Experimental details and synthetic procedures, further spectroscopic and analytical data, crystallographic information, details of quantum chemistry calculations (PDF)
Cartesian coordinates (PDF)
