# Supplementary material for: Severely Bent Dinitrogen Bridging in Highly Preorganized Dinuclear Cobalt Complexes Featuring an Intricate Electronic Structure
Source: JACS Au. 2025 Jun 20;5(7):3104–14. doi: 10.1021/jacsau.5c00129 (PMC12308450; doi:10.1021/jacsau.5c00129)
Supplement: Supplementary file 1 [file au5c00129_si_001.pdf]

Supporting Information

for

Severely Bent Dinitrogen Bridging in Highly Preorganized Dinuclear Cobalt Complexes Featuring an Intricate Electronic Structure

Yue Wang,[†] *Shweta Singh,*[‡] *Andreas Meyer,*[#,$] *Sebastian Dechert,*[†] *Sandeep K. Gupta,*[†,%] *Serhiy Demeshko,*[†] *Vera Krewald,*[‡,*] *Franc Meyer*[†,§,*]

[†] University of Göttingen, Institute of Inorganic Chemistry, Tammannstrasse 4, D-37077 Göttingen, Germany

[‡] Technische Universität Darmstadt, Fachbereich Chemie, Quantenchemie, Peter-Grünberg-Str. 4, D-64287 Darmstadt, Germany

[#] Max Planck Institute for Multidisciplinary Sciences, Am Fassberg 11, D-37077 Göttingen, Germany

[$] University of Göttingen, Institute of Physical Chemistry, Tammannstrasse 6, D-37077 Göttingen, Germany

[%] Department of Chemistry, Indian Institute of Technology Delhi, 110016, New Delhi, India

[§] University of Göttingen, International Center for Advanced Studies of Energy Conversion (ICASEC), D-37077 Göttingen, Germany

* To whom correspondence should be addressed:
Email: franc.meyer@chemie.uni-goettingen.de
vera.krewald@tu-darmstadt.de

Content

1 Methods and Instruments S2
2 Synthetic Procedures S4
3 NMR Spectra S7
4 IR and UV-vis Spectra S14
5 Magnetic Measurements and EPR Spectroscopy S23
6 Electrochemical Data S26
7 Computational Details and Additional Computational Data S27
8 Crystallographic Details S46
9 References S53

1 Methods and Instruments

Air sensitive reactions were carried out under dry argon using standard Schlenk techniques, or in a glovebox (MBraun LabMaster) under dinitrogen atmosphere with less than 0.5 ppm O_2 and H_2O. Chemicals were purchased from commercial sources and used as received. Glassware was dried at 120°C prior to use. THF, diethyl ether, pentane and hexanes were dried over sodium in the presence of benzophenone; all solvents were distilled prior to use. Toluene was dried over a solvent purification system (SPS) from MBraun. THF-d_8 was also dried over potassium and filtered through a filter pad (pore size 0.45 μm), then stored in the presence of 3 Å molecular sieves. $^{15}N_2$ was vacuo transferred into a Schlenk-tube with 3 Å molecular sieves and stored over the molecular sieves for at least 3 days. Ligand H_3L was synthesized as reported previously.[1] All other chemicals and solvents were purchased from commercial suppliers and used without further purification.

UV-vis spectra of solution samples were recorded on an Agilent Cary 60 equipped with an Unisoku Cryostat (CoolSpek) and magnetic stirrer using quartz cuvettes equipped with a tube and a Young valve. All UV−vis samples were prepared in a glovebox and transferred out of the glovebox prior to the measurement. UV-vis spectra of solid samples were recorded with a Varian Cary 5000 spectrometer with a Praying Mantis™ diffuse reflection attachment equipped with a sample chamber with quartz windows (Harrick Scientific Products), and the samples were prepared as fine powders diluted by KBr. Spectra were analyzed by Cary Win UV software.

IR measurements of solution samples were performed with a Bruker Vertex 70 spectrometer using an OTTLE cell with KBr windows under dinitrogen atmosphere in THF at room temperature. IR measurements of solid samples were recorded with a Cary 630 FTIR spectrometer with Dial Path Technology in a glovebox under dinitrogen atmosphere, and spectra were analyzed by FTIR MicroLab software.

NMR spectra were recorded on Bruker Avance 300, Avance 400 and Avance Neo 600 instruments. Chemical shifts (δ) are given in ppm and are referenced to the solvent residual signals. The peaks are labeled according to their splitting patterns with s (singlet), d (doublet), t (triplet), or m (multiplet).

Elemental analyses were performed by the Analytical Laboratory of the Institute of Inorganic Chemistry at the Georg-August-University Göttingen using an Elementar Vario EL III instrument. Deviations from the expected elemental analysis data, in particular the slightly low values for N, may results from the presence of trace amounts of LCo_2Br (**1**) in the samples of dinitrogen complexes **2** – **5** and **6** and/or from the high sensitivity of the compounds.

Temperature-dependent magnetic susceptibility measurements were carried out with a Quantum-Design MPMS3 SQUID magnetometer equipped with a 7.0 T magnet in the range from 295 or 210 to 2 K at an applied magnetic field of 0.5 T. In case of **1**, the polycrystalline powdered sample was packed in a polycarbonate capsule and covered

with low viscosity perfluoropolyether-based inert oil Fomblin Y45 to prevent torquing in a non-magnetic sample holder. Each raw data file for the measured magnetic moment was corrected for the diamagnetic contribution of the sample holder and the polycarbonate capsule. The raw data were corrected for the diamagnetic contribution of the capsule including the inert oil if used according to $M_{dia} = \chi_g \cdot m \cdot H$, with an experimentally obtained gram susceptibility of the capsules including the inert oil. The corrected experimental data for complex **1** was modelled using the *julX* program[2] by applying a fitting procedure to the Heisenberg-Dirac-van-Vleck (HDvV) spin Hamiltonian

$$\hat{H} = -2J\hat{S_1}\hat{S_2} + g\mu_B\vec{B}(\vec{S_1} + \vec{S_2}) \quad \text{(eq. 1)}$$

Temperature-independent paramagnetism (TIP) and paramagnetic impurities (PI) were included according to $\chi_{calc} = (1 - PI)\cdot\chi + PI\cdot\chi_{mono} + TIP$.

Intermolecular interactions in case of **6** were considered in a mean field approach by using a Weiss temperature Θ.[3] The Weiss temperature Θ (defined as $\Theta = zJ_{inter}S(S + 1)/3k$) relates to intermolecular interactions zJ_{inter}, where J_{inter} is the interaction parameter between two nearest neighbor magnetic centers, *k* is the Boltzmann constant (0.695 $cm^{-1}\cdot K^{-1}$) and *z* is the number of nearest neighbors.

The EPR sample of **6** (0.2 mM in 250 μL toluene) was prepared in a glovebox under N_2 atmosphere at room temperature, and then sealed in a *J*-Young EPR tube. All EPR measurements were conducted with an X-band (ca. 9.5 GHz) cw-EPR spectrometer (Bruker Elexsys E500 with a Bruker super-high Q resonator ER4122SHQE) at T = 12 K. The temperature was kept constant using an EPR900 cryostat from Oxford instruments with helium cooling. The modulation amplitude was set to 4 G at a microwave power of 10.02 mW. Small background signals were removed from the signal shown in Figure 7 by measuring an EPR spectrum of the empty resonator under identical conditions and subtracting this background signal. Simulations of the EPR spectra were conducted using matrix-diagonalization implemented in the pepper-routine of EasySpin (version 5.2.36)[4] in the software MATLAB. All simulation parameters are specified in the caption to Figure 7 in the main text.

Cyclic voltammograms were measured using a Perkin Elemer 263A potentiostat controlled by electrochemistry Powersuite software, a Gamry Reference 600 controlled by the Gamry Framework program. A three-electrode arrangement with a glassy carbon working electrode, a Pt reference electrode and a Pt wire/disc counter electrode was used. All CV experiments were performed in a glovebox under an atmosphere of dry dinitrogen in THF / 0.2 M $[^nBu_4N]PF_6$. The resulting data were converted to the Fc^+/Fc scale by using Cp_2Fe, added as internal standard after the experiment.

2 Synthetic Procedures

Synthesis of LCo_2Br (1)

A solution of KHMDS (98 mg, 0.49 mmol, 3.0 eq.) in THF (2 mL) was dropwise added to a solution of H_3L (100 mg, 0.16 mmol, 1.0 eq.) in THF (2 mL) at room temperature. The reaction mixture was stirred for 1 h and then added dropwise to $CoBr_2$ (70 mg, 0.32 mmol, 2.0 eq.) in THF (1.5 mL). The color changed directly from blue to dark red. The reaction mixture was kept at room temperature and stirred overnight. The red solution was filtered through a celite pad (1 cm) and the solvent was removed under reduced pressure. The residue was extracted with toluene and the insoluble parts were removed by filtration through celite (1 cm). Diffusion of hexanes into the toluene solution gave **1** as reddish needle-like crystals after one week (crystalline yield up to 46%); the crystals were suitable for X-ray diffraction. ^{1}H NMR (400 MHz, THF-d_8): δ = 134.6 (4H), 45.2 (1H), 27.5 (4H), 22.5 (2H), 2.6 (12H), -12.8 (12H), -24.5 (6H), -33.3 (6H), -98.8 (2H). ATR-IR (solid): $\tilde{\upsilon}$ (cm^{-1}) = 2956, 2924, 2865, 1529, 1458, 1434, 1391, 1380, 1366, 1330, 1312, 1279, 1259, 1249, 1220, 1186, 1173, 1152, 1129, 1110, 1109, 858, 796, 771, 761, 751, 550. Anal. Calcd. for $C_{39}H_{53}N_6BrCo_2$: C, 58.29; H, 6.65; N, 10.46. Found: C, 58.32; H, 6.57; N, 10.35.

Synthesis of $[LCo_2(N_2)]K(THF)$ (2)

A suspension of KH (4.0 mg, 0.10 mmol, 2.2 eq.) in THF (2.5 mL) was added to a THF (1 mL) solution of **1** (36.0 mg, 0.04 mmol, 1.0 eq.) at room temperature. The reaction mixture was kept stirring overnight. The color changed from red to purple. The reaction mixture was filtered through celite (1 cm). Needle-shaped crystals of **2** (4.5 mg, 55%; black appearance for large crystals, purple if crushed) were obtained via gradient crystallization from THF/hexanes (1:6 to 1:7); the crystals were suitable for X-ray diffraction. ^{1}H NMR (400 MHz, THF-d_8): δ = 6.7 (2H), 6.6 (4H), 5.4 (1H), 4.3 (2H), 4.1 (4H), 1.48 (6H), 1.3 (12H), 0.9 (16H). ATR-IR (solid): $\tilde{\upsilon}$ (cm^{-1}) = 2863, 1901, 1524, 1454, 1440, 1415, 1394, 1378, 1358, 1321, 1306, 1281, 1273, 1252, 1185, 1046, 1027. IR (solution in THF): $\tilde{\upsilon}$ (cm^{-1}) = 2040, 1907. Anal. Calcd. for $C_{43}H_{61}N_8OKCo_2$: C, 59.85; H, 7.93; N, 12.98. Found: C, 58.98; H, 7.10; N, 12.05.

Synthesis of $[LCo_2(N_2)]K(THF)_2(18c6)$ (3)

A suspension of KH (0.9 mg, 0.022 mmol, 2.2 eq.) in THF (1.5 mL) was added into a solution of **1** (8.0 mg, 0.01 mmol, 1.0 eq.) in THF (1 mL) at room temperature. The reaction mixture was kept stirring overnight. The color changed from red to purple. Then 18-crown-6 (1 M in THF, 12 μL, 0.012 mmol, 1.2 eq.) was added to the reaction mixture and stirring was continued for another 30 min. The color of the solution changed from purple to navy blue. The reaction mixture was filtered through celite (1 cm). Needle-shaped crystals of **3** (7.7 mg, 64%; black appearance for large crystals, dark blue if crushed)) were obtained via slow diffusion of Et_2O into the THF solution; the crystals were suitable for X-ray diffraction. ^{1}H NMR (400 MHz, THF-d_8): δ = 6.8

(2H), 6.7 (4H), 5.4 (1H), 4.4 (4H), 4.2 (2H), 3.7 (4H) 1.5 (6H), 1.4 (6H), 1.3 (6H), 1.0 (6H), 0.9 (6H), 0.7 (6H).$^{13}C\{^{1}H\}$ NMR (400 MHz, THF-d_8): δ = 154.7, 151.3, 148.9, 146.2, 122.9, 121.5, 99.2, 89.3, 70.2 (THF), 67.2 (THF), 52.9, 27.0, 25.4, 24.9, 23.4, 22.6, 22.3. ATR-IR (solid): $\tilde{\upsilon}$ (cm^{-1}) = 2953, 2899, 2862, 1905, 1519, 1452, 1402, 1380, 1350, 1322, 1305, 1277, 1249, 1188, 1132, 1100, 1057, 1025. IR (solution in THF): $\tilde{\upsilon}$ (cm^{-1}) = 1909. Anal. Calcd. for $C_{59}H_{93}N_8O_8KCo_2$: C, 59.08; H, 7.82; N, 9.34. Found: C, 58.19; H, 7.46; N, 8.73.

Synthesis of [$LCo_2(N_2)$]K(cryptand) (4)

Compound **4** was synthesized in a similar way as **3**, but using [2,2,2]-cryptand (4.4 mg, 0.012 mmol, 1.2 eq.) instead of 18-crown-6. Block-shaped crystals were collected for X-ray diffraction (black appearance for large crystals, dark blue if crushed). ^{1}H NMR (400 MHz, THF-d_8): δ = 6.8 (2H), 6.7 (4H), 5.4 (1H), 4.4 (4H), 4.3 (2H), 3.7 (4H), 1.5 (12H), 1.4 (6H), 1.0 (12H), 0.8 (6H). ATR-IR (solid): $\tilde{\upsilon}$ (cm^{-1}) = 2953, 2947, 2882, 2859, 2819, 2112, 2091, 2078, 2058, 1994, 1987, 1948, 1942, 1929, 1899, 1832, 1778, 1519, 1478, 1455, 1433, 1403, 1377, 1355, 1319, 1301, 1276, 1258, 1234, 1202, 1185, 1174, 1155, 1129, 1112, 1096, 1081, 1049, 1030, 1025. IR (solution in THF): $\tilde{\upsilon}$ (cm^{-1}) = 2050, 1907.

Preparation of 3^{15N2}

In a glove box filled with dinitrogen, **1** (8.0 mg, 0.01 mmol, 1.0 eq.) was dissolved in THF (2 mL). A suspension of KH (0.9 mg, 0.022 mmol, 2.2 eq.) in THF (1.5 mL) was added and the reaction mixture was kept in the dinitrogen glovebox overnight, giving a purple solution. The mixture was filtered through celite (1 cm), and to the filtrate was added 18-crown-6 (1 M in THF, 12 μL, 0.012 mmol, 1.2 eq.) to form a navy-blue colored solution. The solution was transferred into a 10 mL Schlenk-tube and transferred outside the glovebox. The reaction tube was conncected to the Schlenk line and the solution was frozen with liquid nitrogen. The head space was evacuated using Schlenk techniques, then flushed with Ar and again evacuated and flushed with Ar three times. Then $^{15}N_2$ was transferred into the evacuated Schlenk tube and the temperature of the reaction mixture was slowly raised to room temperature, leading to partial $^{14}N_2$/$^{15}N_2$ exchange. The Schlenk tube was transferred into an Ar glovebox and stirred overnight. Then the solvent was removed under reduced pressure. $\mathbf{3}^{15N2}$ (containing around 50% $^{14}N_2$ and $^{15}N_2$ each) was obtained as a black solid and used directly for IR measurements.

Synthesis of LCo_2N_2 (6)

LCo_2Br **1** (14.3 mg, 0.018 mmol, 1.0 eq.) was dissolved in 2 mL benzene and a suspension of KC_8 (3.4 mg, 0.025 mmol, 1.4 eq.) in benzene was added to the previous solution at room temperature. The reaction mixture was stirred vigorously overnight and resulted in a color change from red to dark brown. The reaction mixture was filtered through a filter pad and the solution was concentrated under reduced pressure. The

dark brown crystals of **6** (8.2 mg, 61%) suitable for singe crystal diffraction were obtained in a concentrated benzene solution. ^{1}H NMR (400 MHz, THF-d_8): δ = 88.4 (2H), 17.6 (4H), 16.2 (2H), 1.2 (4H), 1.48 (6H), 1.1 (12H), -0.8 (12H), -18.1 (6H), -22.3 (6H), -62.5 (1H). ATR-IR (solid): $\tilde{\upsilon}$ (cm^{-1}) = 2958, 2924, 2867, 1953, 1895, 1530, 1457, 1435, 1381, 1360, 1310, 1276, 1250, 1234, 1207, 1187, 1174, 1146, 1103, 1086, 1055, 1024. IR (solution in THF): $\tilde{\upsilon}$ (cm^{-1}) = 2002.

Preparation of $\mathbf{6}^{15N2}$

Isotope labeling of **6** was synthesized in a similar method as in the synthesis of $\mathbf{3}^{15N2}$. **6** was synthesized according to previous protocol in a N_2 glovebox. Subsequently the reaction mixture was transferred into a 10 mL Schlenk tube and brought out the glove box. The reaction mixture was connected to an Ar atmosphere Schlenk line, and the headspace was exchanged with Ar using freeze-pumping three times while the Schlenk tube merged in liquid nitrogen. Then headspace was then evacuated under static vacuum at same temperature and $^{15}N_2$ was then vacuum transferred into the reaction tube. As the reaction tube connected to $^{15}N_2$ the reaction was allowed to warm up to room temperature and the Schlenk tube was transferred into an Ar glovebox. The reaction mixture was stirred overnight. The solvent was removed under reduced pressure and the resulted black powder was suitable for IR measurements.

3 NMR Spectra

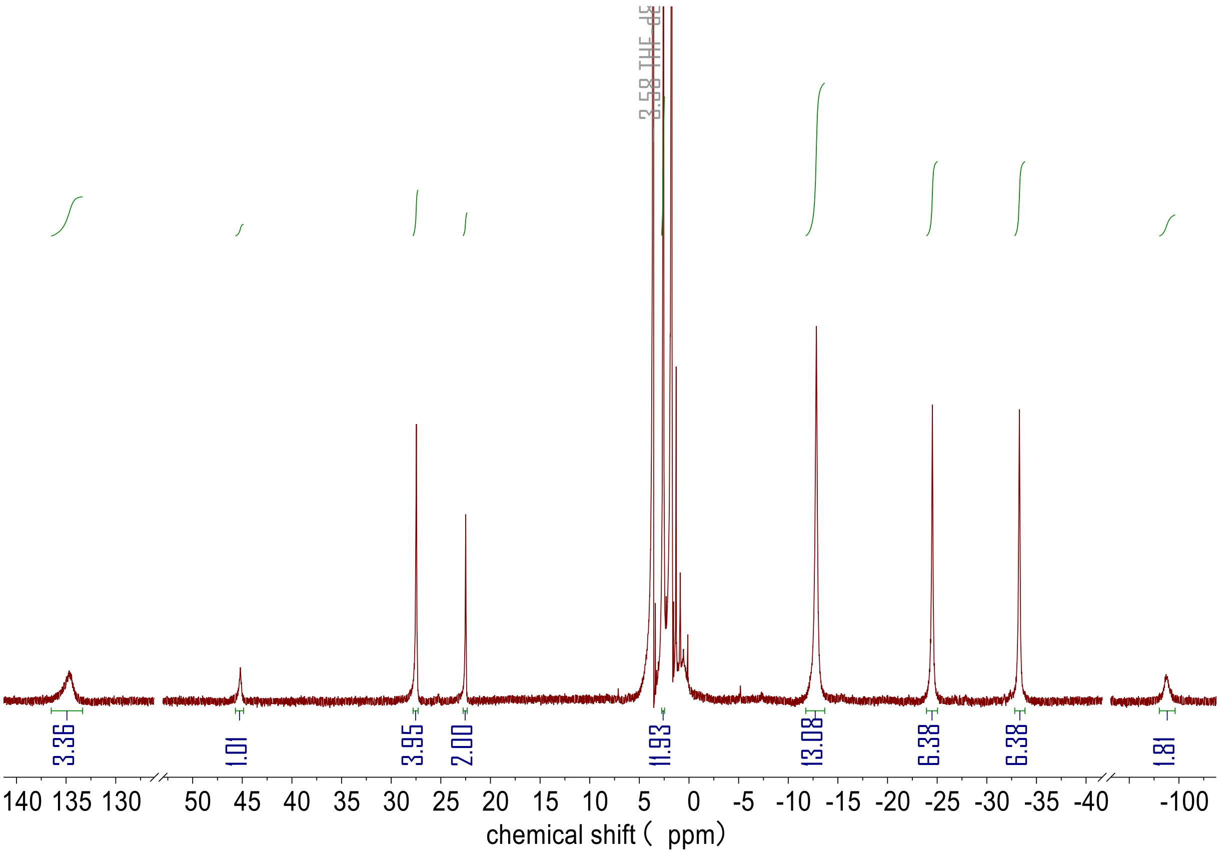

Figure S1. ^{1}H NMR spectrum of **1** in THF-d_8 at 298 K.

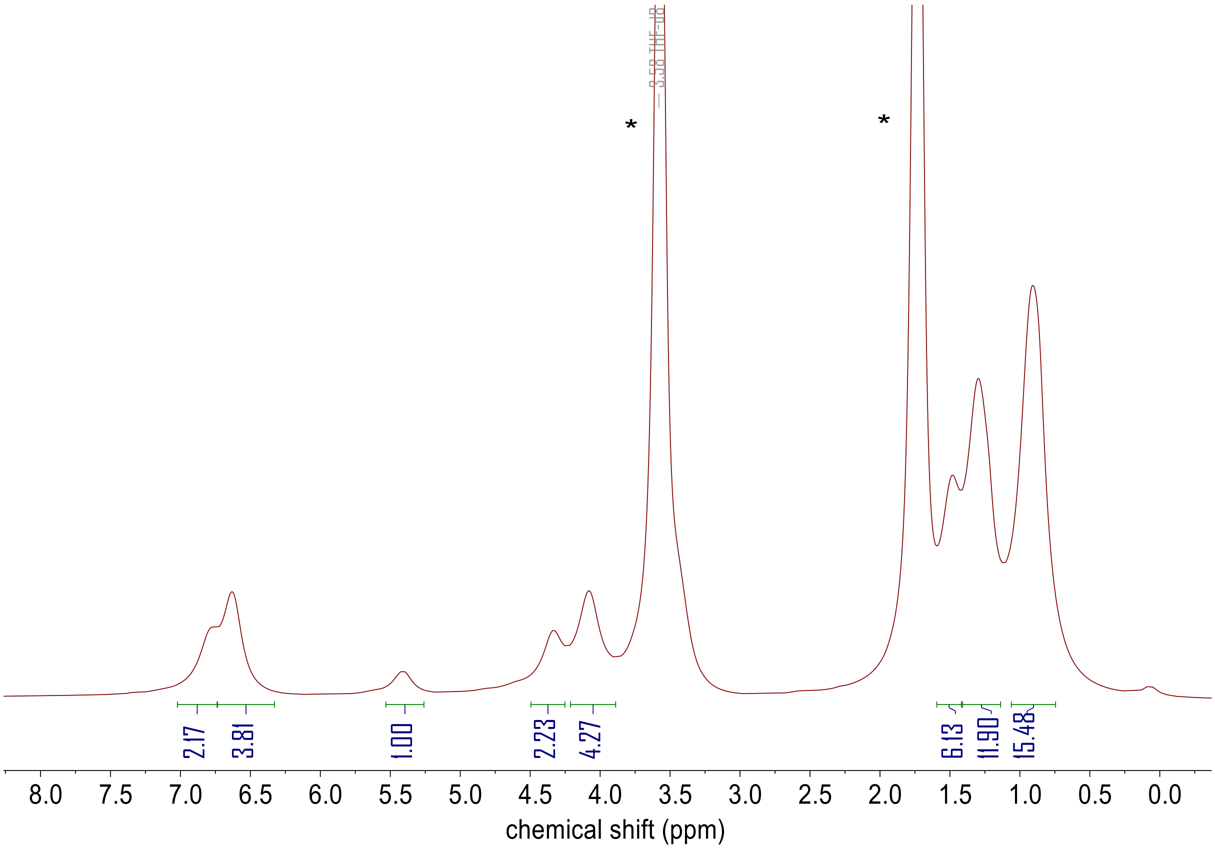

Figure S2. ^{1}H NMR spectrum of **2** in THF-d_8 at 193 K (solvent signals marked with *).

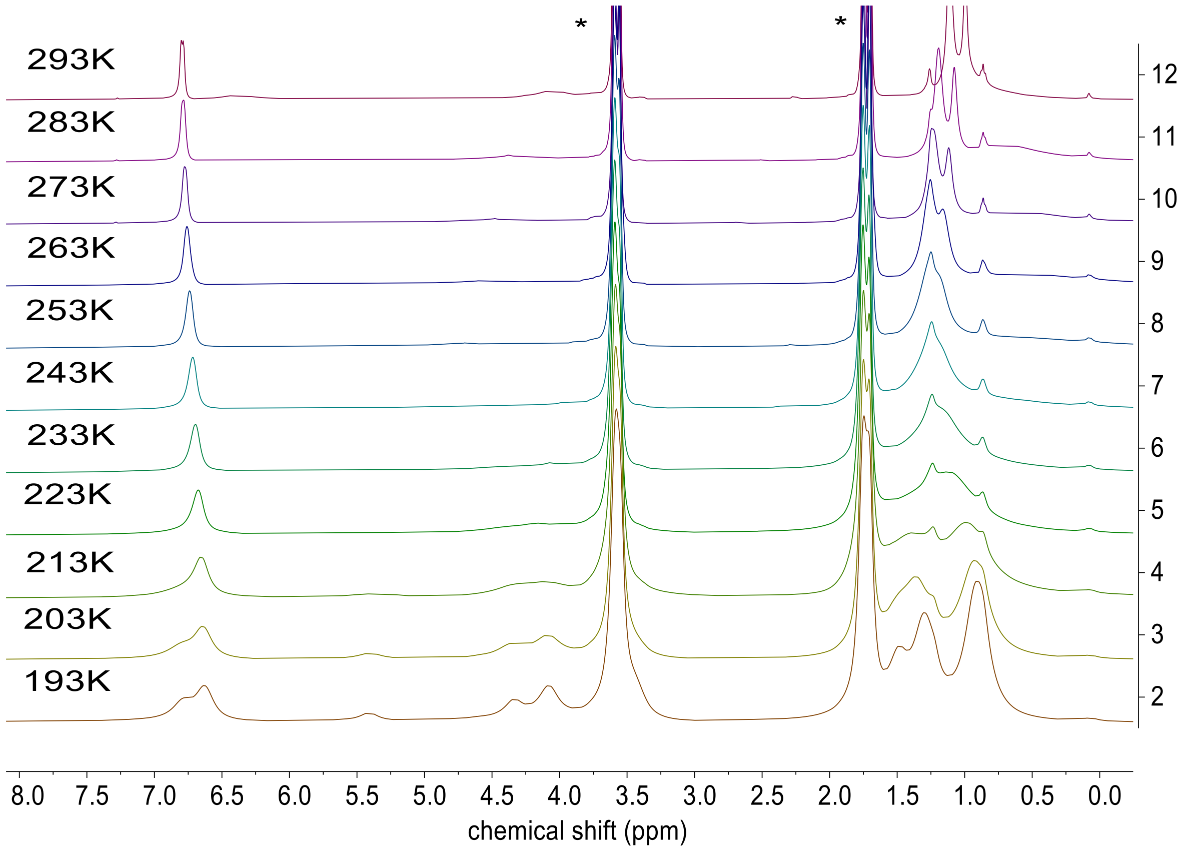

Figure S3. Variable temperature ^{1}H NMR spectra of **2** from 193 K to 298 K in THF-d_8 (solvent signals marked with *).

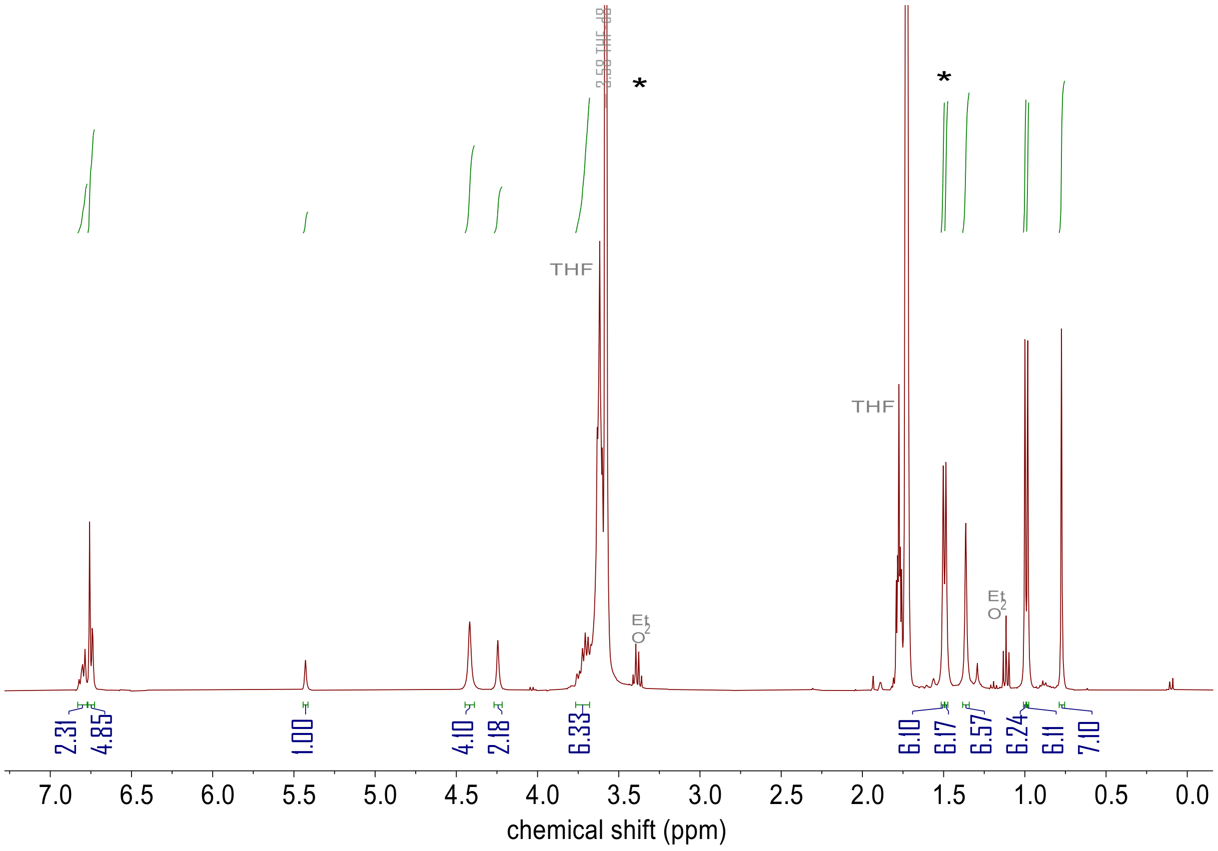

Figure S4. ^{1}H NMR spectrum of **3** in THF-d_8 at 298 K (solvent signals marked with *).

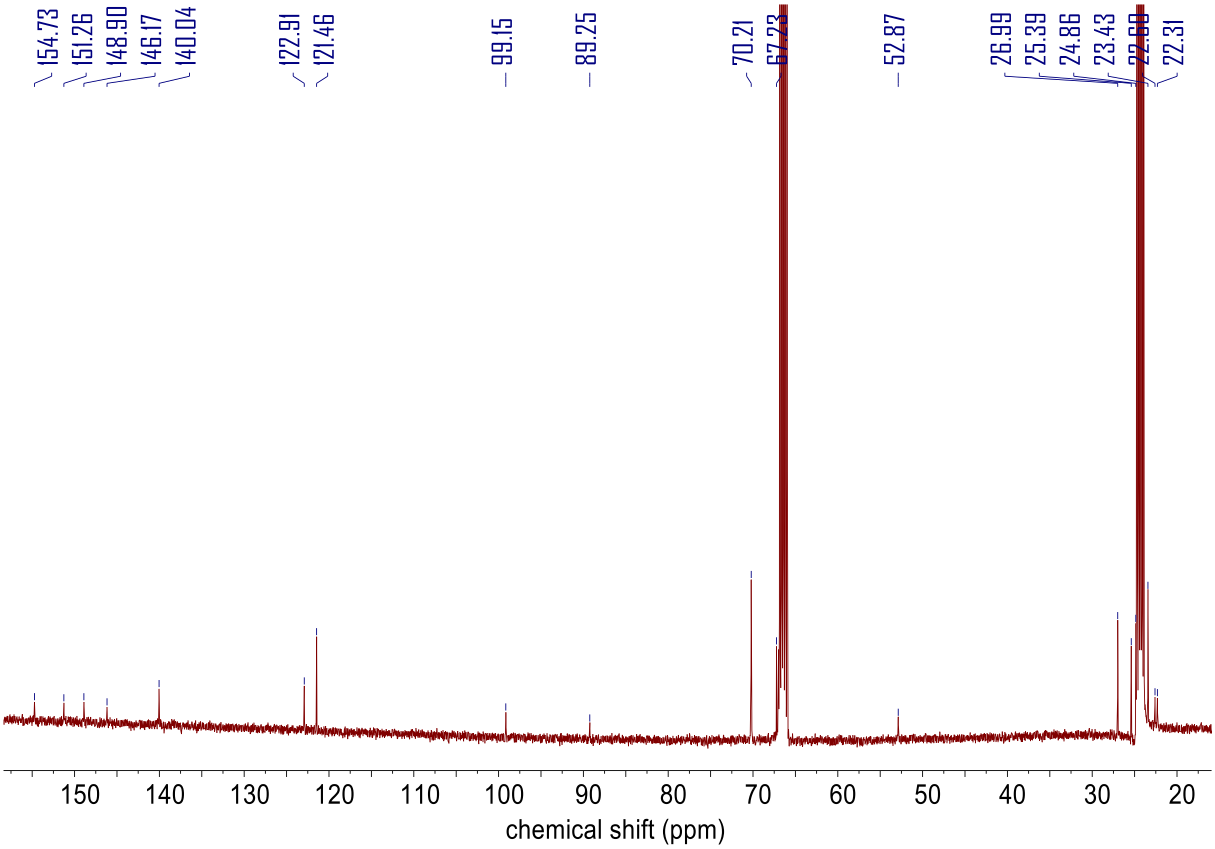

Figure S5. ^{13}C NMR spectrum of **3** in THF-d_8 at 298 K.

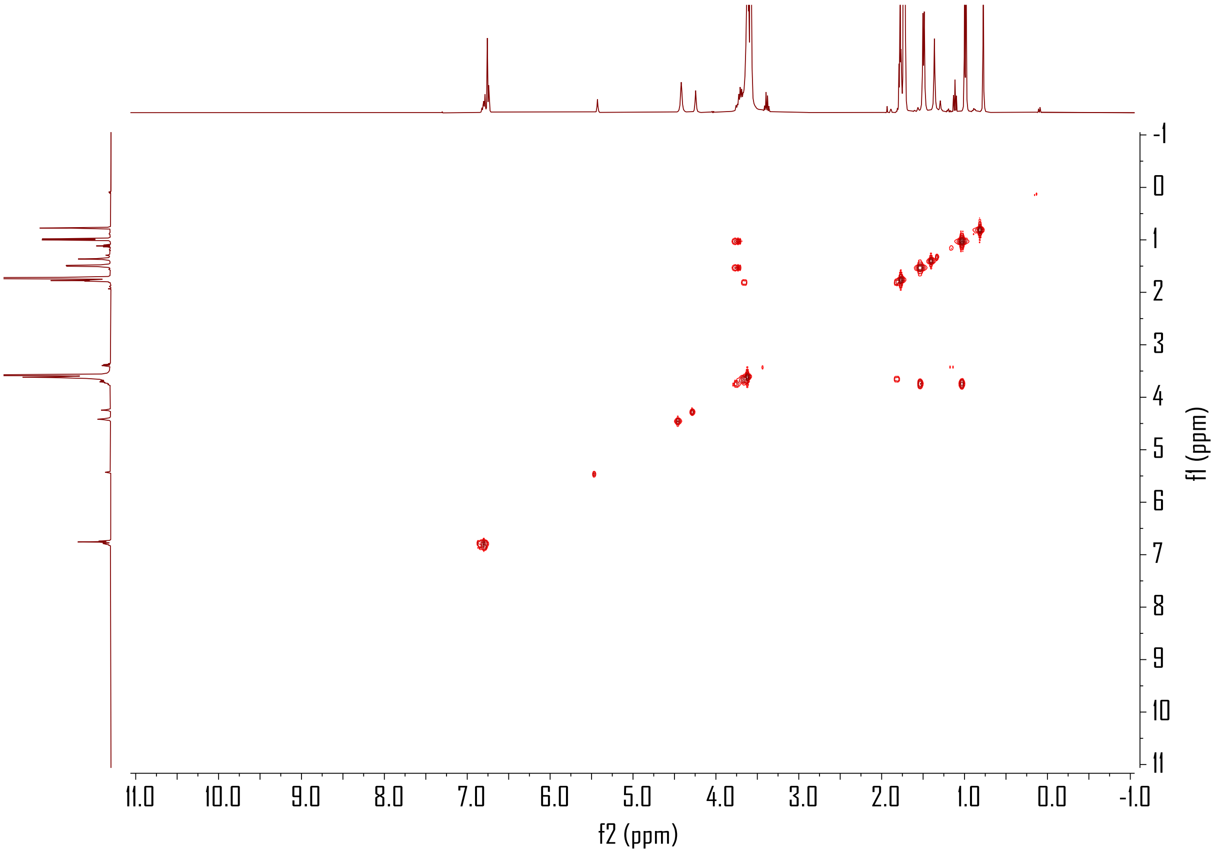

Figure S6. COSY NMR spectrum of **3** in THF-d_8 at 298 K.

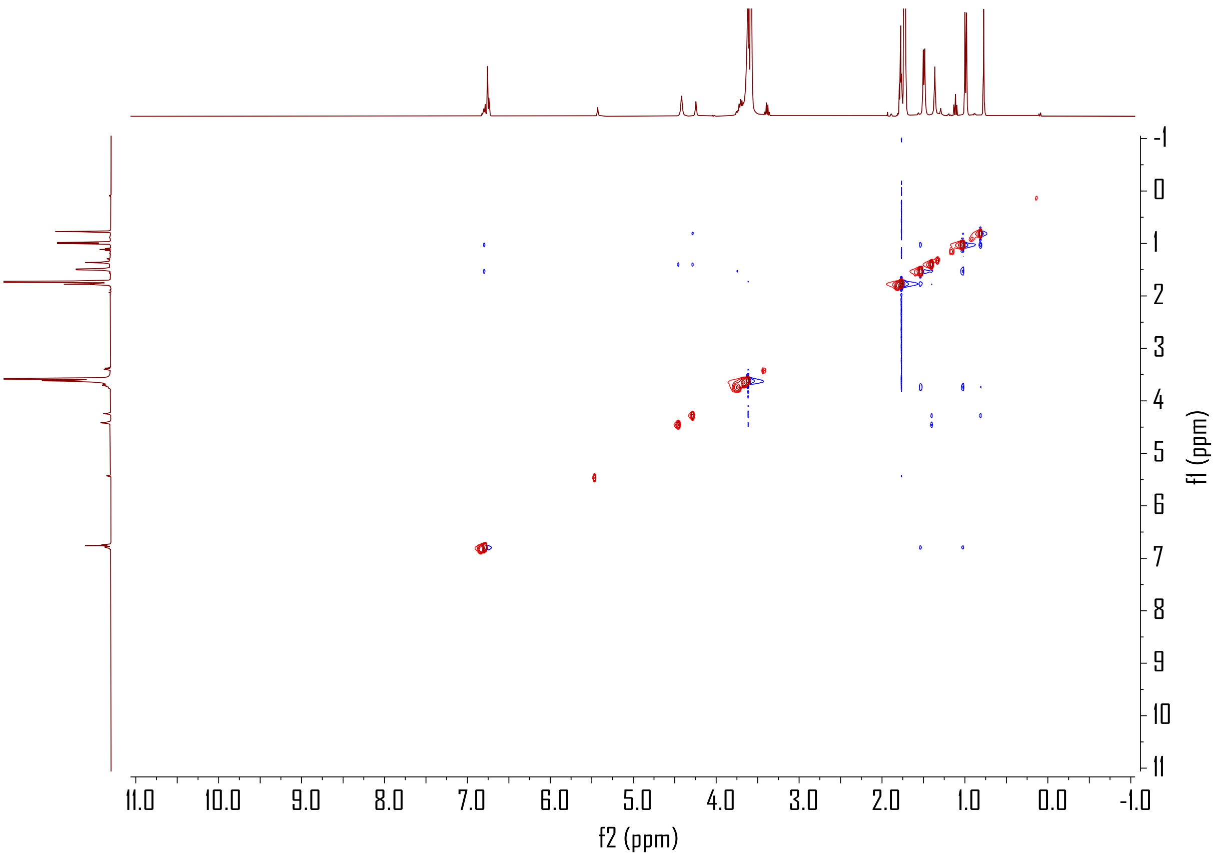

Figure S7. NOESY NMR spectrum of **3** in THF-d_8 at 298 K.

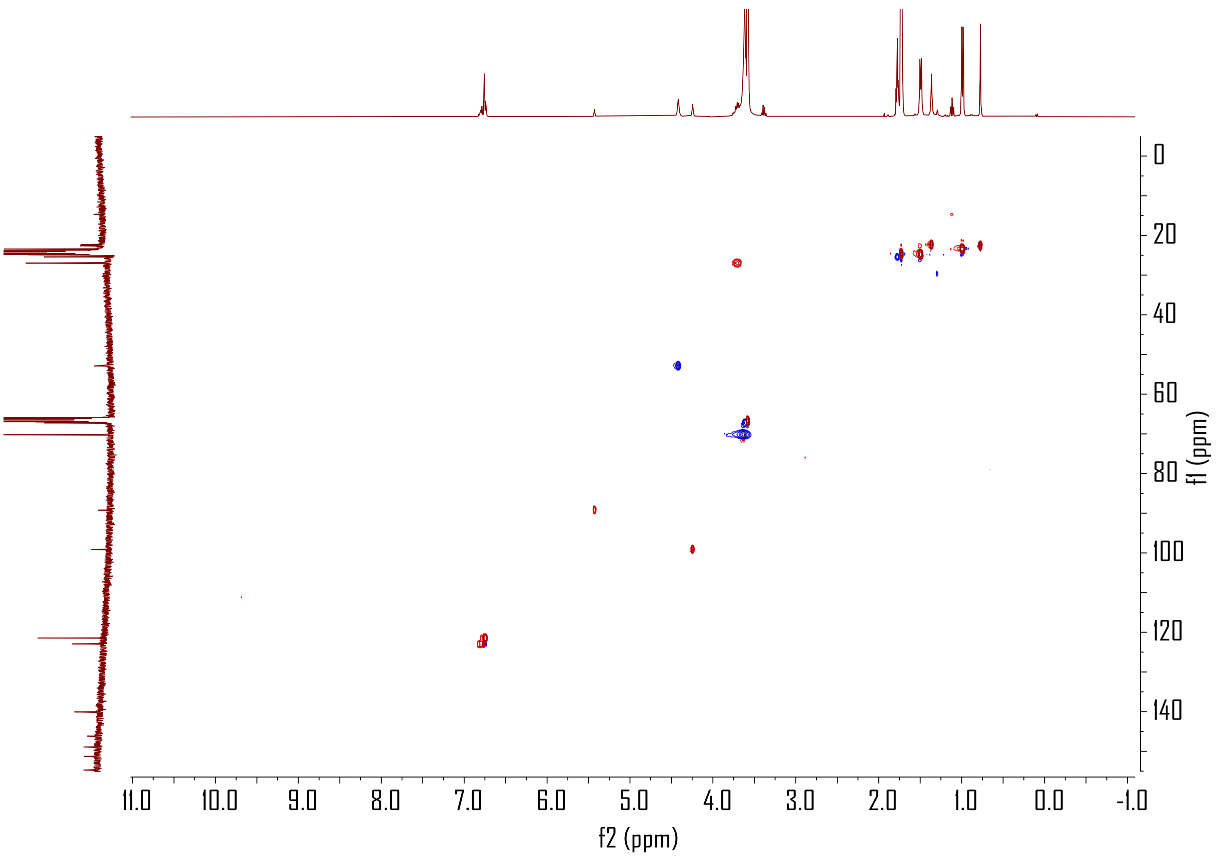

Figure S8. ^{13}C-HSQC NMR spectrum of **3** in THF-d_8 at 298 K.

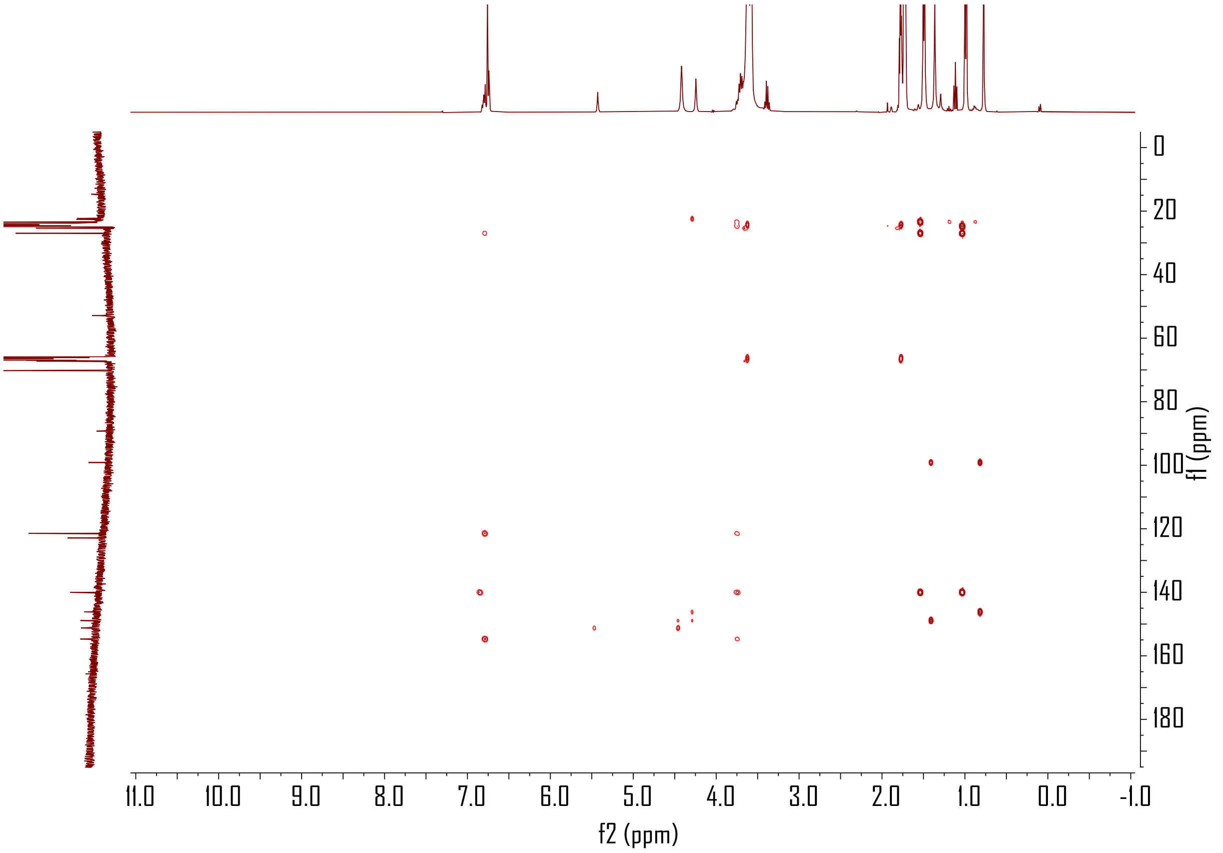

Figure S9. ^{13}C-HMBC NMR spectrum of **3** in THF-d_8 at 298 K.

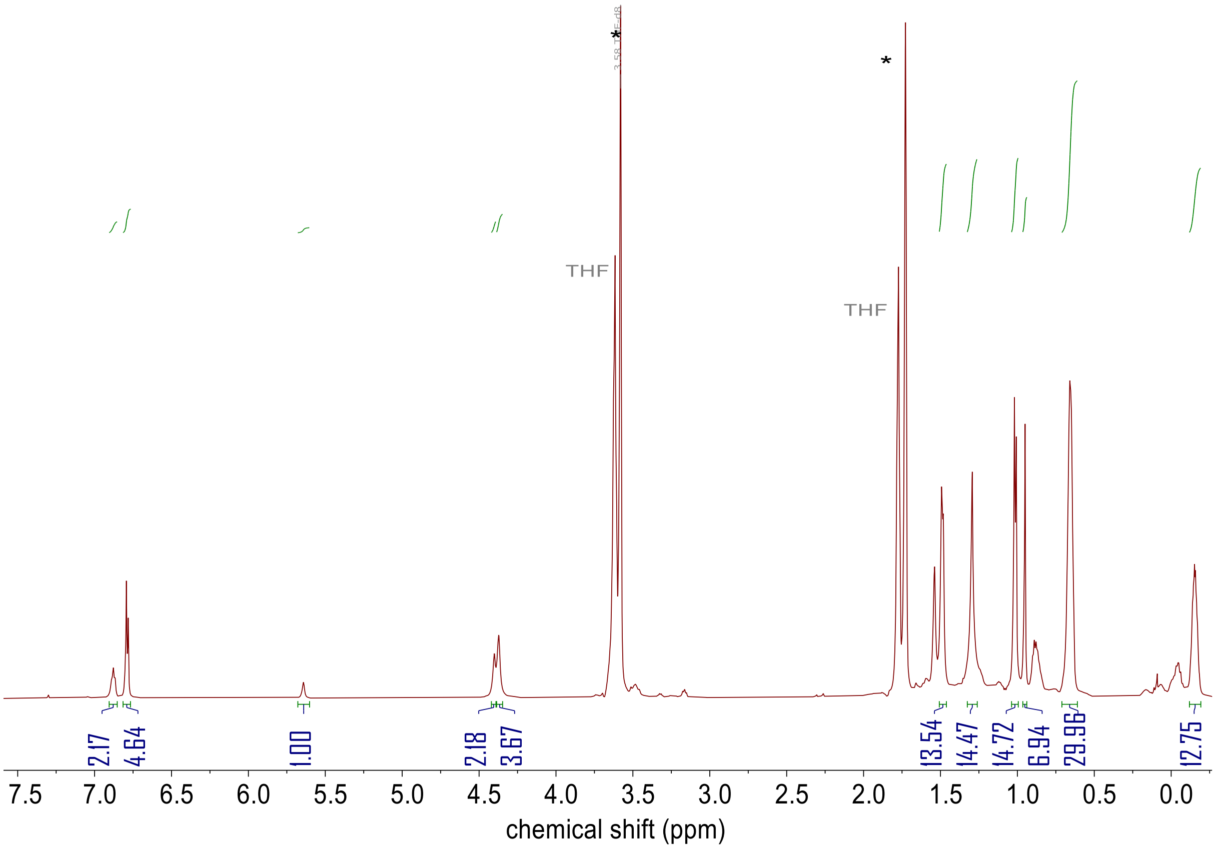

Figure S10. ^{1}H NMR spectrum of **2**(THF)·$KBEt_3H$ in THF-d_8 at 298 K (solvent signals marked with *).

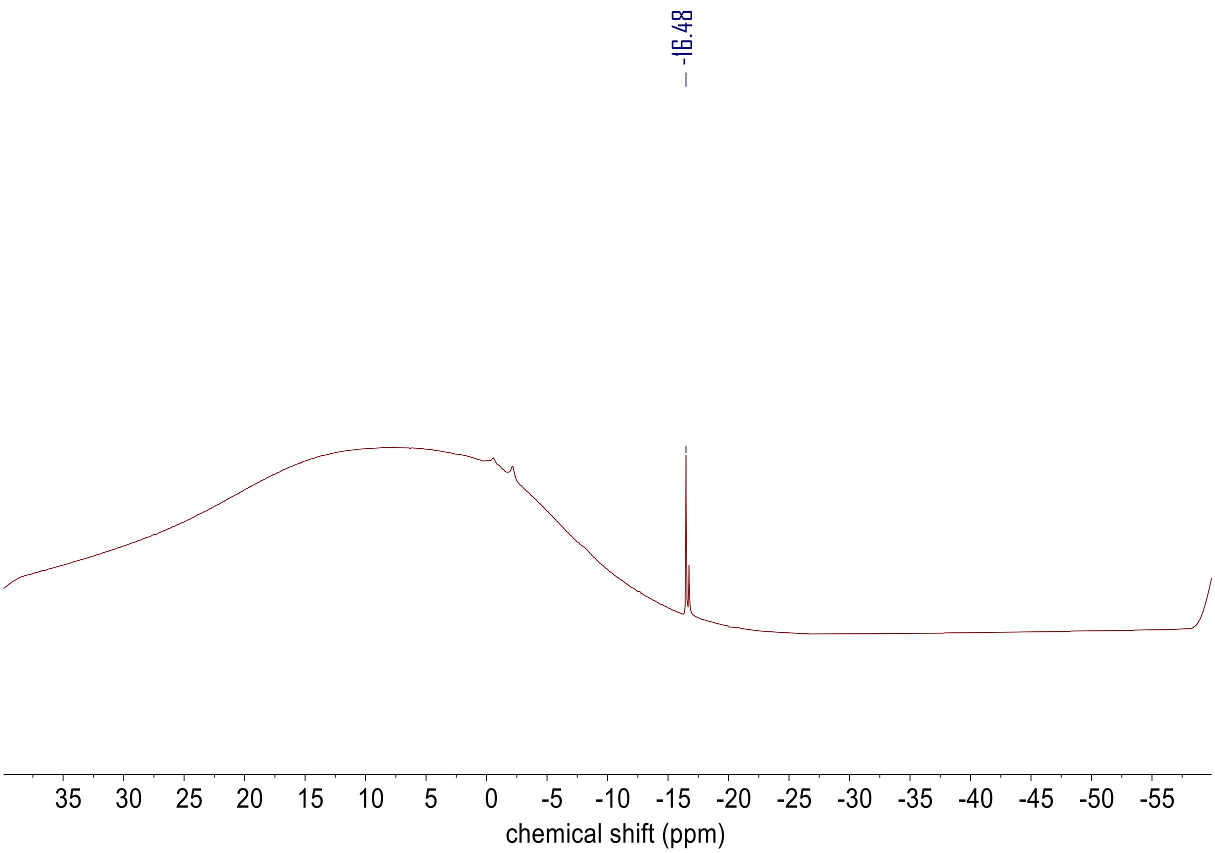

Figure S11. ^{11}B NMR spectrum of **2**(THF)·$KBEt_3H$ in THF-d_8 at 298 K.

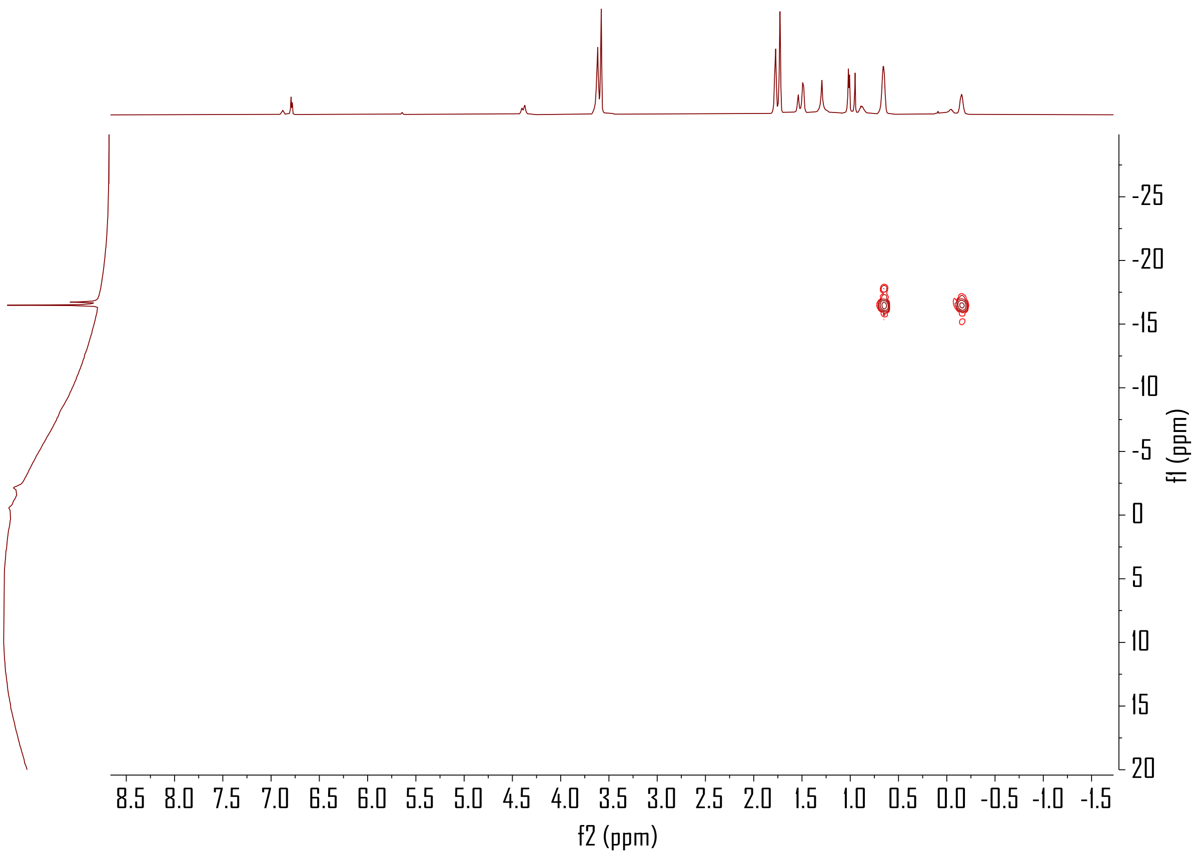

Figure S12. ^{11}B-H HMBC NMR spectrum of **2**(THF)·$KBEt_3H$ in THF-d_8 at 298 K.

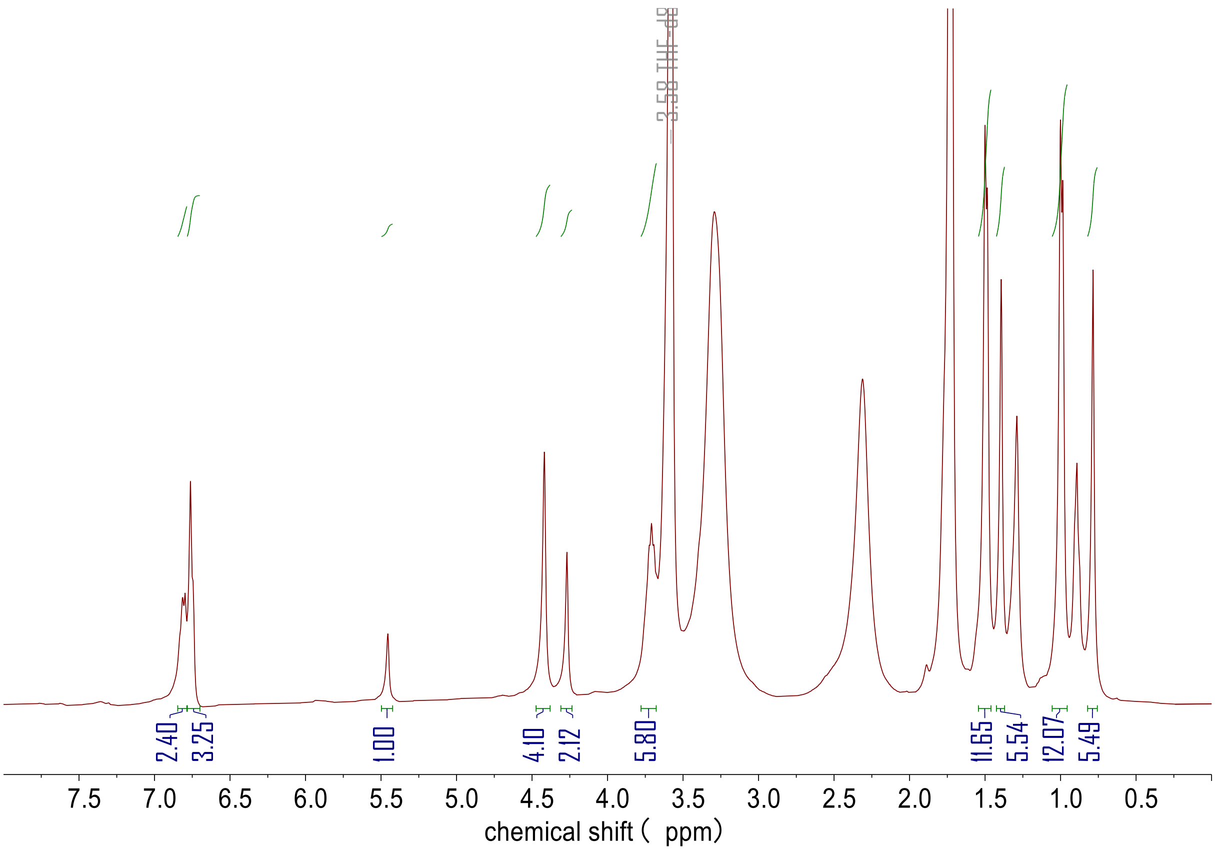

Figure S13. ^{1}H NMR spectrum of **4** in THF-d8 at 298 K.

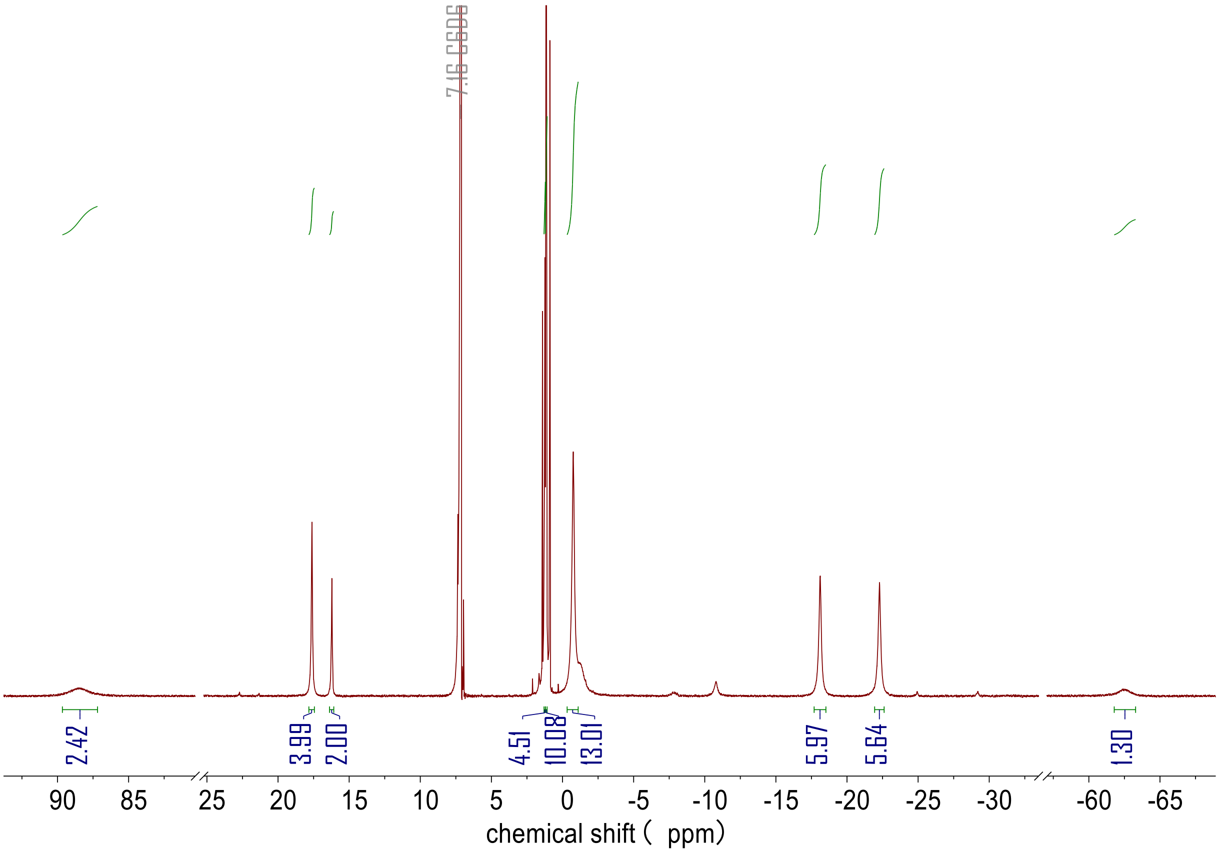

Figure S14. ^{1}H NMR spectrum of **6** in C_6D_6 at 298 K.

4 IR and UV-vis Spectra

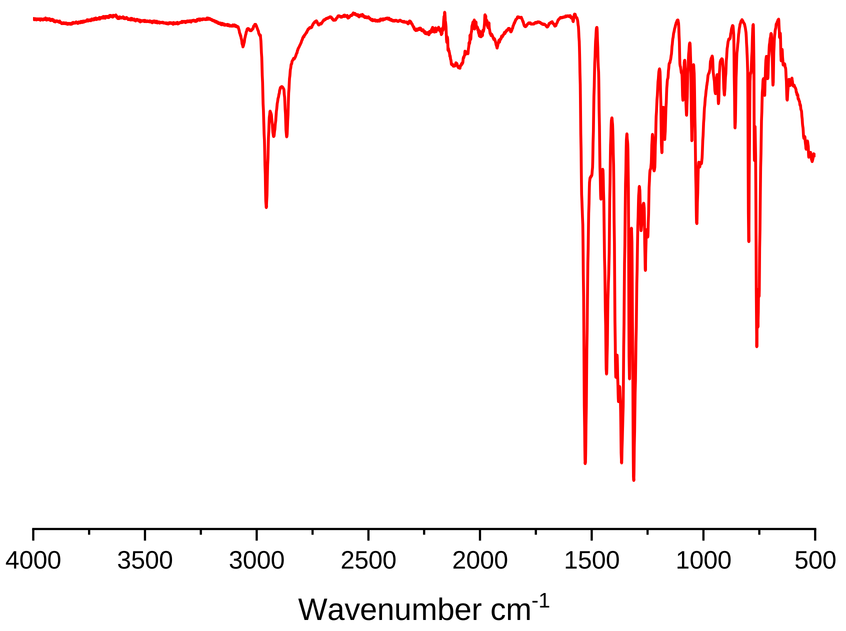

Figure S15. ATR-IR spectrum of solid **1**.

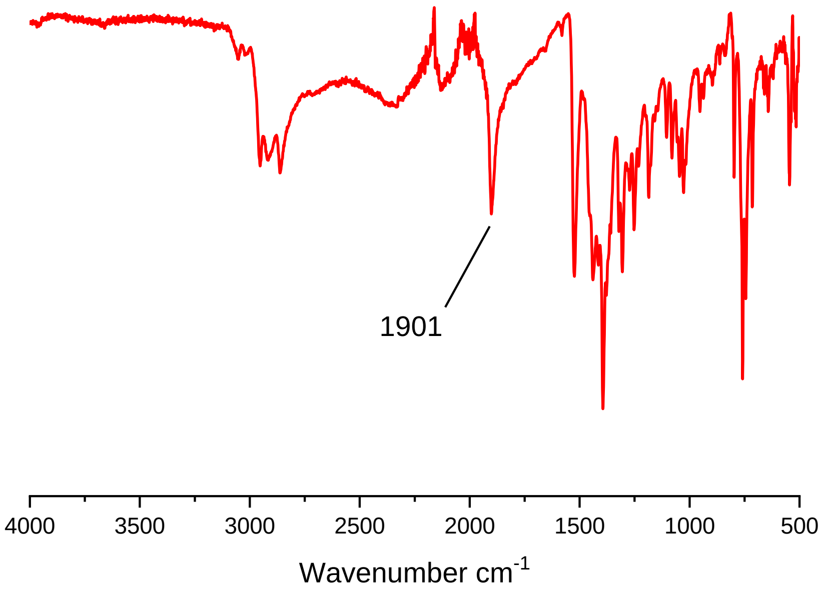

Figure S16. ATR-IR spectrum of solid **2**.

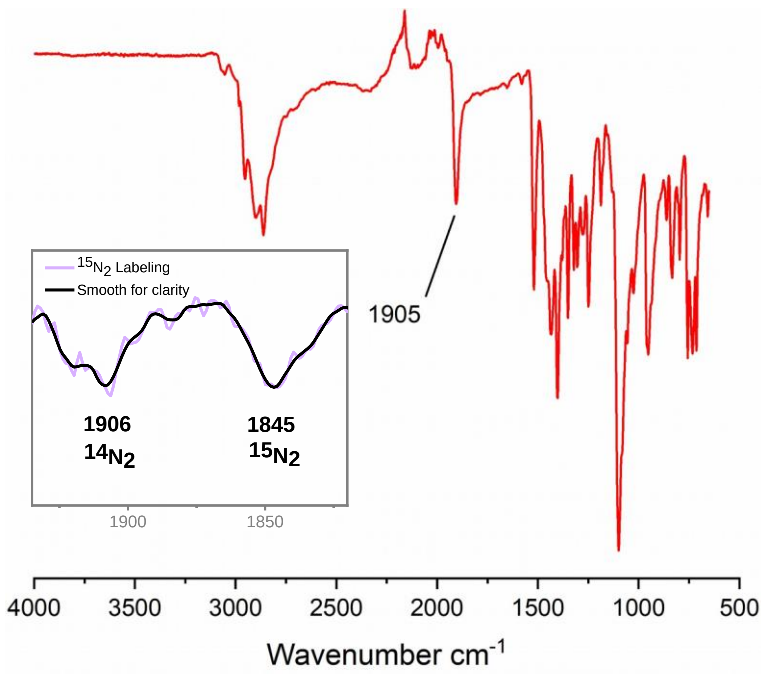

Figure S17. ATR-IR spectrum of solid **3**; the inset shows the ATR-IR spectrum of solid $\mathbf{3}^{\mathbf{15N2}}$ in the range 1820 – 1940 cm^{-1} (containing ~50% $^{15}N_2$ and $^{14}N_2$ each; purple: experimental data; black: smooth for clarity)**.**

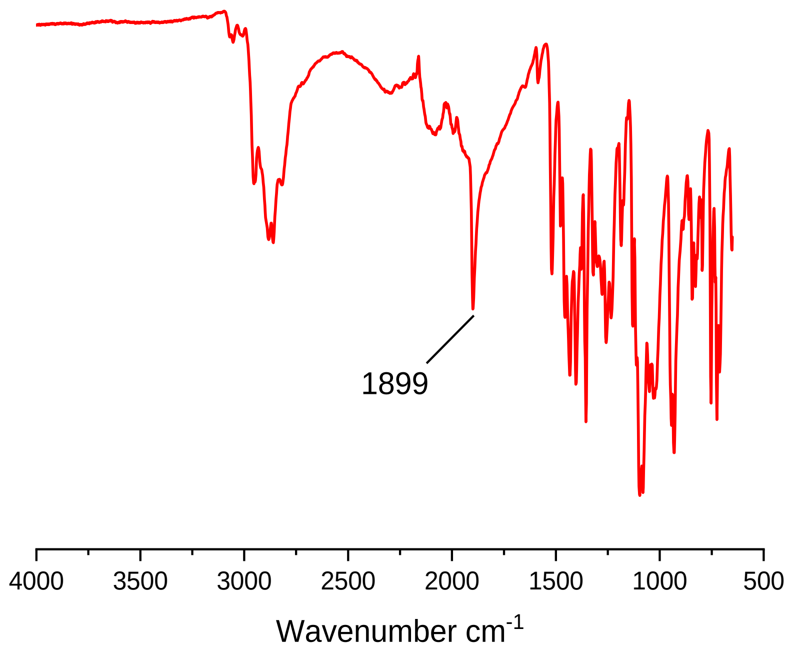

Figure S18. ATR-IR spectrum of solid **4**.

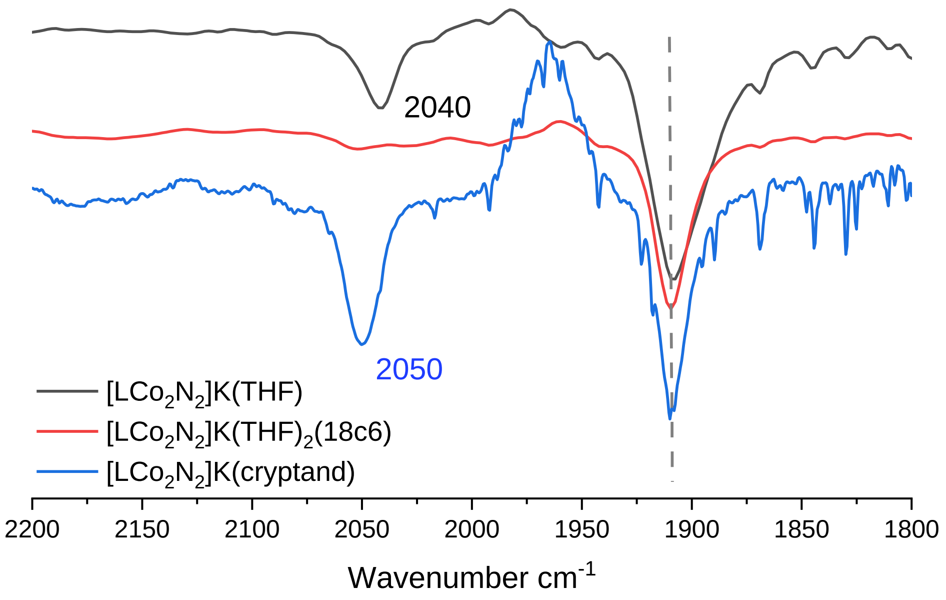

Figure S19. IR spectra (in the range 1800 – 2200 cm^{-1}) of **2**, **3** and **4** in THF at room temperature.

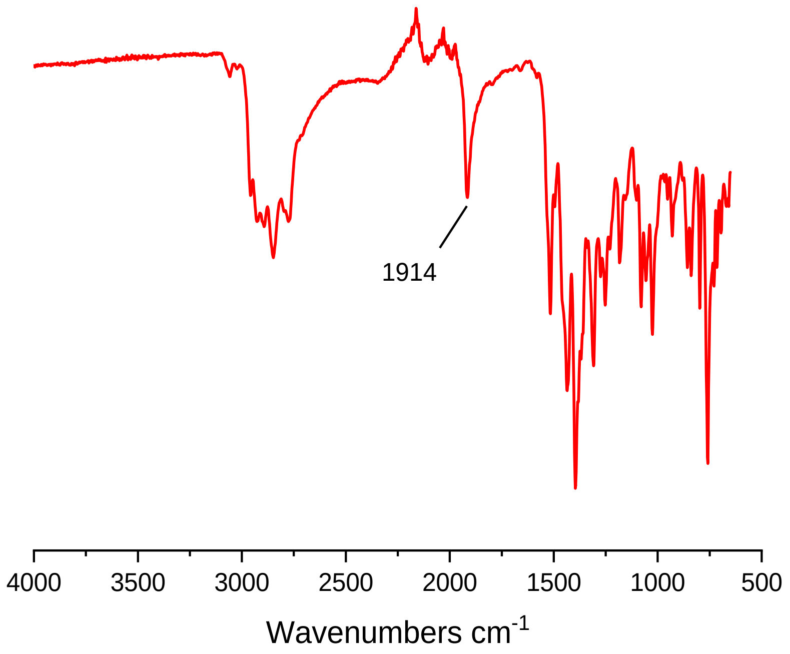

Figure S20. ATR-IR spectrum of solid **2**(THF)·$KBEt_3H$.

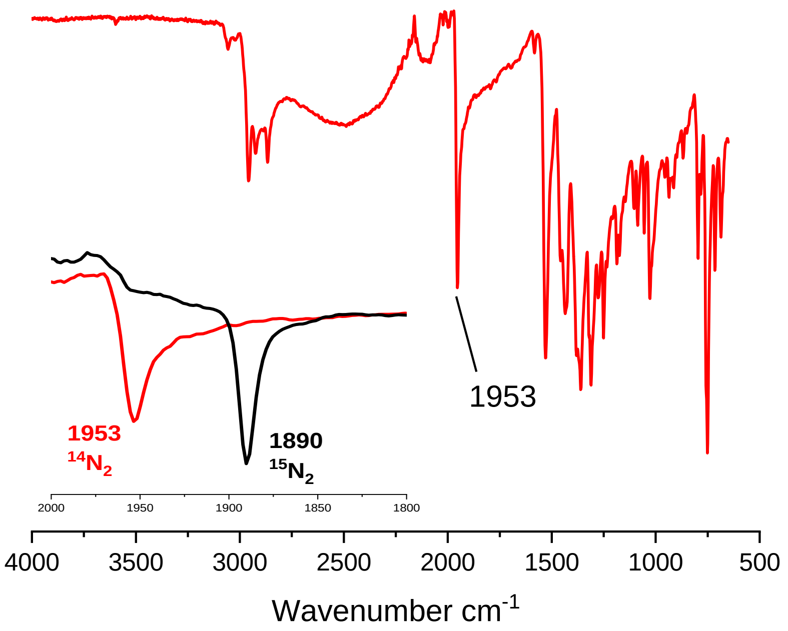

Figure S21. ATR-IR spectrum of solid **6** and **6**$^{\mathbf{15N2}}$.

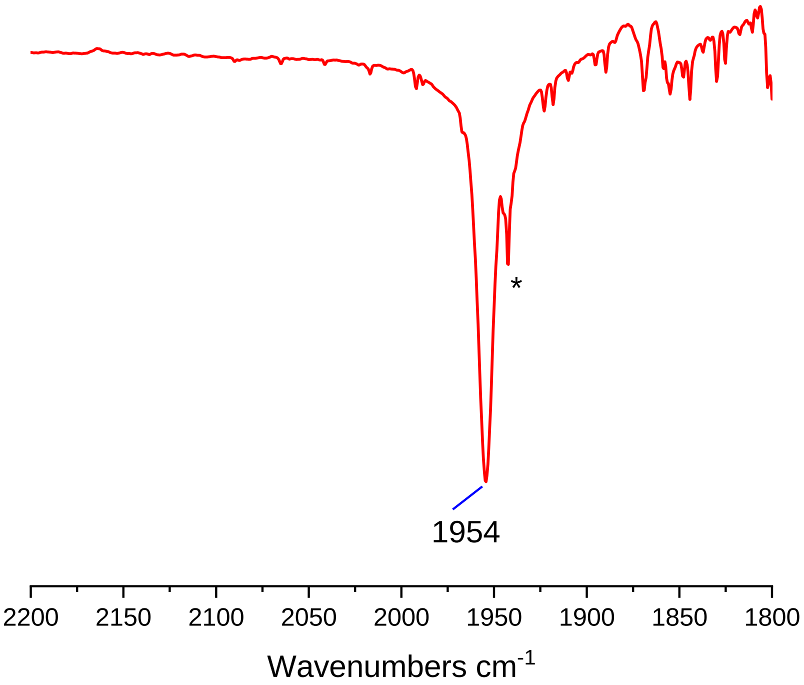

Figure S22. IR spectrum (in the range 1800 – 2200 cm^{-1}) of **6** in toluene (*: 1942 cm^{-1}, probably due to incomplete background subtraction of the solvent signals).

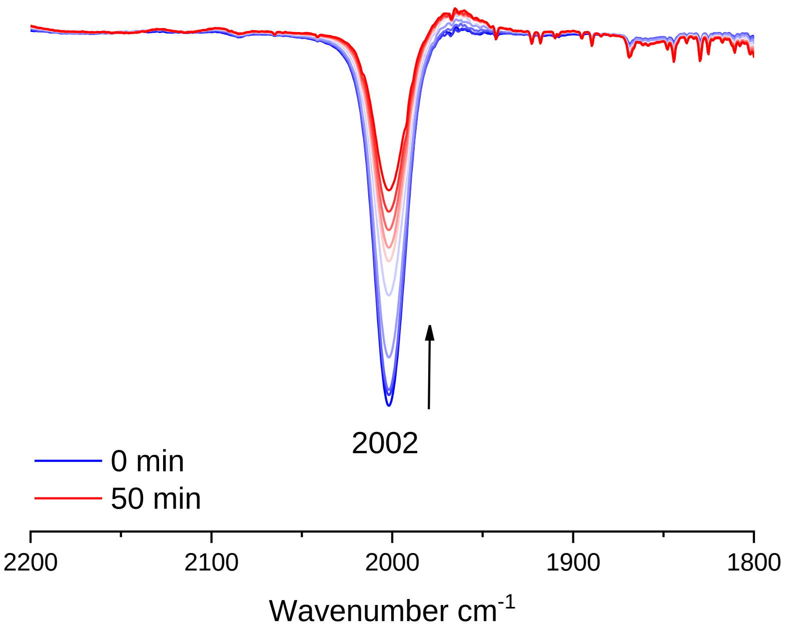

Figure S23. IR spectrum (in the range 1800 – 2200 cm^{-1}) of **6** in THF, and evolution of the spectrum over the course of 50 min.

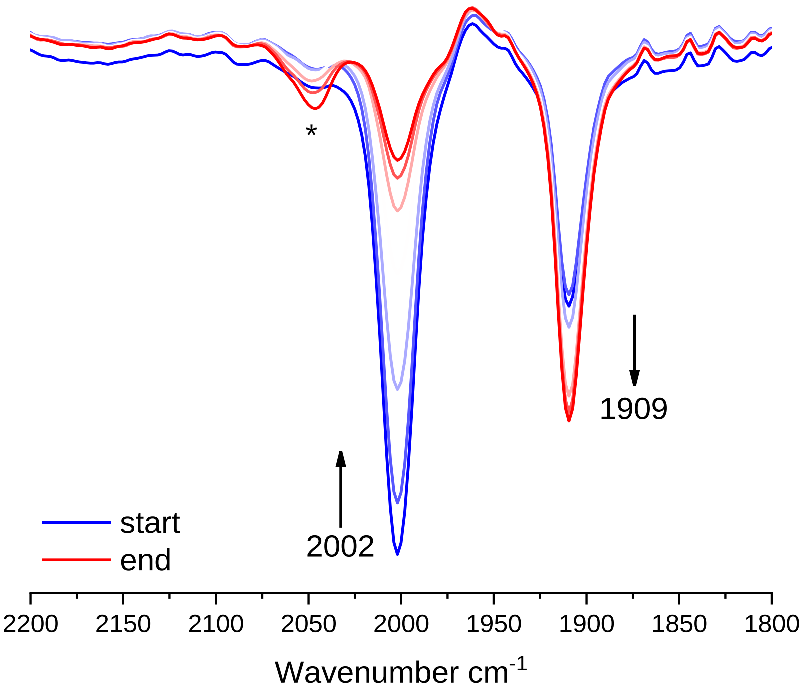

Figure S24. SEC-IR monitoring of the re-reduction of oxidized **3** in THF (3.0 mM of **3** in 0.2 M ($^{n}Bu_4N)PF_6$ solution in THF; WE: Pt mesh, CE: Pt wire and RE: Ag wire; scan rate 10 mV/s; the asterisk * indicates a new peak at 2048 cm^{-1} appearing during the re-reduction, which possibly originates from $[LCo_2(N_2)_2]^-$ as discussed in the main text).

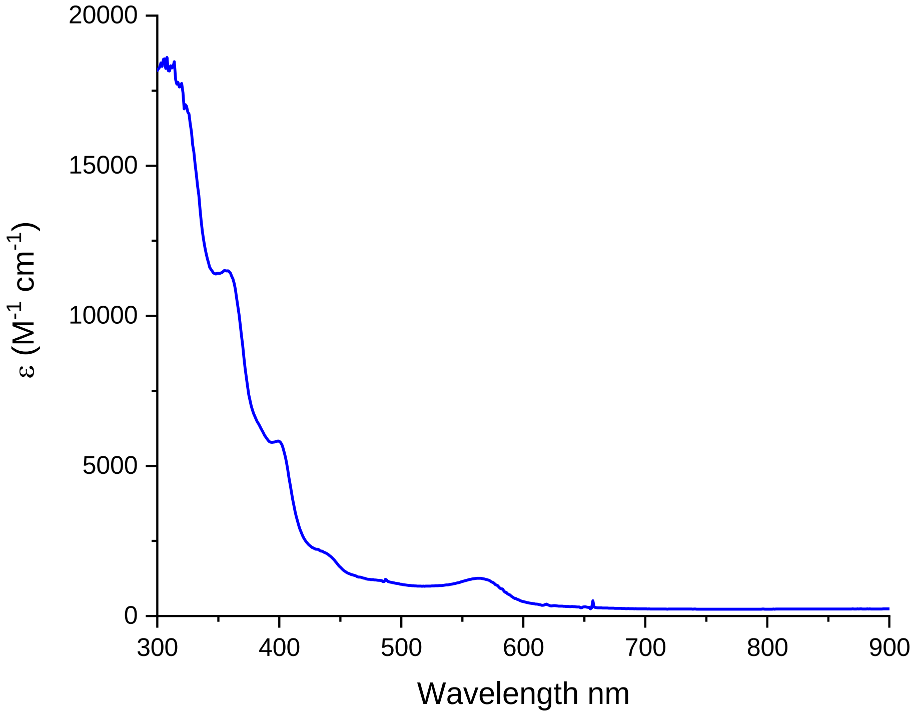

Figure S25. UV-vis spectrum of **1** in THF at room temperature.

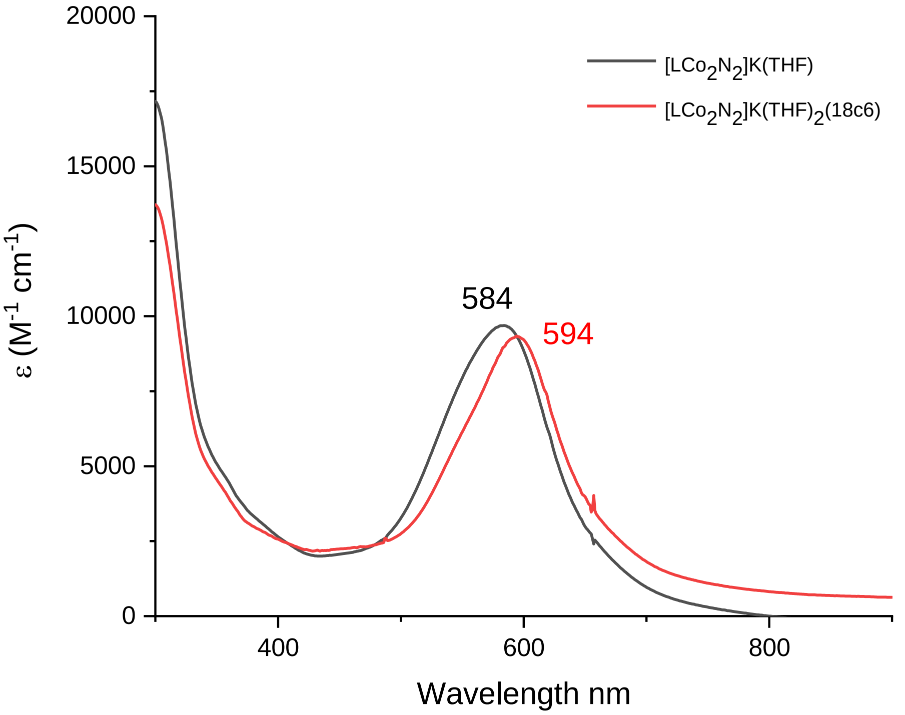

Figure S26. UV-vis spectra of **2** and **3** in THF at room temperature.

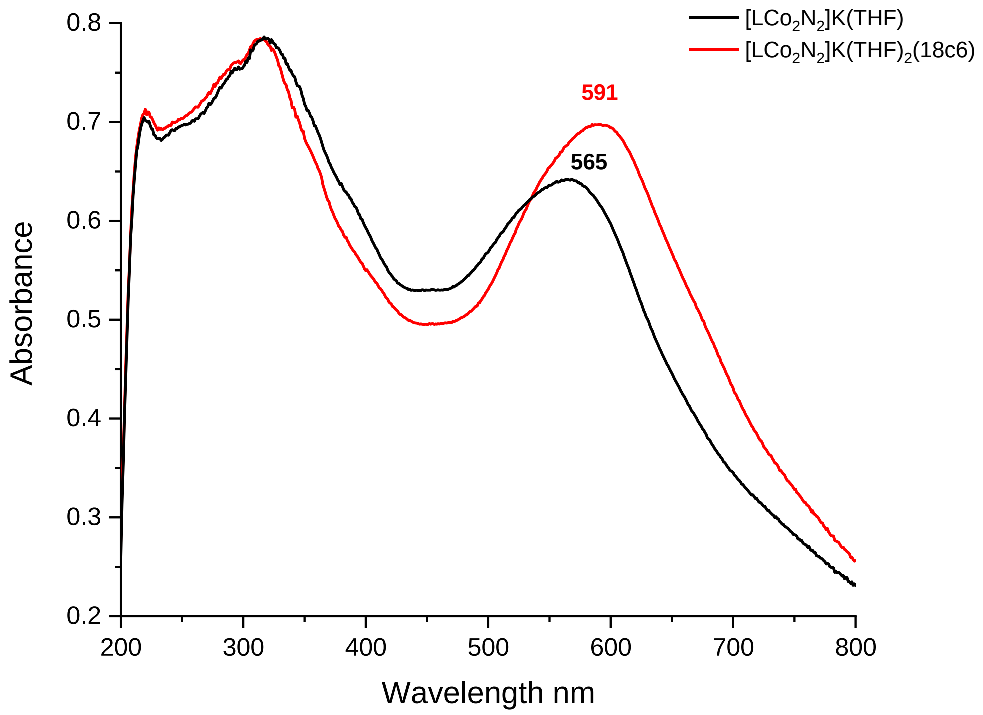

Figure S27. UV-vis spectra of solid samples of **2** and **3** at room temperature.

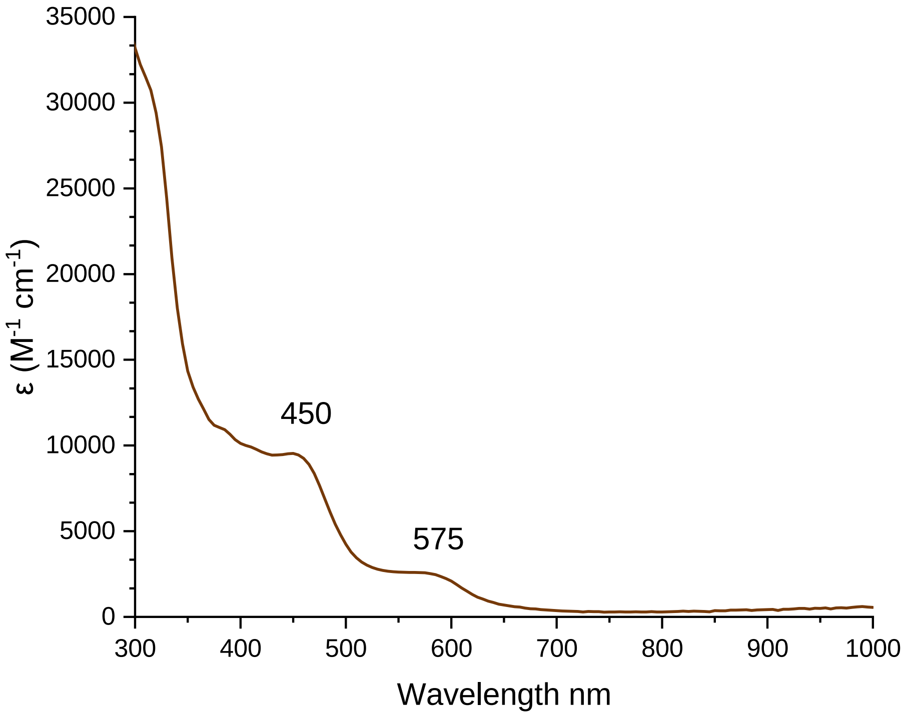

Figure S28. UV-vis spectra of **6** in toluene at room temperature.

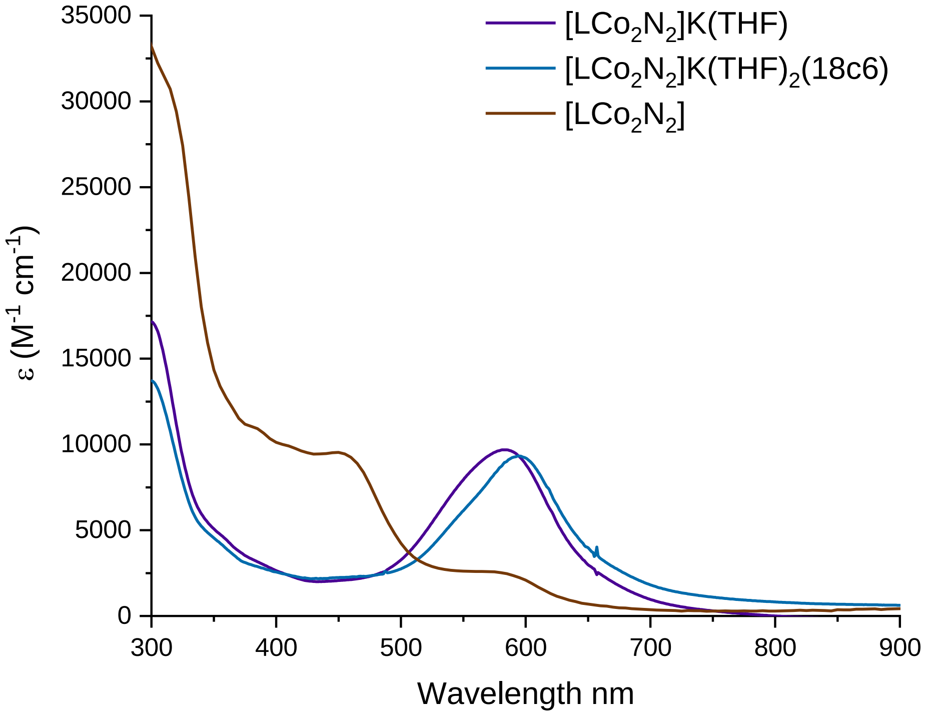

Figure S29. Comparison of UV-vis spectra of **2**, **3** in THF and **6** in toluene solution at room temperature.

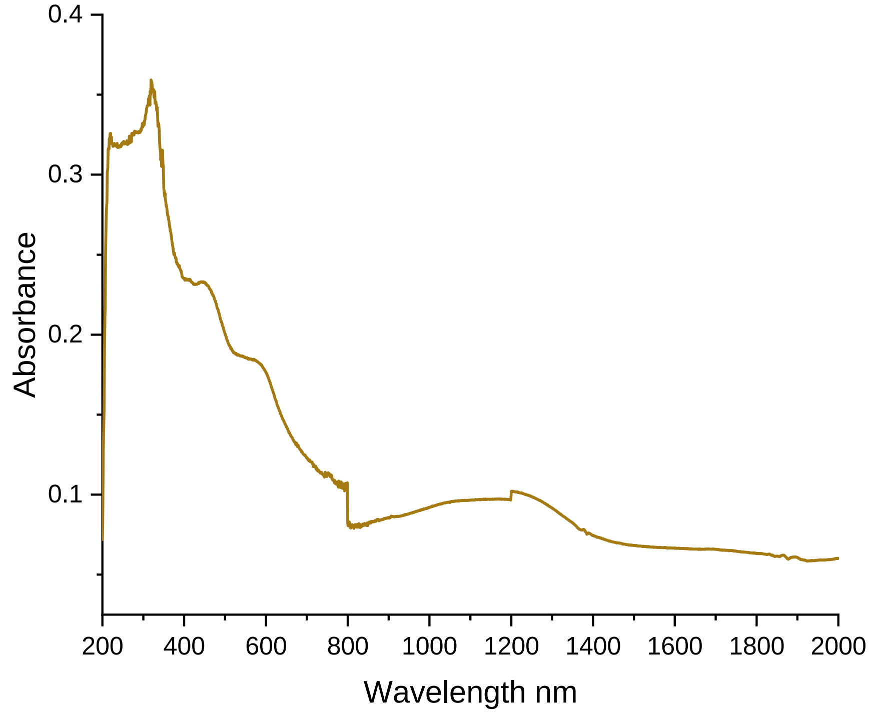

Figure S30. UV-vis-NIR spectrum of solid **6** (steps at 800 nm and 1200 nm due to the change of the light source of the spectrometer).

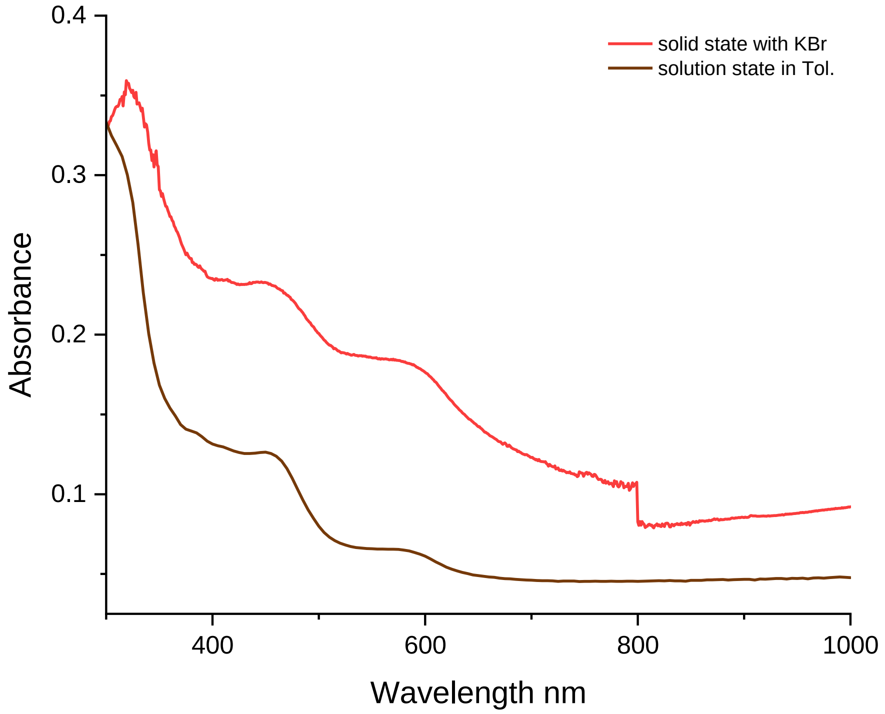

Figure S31. Comparison of the UV-vis spectra of solid **6** (KBr; red spectrum; step at 800 nm due to the change of the light source of the spectrometer) and of **6** in toluene solution (black spectrum).

5 Magnetic Measurements and EPR Spectroscopy

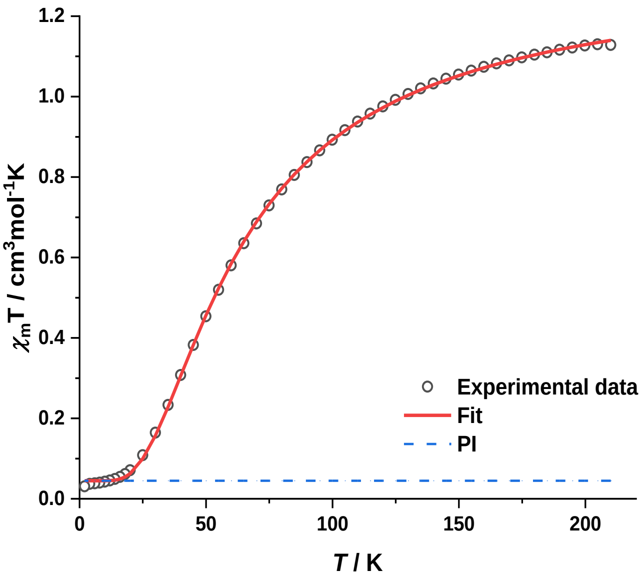

Figure S32. $\chi_M T$ vs. T plot for solid **1**; the red line represents the best fit with 2× S = ½, J = –39.2 cm^{-1} and g = 2.65. TIP = 1450·10^{-6} cm^3mol^{-1}, PI = 2.4 % (with S = 3/2).

Magnetic exchange interactions could be mediated via a benzene molecule which is arranged in a T-shaped edge-to-face fashion to the Co(β-diketiminato) 6-membered rings (Figure S33). Distance and angle are in the range reported for CH/π hydrogen bonds.[5]

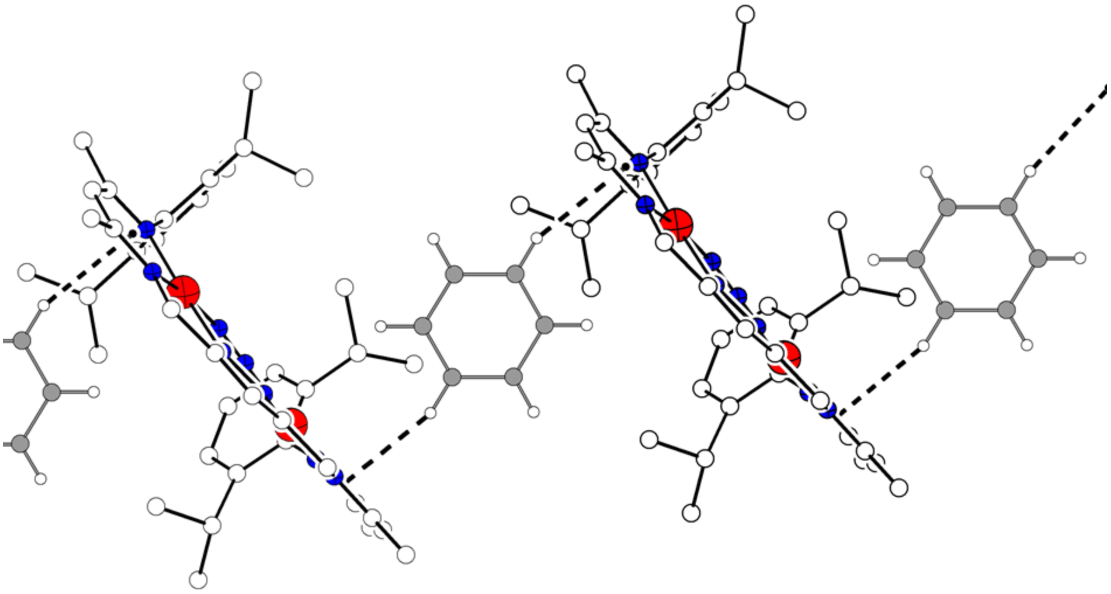

Figure S33. Emphasis of possible intermolecular CH/π hydrogen bond interactions between the Co-nacnac 6-membered rings and benzene molecules in **6**. Distance of the plane of the 6-membered ring (Co1, N2, C4, C5, C6, N3) to the closest hydrogen atom of the benzene molecule (H22) is 2.56 Å, to the carbon atom bound to the hydrogen atom 3.449(2) Å. The angle of the centroid of the plane (Co1, N2, C4, C5, C6, N3) is Cg···H–C(benzene) is 159.9(1) deg.

EPR spectroscopy

The reactivity of **6** in the presence of THF was also investigated using EPR spectroscopy. For this, an EPR sample consisting of ca. 0.4 mg **6**, 250 µL toluene and 1 µL of THF was prepared and filled into a sealed EPR tube. The solution was left at room temperature for ca. 2 minutes before shock freezing the sample in liquid nitrogen. This sample was then immediately brought into the pre-cooled X-band cw EPR resonator and measured at 12 K. The obtained spectrum is shown in Figure 34. Notably, an intense resonance at an effective *g* value of ca. 6.0 is observed, which points to an S = 3/2 spin state.[6] The ^{59}Co hyperfine coupling displayed on this resonance resembles the one observed for **6** in pure toluene, pointing to localization of the spin density on a single Co nucleus.

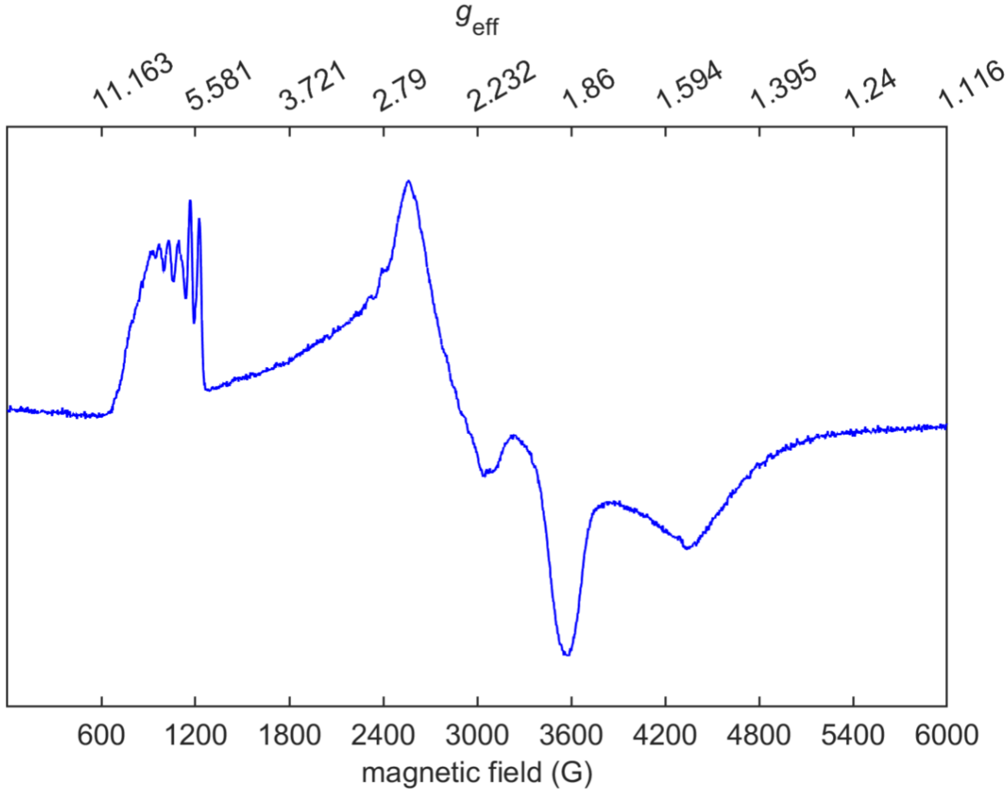

Figure S34. cw EPR spectrum of **6** in toluene/THF (250:1) measured at 9.376 GHz and 12K.

We note that more than one paramagnetic species might be responsible for the EPR spectrum displayed in Figure S34. In fact, we have observed other, transient species when freezing the sample solution at earlier times as well as emergence of new species when waiting for longer times. However, the complexity of the spectra and the sample prevents a detailed analysis.

A comparison of the spectrum obtained in presence of THF (Figure S34) with the spectrum in pure toluene (Figure 7 in the main text) is presented in Figure S35. The comparison reveals that even in pure toluene either an identical or structurally very similar S = 3/2 Co(II) species is present as a minority species.

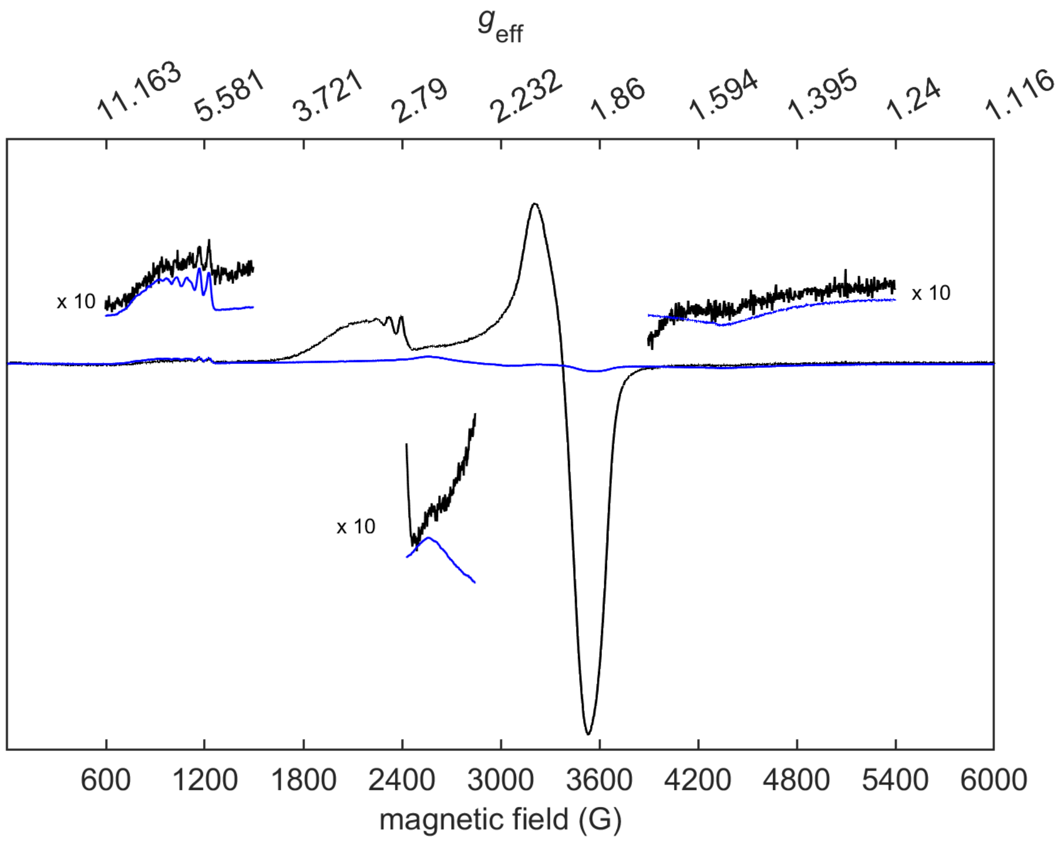

Figure S35. Comparison of the cw EPR spectrum of **6** in pure toluene (black line, same as in Figure 7 in main text) or in toluene/THF (250:1) (blue line, same as in Figure S34). The insets show magnified views of spectral features which occur in both spectra. The blue line is scaled to match the intensity of these features in the black spectrum.

6 Electrochemical Data

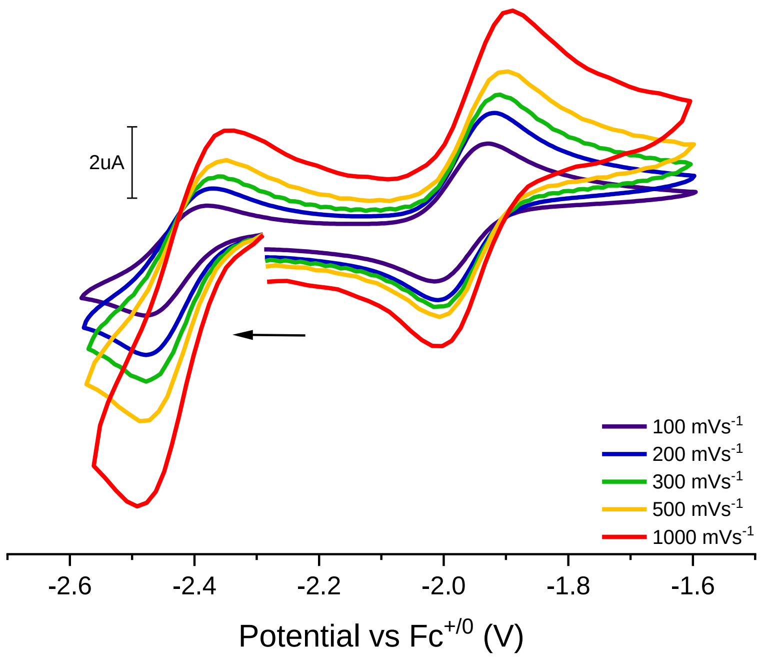

Figure S36. Cyclic voltammograms of **2** (1 mM in THF), using a glassy carbon (3 mm outer diameter) working electrode, a Pt wire counter electrode, a Pt wire reference electrode and 0.2 M [^{n}Bu$_4$N][PF$_6$] as the supporting electrolyte.

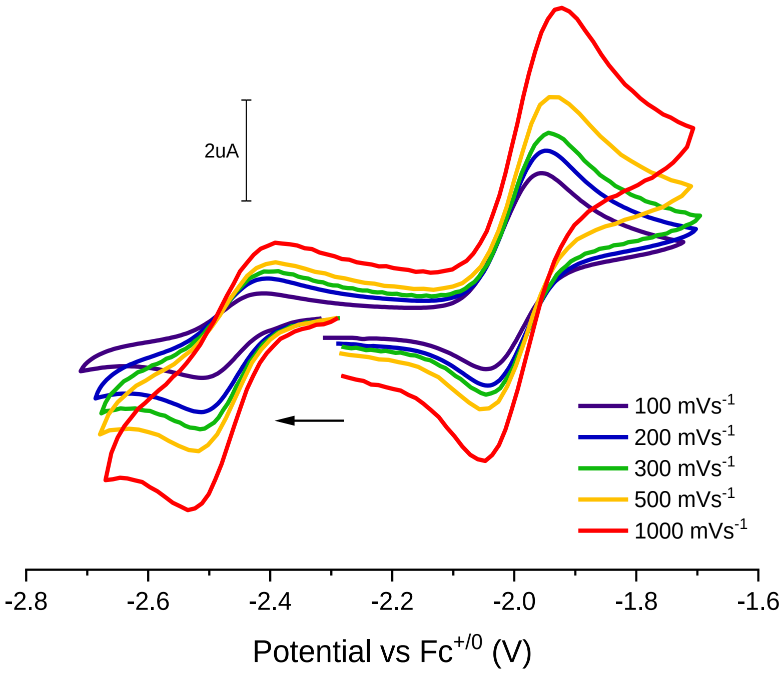

Figure S37. Cyclic voltammograms of **3** (1 mM in THF), using a glassy carbon (3 mm outer diameter) working electrode, a Pt wire counter electrode, a Pt wire reference electrode and 0.2 M [^{n}Bu$_4$N][PF$_6$] as the supporting electrolyte.

7 Computational Details and Additional Computational Data

Density Functional Theory

All quantum chemical calculations were carried out with the ORCA suite of programs, version 5.0.4.[7] Geometry optimizations started from crystallographic coordinates where available. The starting structure for the complex with two end-on bound dinitrogen was created from a previously optimized geometry by adding a second N_2 unit. All structures were fully relaxed using UKS-DFT with the BP86 density functional and the ZORA scalar-relativistic Hamiltonian.[8] To accelerate the calculations, the resolution of identity approximation as employed.[9] All atoms are described with the scalar-relativistically recontracted ZORA-def2-TZVP basis set; the SARC/J auxiliary basis was used.[10] Grimme's dispersion correction D3BJ with Becke–Johnson damping was applied.[11] A conductor-like polarizable continuum model (CPCM) was used as implicit solvation model with the solvent parameters of THF.[12] Frequency calculations were performed with the optimized geometries and the same settings as above.

All attempts to converge a broken-symmetry electronic structure approximating an open-shell singlet electronic structure converged to the closed-shell singlet electronic structure.

We note that using DFT calculations, the fully relaxed bare complex $[LCo_2(N_2)]^-$ with a closed shell singlet electronic structure has an unscaled N–N stretching mode at 1976 cm^{-1}, an overestimation within the expected accuracy of DFT that in this case is at least partially attributed to the complex electronic nature of the ground state. Inclusion of the K^+ and two THF molecules results in an optimized structure with a K^+•2THF fragment situated above the six-membered ($\mu_{1,2}$-N_2)-Co_2-N_2^{pz} ring and an N–N stretching mode at 1949 cm^{-1}. We furthermore optimized a supermolecular unit comprised of two anion moieties with K^+•THF between them, though a frequency calculation to confirm a true minimum was not completed. There is no notable difference in N–N bond length (bare: 1.151 Å vs. K^+•2THF adduct: 1.156 Å vs. K^+•THF-crystal structure: 1.157 Å) or Mayer bond order (bare: 2.3 vs. K^+•2THF adduct: 2.1 vs. K^+•THF-crystal structure: 2.25).

Time-Dependent Density Functional Theory

Using the optimized structures or the crystal structure with relaxed hydrogen atom positions, time-dependent density functional theory calculations with the Tamm–Dancoff-approximation were performed to obtain the UV-vis absorption spectra for $[LCo_2(N_2)]^-$ and $[LCo_2(N_2)]$.[13] These calculations employed the PBE0 hybrid density functional. Relativistic effects were taken into account by the scalar-relativistically zeroth-order regular approximation ZORA and using recontracted ZORA-def2-TZVP basis sets along with the SARC/J auxiliary basis set. To speed up the calculations, the RIJCOSX approximation was applied. A conductor-like polarizable continuum model (CPCM) was used as an implicit solvation model with the solvent parameters of THF. 100 roots were calculated and the dimension of the expansion space in the Davidson procedure was chosen as five times as large. To obtain line spectra, the individual transitions were artificially broadened with Gaussian functions with a FWHM of 1000 cm^{-1} using the *orca_mapspc* utility program.

Details on the failure of TD-DFT to reproduce the experimental absorption spectrum are given below.

Coupled Cluster

For the DLPNO-CCSD(T1) calculations, very tight energy convergence criteria were used. Scalar relativistic effects were considered with the zeroth-order regular approximation (ZORA) of the def2 basis sets. Basis set combinations were employed. The basis set is used for Co, ZORA-def2-TZVP on O, C, and N, and ZORA-def2-SVP on H. The corresponding auxiliary basis sets def2-TZVPP/C on Co, def2-TZVP/C on O, C, and N, and def2-SVP/C on H were used. Normal PNO settings were used for calculations with only changing TCutPNO = $1*10^{-6}$.

CASSCF/NEVPT2

State-average complete active space self-consistent field (SA-CASSCF) calculations including spin-orbit coupling were performed considering singlet and triplet multiplicities. To incorporate correlation effects, *N*-electron valence second-order perturbation theory (NEVPT2) was employed.[14] The crystal structure coordinates were taken with optimized hydrogen atom positions. The initial active space orbitals were taken from DFT calculations as quasi-restricted orbitals (QROs). Scalar relativistic effects were considered with the zeroth-order regular approximation (ZORA) and the ZORA-def2-TZVP basis set was used for all atoms.[8] To speed up the calculations, the RIJCOSX approximation was implemented along with an auxillary basis (AutoAux).[9] The implicit solvation model CPCM was used with THF as the modeled solvent.[12] The active space contained 20 electrons in 12 orbitals (CASSCF(20,12)) and 50 singlet and triplet states were averaged with equal weights. To obtain line spectra, the individual transitions were artificially broadened with Gaussian functions with a FWHM of 1000 cm^{-1} using the *orca_mapspc* utility program.

To test the stability of the resulting electronic structure description, SA-CASSCF/NEVPT2 calculation were also performed for the same (20,12) active space with a lower number of roots of 60, i.e. 30 singlet and triplet states that were averaged with equal weights, see Fig S47, S48. Any further decrease of the number of roots as well as state specific calculations did not result in a meaningful active space or converge at all.

Failure of TD-DFT for the Prediction of Absorption Spectrum

All attempts to predict the UV-vis absorption spectrum for the crystal structure (relaxed hydrogen atom positions) and the fully optimized structure of the anion $[LCo_2N_2]^-$ with a closed-shell singlet state with and without spin-orbit coupling failed since the characteristic band at 600 nm was not reproduced. With the BP86 and PBE GGA functionals, the maxima shift slightly from ~2.6 eV to ~2.5 eV but they still fail to reproduce the characteristic band at ~2.0 eV.
While there is no absorption band in the region of 500-700 nm in the singlet state, spectral predictions based on the triplet electronic structure capture excitations in that region in both, the crystal structure and the fully optimized structure.

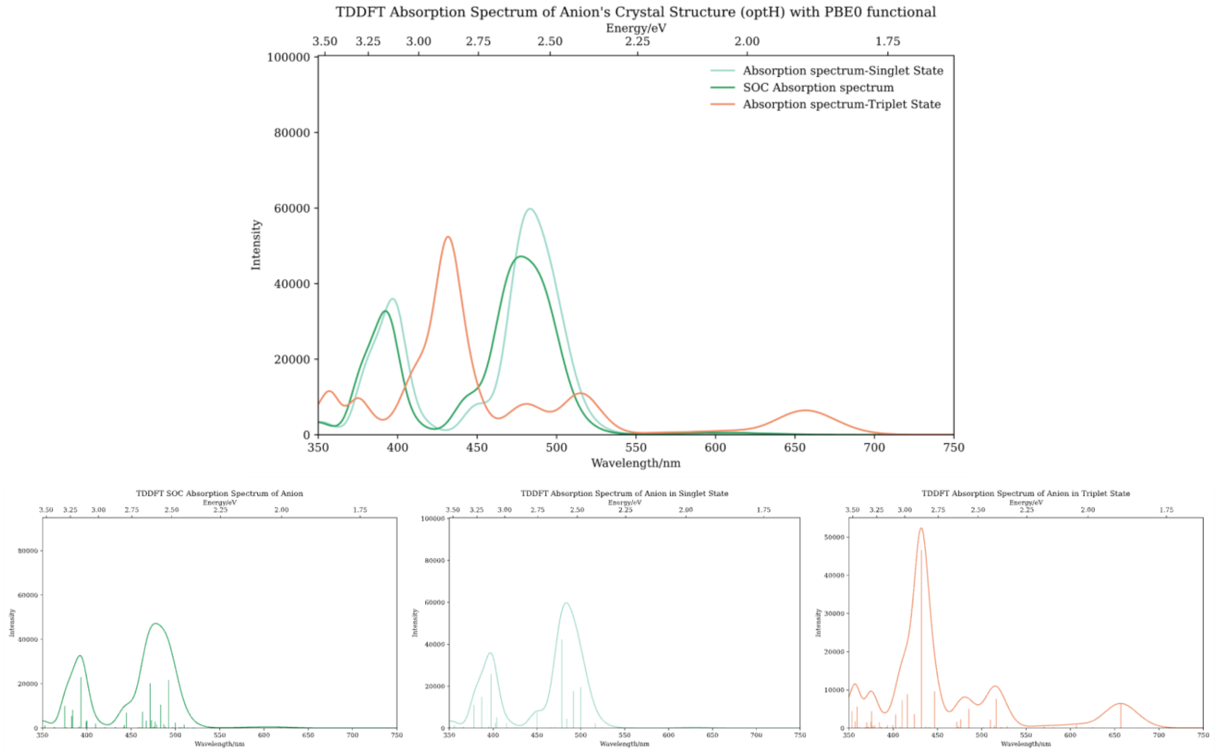

Figure S38. Top: Overlaid TD-DFT spectra of the singlet electronic structure (with and without spin orbit coupling, dark and light green traces, respectively) and triplet multiplicity (orange trace) of the crystal structure of $[LCo_2N_2]^-$ (hydrogen atoms relaxed) for the PBE0 functional. Bottom: Vertical transitions of the individual spectra in the top panel and the line spectra resulting from Gaussian broadening.

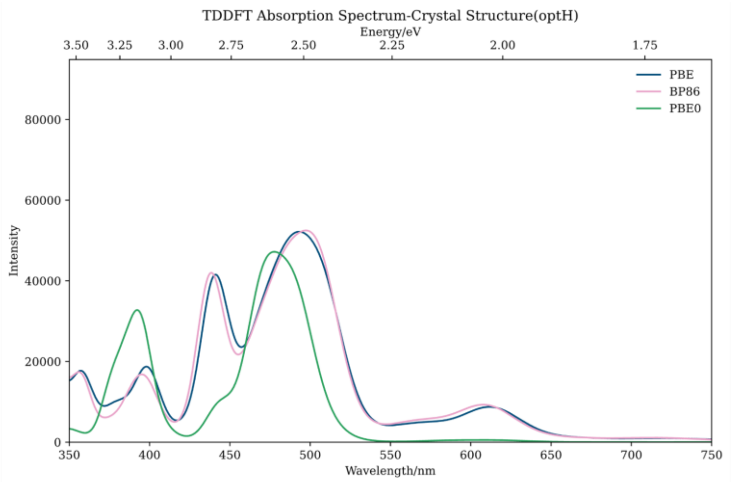

Figure S39. Overlaid TD-DFT spectra obtained from of the singlet electronic structure of the crystal structure of $[LCo_2N_2]^-$ (only hydrogen atom positions optimized) with the BP86, PBE and PBE0 functionals.

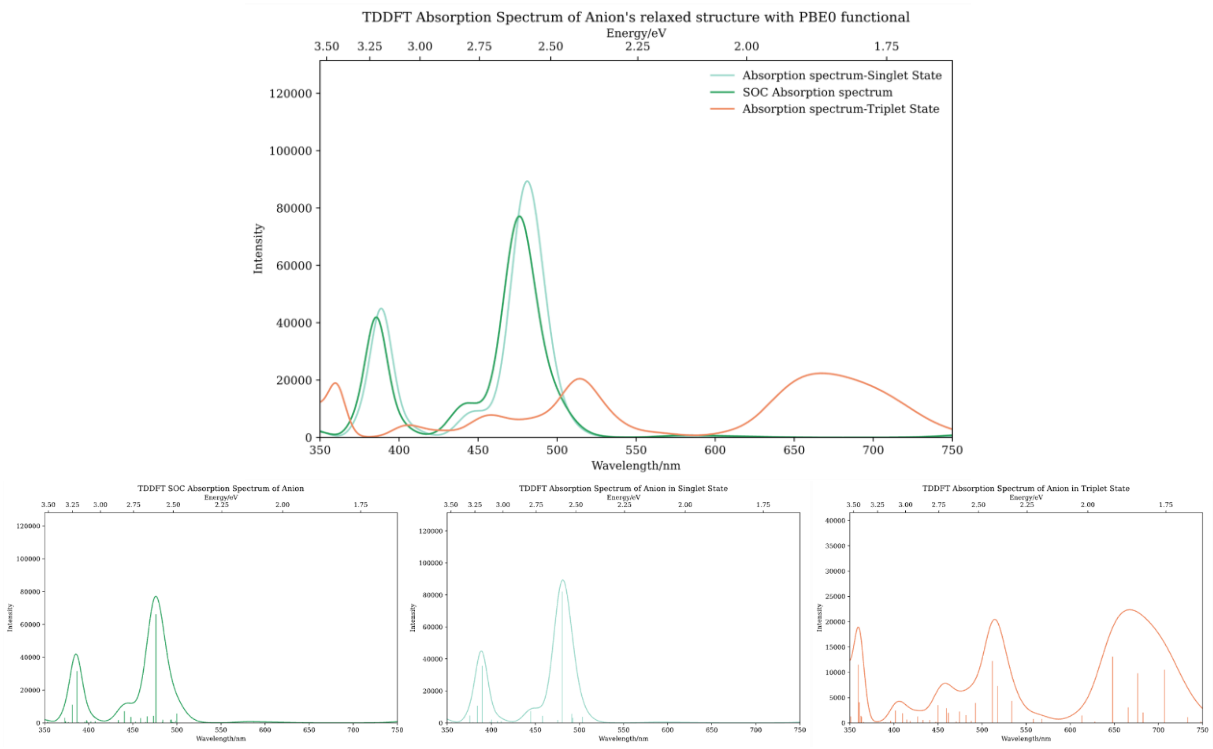

Figure S40. Top: Overlaid TD-DFT spectra of the singlet electronic structure (with and without spin orbit coupling, dark and light green traces, respectively) and triplet multiplicity (orange trace) of the crystal structure of $[LCo_2N_2]^-$ (relaxed structure) for the PBE0 functional. Bottom: Vertical transitions of the individual spectra in the top panel and the line spectra resulting from Gaussian broadening.

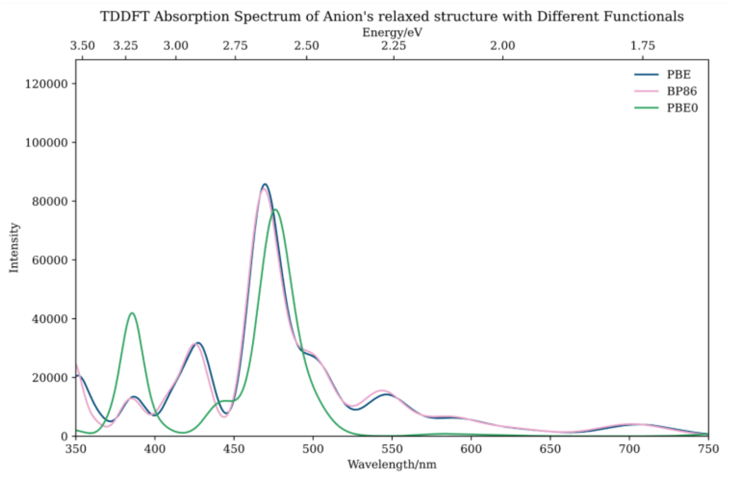

Figure S41. Overlaid TD-DFT spectra obtained from the singlet electronic structure of the relaxed structure of $[LCo_2N_2]^-$ (only hydrogen atom positions optimized) with the BP86, PBE and PBE0 functionals.

Difference Density Analysis

Transitions in the singlet state at ~2.6 eV

Crystal Structure	Relaxed Structure

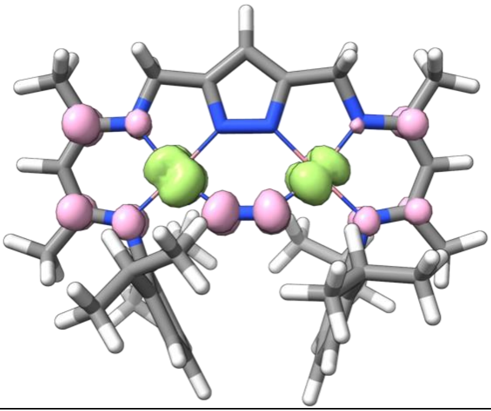
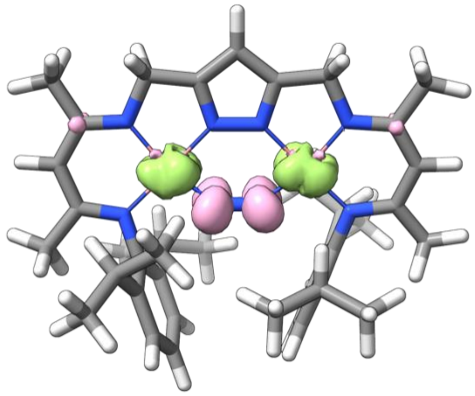

Transitions in the triplet state at ~1.9 eV

Crystal Structure	Relaxed Structure

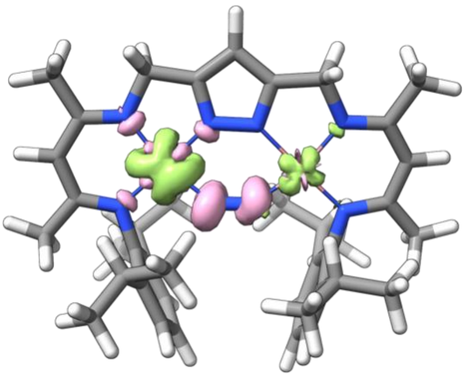
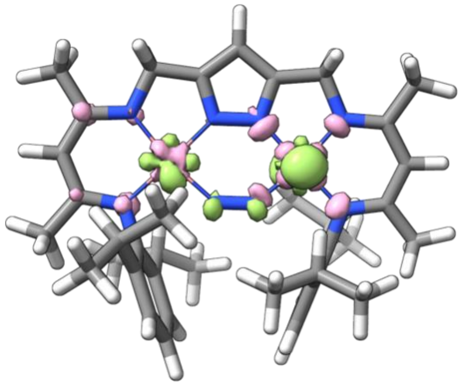

Figure S42. Difference density plots (iso value = 0.0025 a.u.; pink = negative; green = positive) from calculations with the PBE0 functional and ZORA-def2-TZVP basis set.

TD-DFT Oscillator Strength with BP86 functional and ZORA-def2-TZVP basis set

State	Wavelength (nm)	f_{osc}
13	614.6	0.024
33	509.4	0.212

State 13	State 33

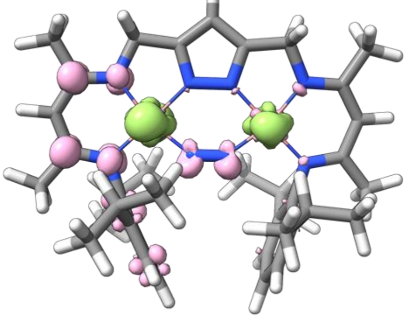
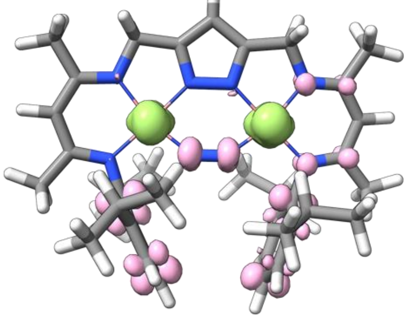

Figure S43. Difference density plots (iso value = 0.0025 a.u.; pink = negative; green = positive) from calculations with the BP86 functional and ZORA-def2-TZVP basis set.

Fractional Occupation Number Weighted Electron Density (FOD) Analysis

The FOD analysis yields significant and delocalized values for *N*(FOD) and *ρ*(FOD), see Figure S44, which fall into the category of a multi-reference (MR) case.

The value of *N*(FOD) is 2.67 and 2.04 for the PEB0 (*T* = 10000 K) and BP86 (*T* = 5000 K) functionals, respectively. Notorious MR cases, such as the diatomic C_2 molecule (*N*(FOD)=1.74) or the TS for insertion of Be into the H_2 bond (*N*(FOD)=1.91), have smaller values though with a smaller total number of electrons.[18] The degree of delocalization appears similar to that of the transition state for isomerization of a complex with a $Cu_2(\mu\text{-}O)_2$ core (*N*(FOD) = 0.829).[18]

PBE0 (T = 10000 K)	BP86 (T = 5000 K)

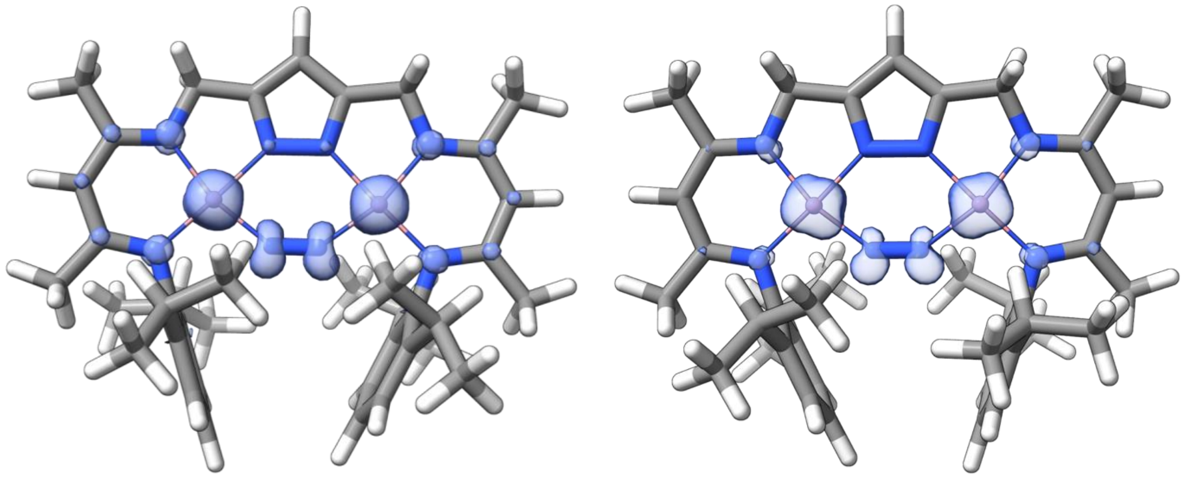

Figure S44. The FOD analysis plotted with the contour value of 0.005 e/Bohr^3 using the def2-TZVP basis set shows significant delocalization at the cobalt and nitrogen center along with some delocalization to the ligand.

Results of the coupled cluster calculations

The coupled cluster calculations are performed on the crystal structure geometry of **2** with relaxed hydrogen positions to avoid any artefacts due to geometry relaxation.

Table S1. Energies from the DLPNO-CCSD(T1) calculations of complex $[LCo_2(N_2)]^-$ with TCutPNO threshold of 1*10^{-6}; all energies are given in Hartree units except the relative energies which are given in kcal/mol. The basis sets are as follows, Co: def2-TZVPP; C, N: def2-TZVP; H: def2-SVP.

Method	E-ref	E-corr(CCSD)	E-corr(T1)	E-CCSD(T1)	T1	E-rel(T1)
**HF-CC singlet**	-4706.48994441	-9.65936496	-0.46116817	-4716.61047754	0.0162	0.00
**HF-CC-triplet**	-4706.36568781	-9.72615424	-0.47078106	-4716.56262311	0.0217	30.0
**KS-CC-singlet**	-4705.69854482	-10.38572480	-0.61467681	-4716.69894644	0.0195	0.00
**KS-CC-triplet**	-4705.61067999	-10.43500737	-0.62598747	-4716.67167484	0.0212	17.1

Abbreviations:
HF: Hartree–Fock reference determinants.
KS: Kohn–Sham reference determinants.
E-ref: Electronic energy of the reference wave function.
E-corr(CCSD): Correlation energy from the coupled cluster with singles and doubles excitation.
E-corr(T1): Perturbative triples excitation correlation energy.
E-CCSD(T1): Total energy with perturbative triples.

Table S2. Doubles Amplitudes of DLPNO-CCSD(T1) calculations of complex $[LCo_2(N_2)]^-$ with TCutPNO threshold of $1*10^{-6}$.

HF-CC singlet			*HF-CC triplet*		
83 > 199	83 > 199	0.079701	198a > 200a	80b > 198b	0.061815
84 > 199	84 > 199	0.064122	198a > 200a	79b > 198b	0.058705
82 > 199	82 > 199	0.062945	73a > 201a	73b > 201b	0.057941
76 > 200	76 > 200	0.058722	111a > 202a	111b > 202b	0.052064
112 > 201	112 > 201	0.051642	198a > 200a	78b > 198b	0.049961
145 > 200	145 > 200	0.049749	198a > 200a	81b > 198b	0.048740
92 > 200	92 > 200	0.049400			
140 > 199	140 > 199	0.048733			
129 > 199	129 > 199	0.048271			
182 > 199	182 > 199	0.048158			
164 > 201	164 > 201	0.047331			
146 > 201	146 > 201	0.047238			
143 > 199	143 > 199	0.046961			
136 > 199	136 > 199	0.046845			
KS-CC singlet			*KS-CC triplet*		
84 > 199	84 > 199	0.070785	199a > 201a	195b > 198b	0.109537
83 > 199	83 > 199	0.057005	198a > 200a	195b > 198b	0.062549
82 > 199	82 > 199	0.055242	82a > 200a	82b > 200b	0.051736
139 > 199	139 > 199	0.047784			
198 > 200	198 > 200	0.047205			
145 > 201	145 > 201	0.046557			
142 > 199	142 > 199	0.046423			
135 > 199	135 > 199	0.046345			
75 > 200	75 > 200	0.046214			
181 > 199	181 > 199	0.046210			
129 > 199	129 > 199	0.045828			
191 > 199	191 > 199	0.045787			

CASSCF/NEVPT2 results

The CASSCF/NEVPT2 calculations are performed on the crystal structure geometry of **2** with relaxed hydrogen positions to avoid any artefacts due to geometry relaxation. The active space consists of bonding and antibonding molecular orbitals of the dinitrogen unit and cobalt ions (see main text figure and Figure S45), containing 20 electrons in 12 orbitals, or in short (20,12), averaged over 50 singlet and 50 triplet roots to cover a sufficient number of states to represent the relevant region in the UV-vis spectrum.

In the CASSCF/NEVPT2 calculation, the ground state (triplet root 2, 0.000 eV) is energetically close to the next three excited states (singlet root 0, 1144.6 cm^{-1}; singlet root 2, 1184.4 cm^{-1}; singlet root 6, 1267.0 cm^{-1}). Upon inclusion of spin-orbit coupling, the ground state is composed of triplet root 2 (81% weight, m_s ±1 components) and singlet root 0 (16% weight), see SI for details. The first excited state above the spin-orbit coupled ground state is found at 102.2 cm^{-1}, with the second excited state following at 103.5 cm^{-1}. Both are dominated by triplet root 2 with different contributions of the respective CSFs. These three close-lying states are separated from the next higher states by more than 1000 cm^{-1}. This demonstrates that only the ground state is strongly affected by spin-orbit coupling in terms of its energetic stabilization, while the higher lying states remain at similar energies.

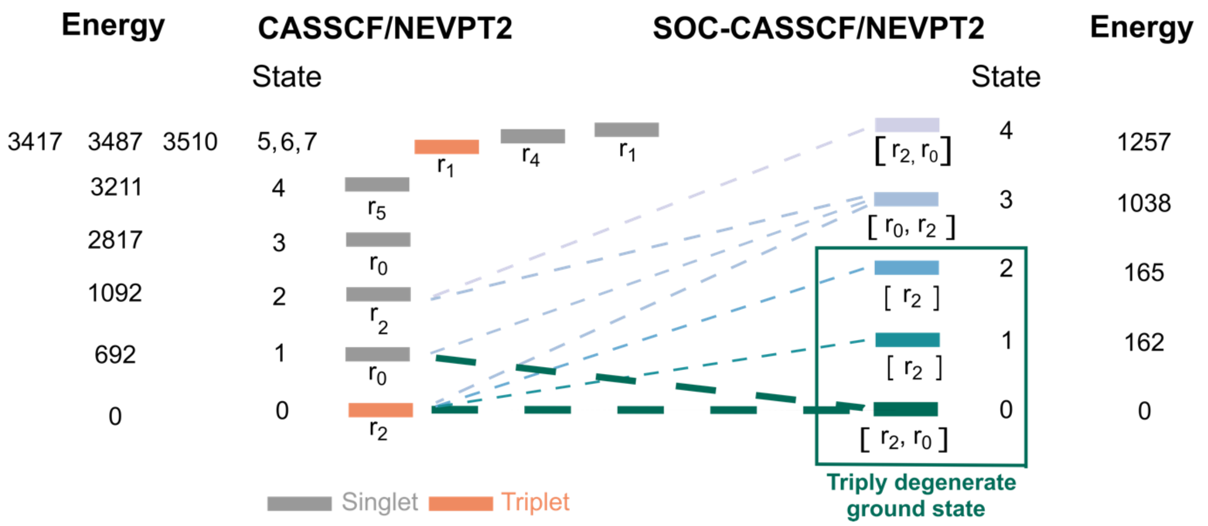

Figure S45: Electronic structure description of state averaged CASSCF/NEVPT2 calculation with and without spin-orbit coupling. Energy is in cm^{-1}

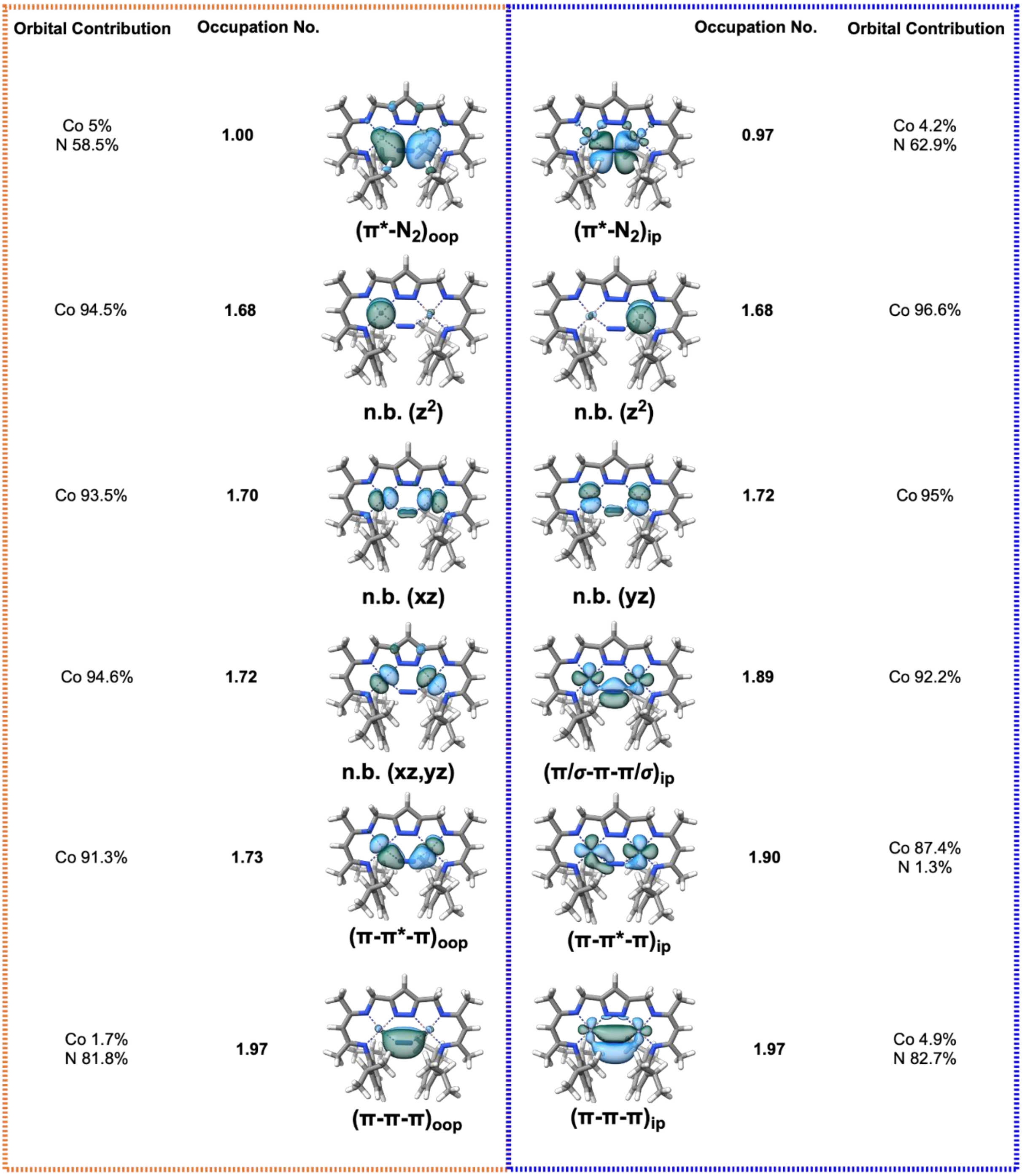

Figure S46. Molecular orbitals of the (20,12) active space for $[LCo_2(N_2)]^-$. Images are created in Chimera with an isosurface value of 0.025. Orbital contribution is taken from Löwdin reduced orbital populations.

Absorption Spectrum at the CASSCF and CASSF/NEVPT2 levels without SOC

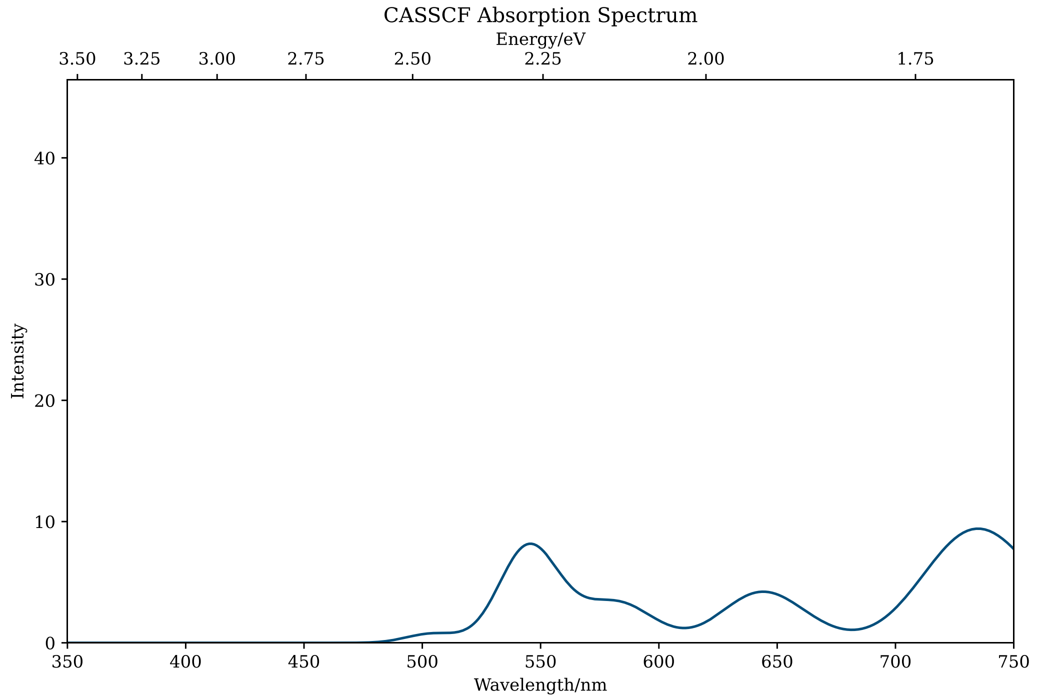

Figure S47. Absorption spectrum at the CASSCF level of the (20,12) active space for $[LCo_2(N_2)]^-$. 50 singlet and triplet states were averaged with equal weights.

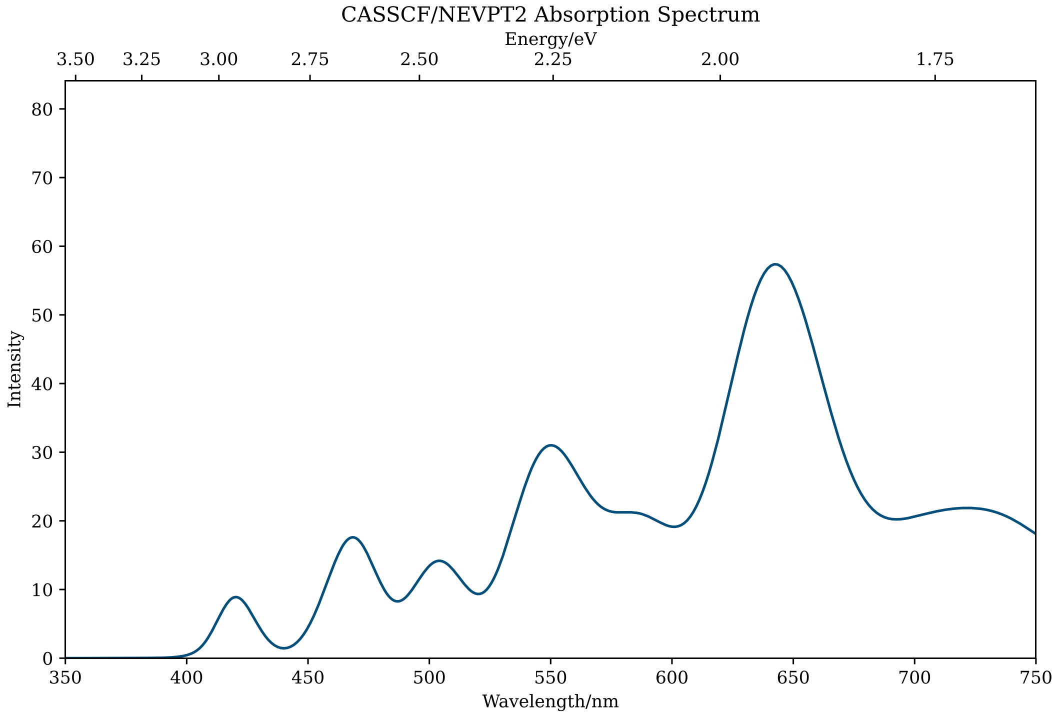

Figure S48. Absorption spectrum at the CASSCF/NEVPT2 level of the (20,12) active space for $[LCo_2(N_2)]^-$. 50 singlet and triplet states were averaged with equal weights.

Prediction of the UV-vis spectrum with SOC-CASSCF/NEVPT2

The overstabilization of the triplet state means that a prediction of the UV-vis spectrum with this electronic structure description must remain an approximation. Allowing excitations from the three degenerate states leads to a broad absorption feature around 590 nm, see Figure S49. In the higher energy region < 500 nm, the discrepancy in intensity with respect to experiment originates from the number of calculated excitations. If one assumes that calculations with a larger active space would resolve the overstabilization of the triplet state at the CASSCF level, it can be expected that the intense absorption band at 590 nm is due to excitations from a predominantly singlet ground state to a state with significant triplet contributions, all of which may have some degree of multiconfigurational character and be subject to spin-orbit coupling.

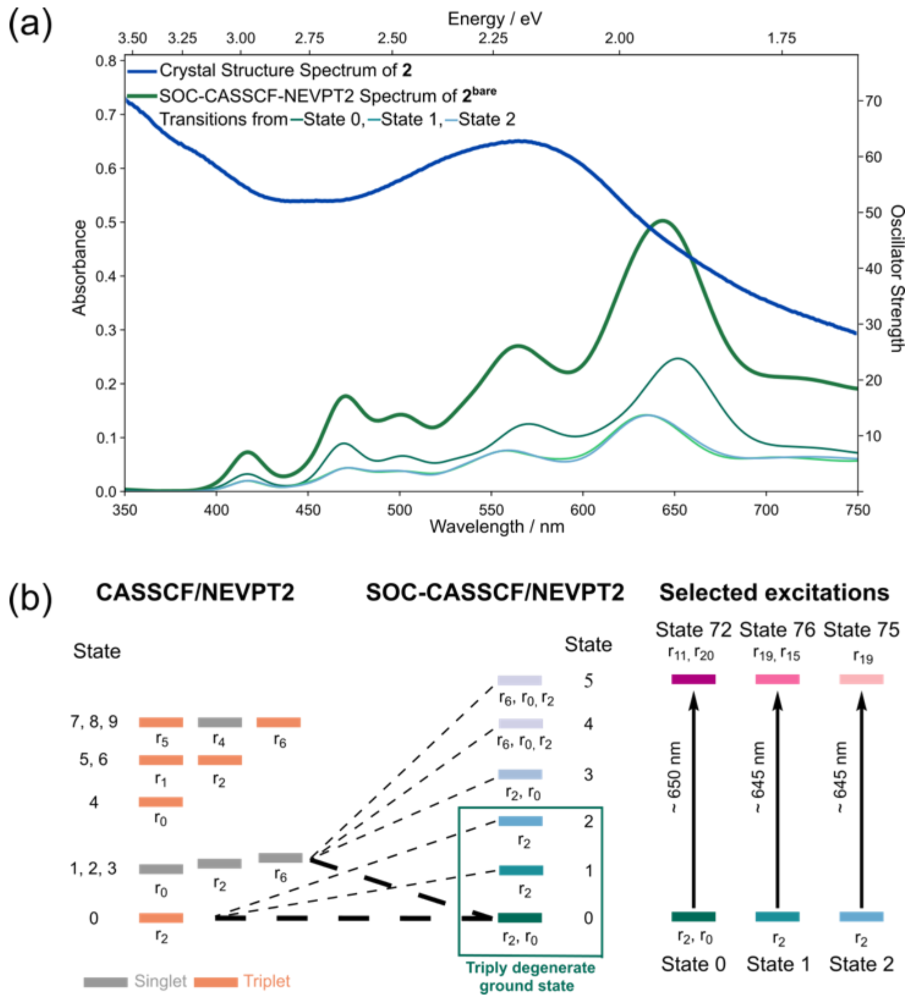

Figure S49. a) UV-vis absorption spectrum of solid [$LCo_2(N_2)$][K(THF)] and CASSCF/NEVPT2 (12,20) absorption spectrum (broader linewidth) along with sub-absorption spectra of degenerate ground state with first and second spin-orbit coupled excited state. b) Electronic structure description of state averaged CASSCF/NEVPT2 calculation after applying spin-orbit coupling along with the nature of selected excitations of the maximum absorption observed in the simulated UV-Vis spectrum.

Table S3. SA-CASSCF transition energies for the lowest root (root 0, multiplicity 3, -4741.132395759 Eh) for [$LCo_2(N_2)$]$^-$.

State	Root	Multiplicity	Δ*E*/eV	Δ*E*/cm^{-1}
0	0	3	0.000	0.0
1	1	3	0.018	144.2
2	0	1	0.039	312.7
3	1	1	0.115	928.5
4	2	3	0.155	1109.2
5	2	1	0.265	1249.0

Table S4. CASSCF/NEVPT2 corrected transition energies for the lowest root (root 2, multiplicity 3, -4750.347103483 Eh) of $[LCo_2(N_2)]^-$.

State	Root	Multiplicity	Δ*E*/eV	Δ*E*/cm^{-1}
0	2	3	0.000	0.0
1	0	1	0.142	1144.6
2	2	1	0.147	1184.4
3	6	1	0.157	1267.0
4	0	3	0.367	2963.9
5	1	3	0.428	3452.5

Table S5. Composition of the three lowest spin-orbit coupled states of $[LCo_2(N_2)]^-$ based on CASSCF/NEVPT2 including the energy in Hartree units. The lowest eigenvalue of the SOC matrix is -4750.34798662 Eh.

	Energy (cm^{-1})	Weight	Real	Imag.	Root	Spin	M_s
State 0	0.00	0.443081	-0.664879	0.031898	2	1	1
		0.443081	-0.664879	-0.031898	2	1	-1
		0.094149	-0.306838	0.000000	0	0	0
		0.010911	0.104455	-0.000000	2	0	0
State 1	102.16	0.487379	0.035055	0.697245	2	1	1
		0.487379	0.035055	-0.697245	2	1	-1
State 2	103.52	0.973713	-0.000000	-0.986769	2	1	0
State 3	1213.40	0.013113	-0.114188	0.008625	0	1	1
		0.013113	-0.114188	-0.008625	0	1	-1
		0.178088	0.422005	-0.000000	0	0	0
		0.757166	0.870153	-0.000000	2	0	0
State 4	1398.00	0.016292	-0.127190	0.010696	2	1	1
		0.016292	-0.127190	-0.010696	2	1	-1
		0.284126	0.533035	0.000000	0	0	0
		0.106383	-0.326165	0.000000	2	0	0
		0.561894	-0.749596	0.000000	6	0	0
State 5	1450.16	0.034187	0.184743	-0.007575	2	1	1
		0.034187	0.184743	0.007575	2	1	-1
		0.420475	-0.648440	-0.000000	0	0	0
		0.075827	0.275368	-0.000000	2	0	0

Table S6. Configuration state functions for $[LCo_2(N_2)]^-$ at the CASSCF/NEVPT2 level of the most important states for the ground state identified in the SOC-CASSCF/NEVPT2 calculations: root2 (triplet), root0 (singlet), root2 (singlet) and root6 (singlet). The energies in Hartree units given are printed in italics for the CASSCF and bold for the CASSC/NEVPT2 level of theory.

Root 2, Multiplicity 3		Root 0, Multiplicity 1		Root 2, Multiplicity 1		Root 6, Multiplicity 1	
-4741.1273420727, **-4750.347103483**		*-4741.1309707775,* **-4750.341888**		*-4741.1267047376,* **-4750.341707**		*-4741.1082492115,* **-4750.341331**	
Weight	CSF	Weight	CSF	Weight	CSF	Weight	CSF
0.25419	222222112211	0.18307	222222211211	0.22044	222222112211	0.12374	222222202211
0.22674	222212212211	0.09315	222222221210	0.21928	222212212211	0.05949	222212222210
0.18456	222222212210	0.07975	222212221211	0.14174	222222212210	0.05374	222222222200
0.11697	222222212201	0.07546	222222121211	0.09025	222222212201	0.05361	222212222201
0.02685	222222112220	0.06248	222212212211	0.07856	222222211211	0.0535	222212122211
0.02470	222212212220	0.0557	222222112211	0.03154	222222221210	0.05066	222222122201
0.01983	222221122211	0.04209	222222212111	0.02596	222212221211	0.04368	222122212211
0.01904	222222112202	0.04072	222222221201	0.0235	222222121211	0.04031	222222122210
0.01737	222212212202	0.03335	222222212210	0.01965	222222221201	0.03498	222222202202
0.01480	222211222211	0.0326	222222212201	0.01716	222221122211	0.03217	222202222211
0.01026	222222211211	0.02783	222212222111	0.01579	222211222211	0.02964	222221212211
0.00751	222221222210	0.02171	222222222101	0.00886	222222211220	0.02811	222222202220
0.00528	112222112222	0.02141	222222222110	0.00811	222221222210	0.02394	222222211211
0.00493	112212212222	0.01832	222222122111	0.00808	222221221211	0.02144	222222022211
0.00467	222222121211	0.01705	222221221211	0.00566	222222211202	0.01968	221222222210
0.00452	222221222201	0.01422	222222211220	0.00551	222221222201	0.01907	222211222211
0.00431	222212112212	0.00999	222222222200	0.00383	112212212222	0.016	221212222211
0.00340	222212221211	0.00949	222222221111	0.00383	222222121220	0.01425	221222222201
0.00332	222212112221	0.00792	222212222201	0.00378	112222112222	0.01303	222222221201
0.00295	222202212212	0.00681	222222211202	0.0035	222212112212	0.01267	222212212211
		0.00667	222212222210	0.00341	222212221220	0.01261	222212122202
		0.00664	222222122201	0.00301	222212222111	0.01191	221222122211
		0.00653	222222202211	0.00288	222222222110	0.0101	222212122220
		0.00598	222211222211	0.00269	222212112221	0.00993	222212221211
		0.00558	222221222111			0.00866	222221222210
		0.00557	222221122211			0.00863	222221222201
		0.00402	222212221220			0.00815	222221212220
		0.00386	222222121220			0.00754	222222212111
		0.00331	222222122210			0.00741	222202222220
		0.00328	222222212120			0.00731	222222121211
		0.00324	222221222210			0.00702	222202222202
		0.00323	112222211222			0.00674	222122212220
		0.00306	222222212102			0.00658	222222211202
		0.00303	222212122211			0.00647	222221212202
		0.00277	222222202202			0.00586	222122212202
						0.00552	222121222211
						0.00538	222222221111
						0.00532	202222202222
						0.00531	222222112211
						0.0048	222212222111
						0.00479	222222022202
						0.00444	221222212211
						0.00418	222221122211
						0.00415	222222222101
						0.00391	222221221211
						0.0037	222222122111
						0.0036	222222022220
						0.0026	222222222110

SA-CASSCF/NEVPT2 results with 60 roots (30 singlet, 30 roots)

Decreasing the number of roots to 60 reduces the degeneracy for state 1, 2 and 3 at the SA-CASSCF/NEVPT2 level. Upon consideration of SOC, the ground state description shows the identical multireference character with a triply degenerate ground state as for the calculation with a larger number of roots.

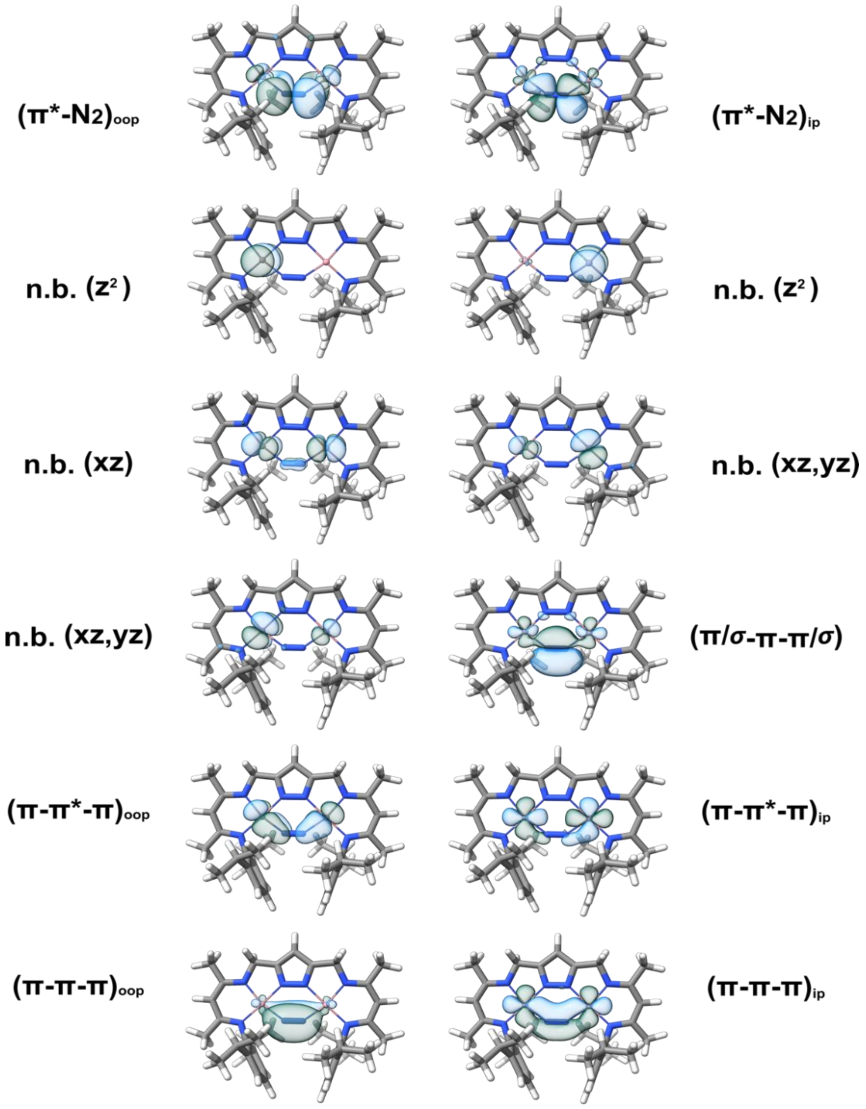

Figure S50: Active space orbitals of the SA-CASSCF over 60 states calculations with 20 electrons in 12 orbitals for complex $[LCo_2(N_2)]^-$.

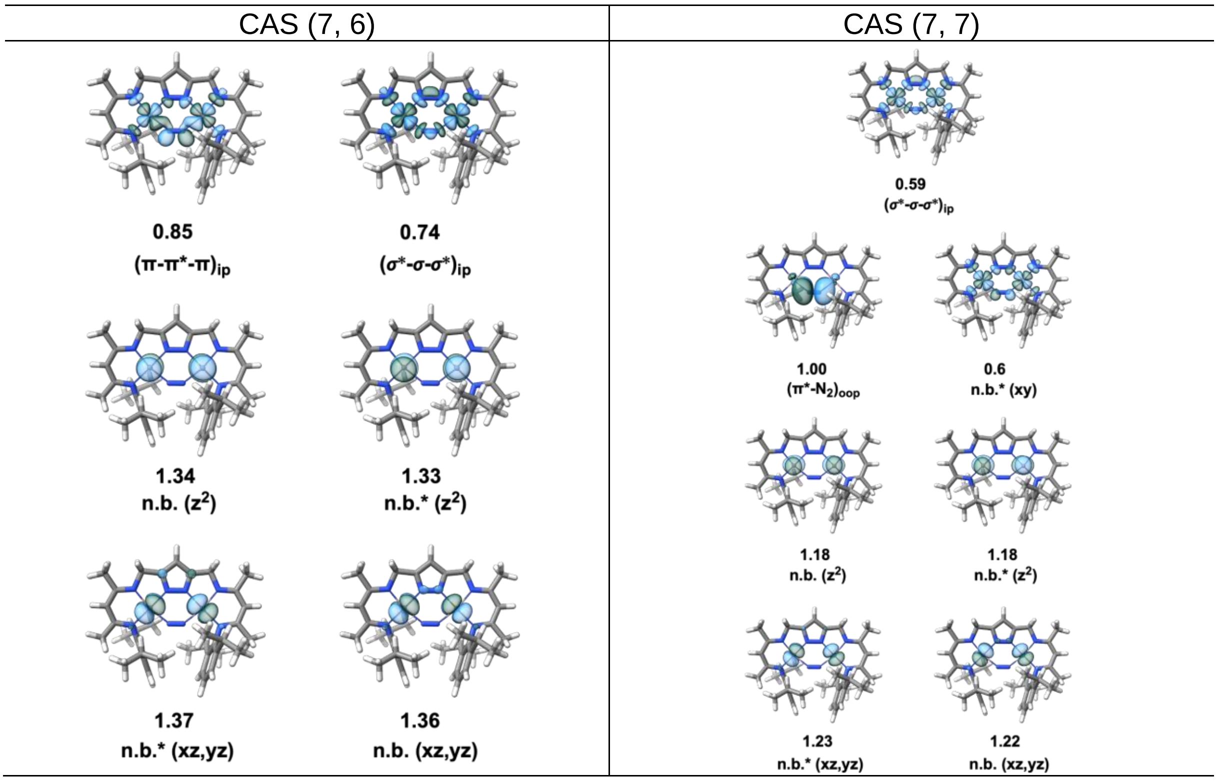

Figure S51: Molecular orbitals of the neutral complex [$LCo_2(N_2)$] using active spaces of size (7, 6) and (7, 7). Images are created in Chimera with isosurface value of 0.025. Further increasing this active space led to severe imbalances. It appears likely that a significant number of ligand orbitals should be included to achieve a balanced active space.

Table S7. CASSCF/NEVPT2 of [$LCo_2(N_2)$] corrected transition energies for the lowest root (root 2, multiplicity 3, -4750.347103483 Eh).

State	Root	Multiplicity	Δ*E*/eV	Δ*E*/cm^{-1}
0	0	2	0.000	0.0
1	1	4	0.000	0.0
2	2	4	0.009	78.13
3	1	2	0.009	78.13
4	2	2	0.061	495.82
5	3	4	0.061	495.82

Table S8. Spin-orbit coupled ground state for $[LCo_2(N_2)]$ with the active space (7,6) and the energetically lowest excited states.

	ΔE/cm^{-1}	Weight	Real	Imag.	Root	Spin	M_S
State 0	0.00	0.327388	-0.561508	-0.109983	0	3/2	3/2
		0.019583	-0.015923	0.139029	1	3/2	3/2
		0.034416	-0.027242	-0.183505	2	3/2	3/2
		0.306522	0.397540	0.385336	0	3/2	1/2
		0.016871	0.124824	-0.035921	1	3/2	1/2
		0.032580	-0.180120	0.011711	2	3/2	1/2
		0.138112	0.371529	0.008816	0	3/2	-1/2
		0.033103	-0.181916	0.003105	1	3/2	-1/2
		0.041589	-0.137517	0.150592	0	3/2	-3/2
		0.014518	0.117322	0.027452	2	3/2	-3/2
State 1	0.00	0.041589	0.200477	-0.037387	0	3/2	3/2
		0.014518	-0.038567	0.114152	2	3/2	3/2
		0.138112	0.188498	-0.320281	0	3/2	1/2
		0.033103	-0.098602	0.152908	1	3/2	1/2
		0.306522	0.117651	0.540999	0	3/2	-1/2
		0.016871	-0.096364	0.087094	1	3/2	-1/2
		0.032580	0.104966	-0.146842	2	3/2	-1/2
		0.327388	-0.202770	0.535044	0	3/2	-3/2
		0.019583	-0.126511	-0.059813	1	3/2	-3/2
State 2	78.13	0.08883	0.160932	-0.250861	0	3/2	3/2
		0.020537	-0.084421	-0.115801	1	3/2	3/2
		0.025212	-0.124754	0.098224	0	3/2	1/2
		0.018366	-0.037488	0.130233	2	3/2	1/2
		0.365563	-0.139085	-0.588404	0	3/2	-1/2
		0.390837	-0.331984	0.529740	0	3/2	-3/2
		0.010046	-0.040741	-0.091576	3	3/2	-3/2
		0.012247	-0.109917	-0.012862	0	1/2	1/2
		0.017517	0.119819	-0.056213	2	1/2	-1/2
State 3	78.13	0.390837	0.392419	0.486667	0	3/2	3/2
		0.010046	0.029602	-0.095759	3	3/2	3/2
		0.365563	-0.068378	0.600739	0	3/2	1/2
		0.025212	0.135515	0.082748	0	3/2	-1/2
		0.018366	0.052657	0.124873	2	3/2	-1/2
		0.088830	0.189525	0.230022	0	3/2	-3/2
		0.020537	-0.070103	0.124989	1	3/2	-3/2
		0.017517	-0.125636	-0.041618	2	1/2	1/2
		0.012247	-0.107619	0.025797	0	1/2	-1/2

Table S9: a) CASSCF/NEVPT2 corrected transition energies of [$LCo_2(N_2)$] for the lowest root (root 2, multiplicity 3, -4750.347103483 Eh)

**State**	**Root**	**MULT**	**ΔE/eV**	**ΔE/cm^{-1}**
0	0	2	0.000	0.0
1	1	2	0.000	0.0
2	1	4	0.059	47.29
3	2	4	0.059	47.29
4	3	4	0.0365	294.44
5	2	2	0.0365	294.44

Table S10. Spin-orbit coupled ground state for [$LCo_2(N_2)$] with the active space (7,7) and the energetically lowest excited states.

	**Δ*E*/cm^{-1}**	Weight	Real	Imag.	Root	Spin	M_s
State 0	0.00	0.426450	-0.182256	0.627083	0	3/2	1/2
		0.178231	0.036473	-0.420596	0	3/2	-1/2
		0.031429	-0.044490	0.171609	2	3/2	-1/2
		0.320222	0.118878	-0.553254	0	3/2	-3/2
State 1	0.00	0.320222	-0.028407	-0.565168	0	3/2	3/2
		0.178231	-0.031604	0.420990	0	3/2	1/2
		0.031429	-0.016329	-0.176529	2	3/2	1/2
State 2	47.29	0.531815	0.070624	0.725829	0	3/2	3/2
		0.269774	0.037478	0.518044	0	3/2	1/2
		0.068996	0.037428	0.259991	0	3/2	-1/2
		0.095125	0.006220	0.308361	0	3/2	-3/2
State 3	47.29	0.095125	0.010252	0.308253	0	3/2	3/2
		0.068996	0.023494	-0.261619	0	3/2	1/2
		0.269774	-0.009767	0.519306	0	3/2	-1/2
		0.531815	0.031771	-0.728564	0	3/2	-3/2
State 4	294.44	0.023792	-0.082705	0.130200	1	3/2	-3/2
		0.259672	-0.484460	-0.158021	0	1/2	1/2
		0.657950	-0.399396	0.705998	0	1/2	-1/2
State 5	294.44	0.023792	0.139696	-0.065401	1	3/2	3/2
		0.657950	0.751221	-0.305971	0	1/2	1/2
		0.259672	0.094862	0.500672	0	1/2	-1/2

Table S11. Energetic and thermodynamic data for all relevant computational models. Listed are the Final Single Point Energy (*FSPE*), enthalpy *H*, entropy contribution *TS* at *T*=298.15K, and Gibbs free energy *G*; all energies are given in Hartree units. Note that $[LCo_2(N_2)]^-$ has multi-reference and multiconfigurational character, so that the thermodynamic data derived from DFT calculations cannot be interpreted with full confidence.

	FSPE	***H***	***TS***	***G***
**$[LCo_2N_2]^-$ (2^{bare})**	-4757.94893139	-4757.06262701	0.12645131	-4757.189078
**$[LCo_2N_2]^-K^+$•2THF (2•THF)**	-5827.10366884	-5825.97222687	0.15689934	-5826.12912621
**$[LCo_2(N_2)_2]^-$ (5)**	-4867.63806034	-4866.73913515	0.13265769	-4866.87179284
**N_2**	-109.5788845	-109.5702196	0.0217496	-109.5919692
**$[LCo_2(N_2)]$ (6)**	-4757.83781882	-4756.94866362	0.12920252	-4757.07786614
**$[LCo_2(N_2)]$•THF (6•THF)**	-4990.56462220	-4989.55294789	0.13940907	-4989.69235696
**$[LCo_2(N_2)_2]$ (6•N_2)**	-4866.61713558	-4866.61713558	0.13292284	-4866.7500584
**(VI)**	-5133.42830764	-5132.68087033	0.12014777	-5132.80101811

(2^{bare}) No K^+ counterion or THF solvent molecules considered
(**2•THF**) One K^+ counterion with two THF solvent molecules situated above the $(N_2)_{pz}$–Co_2–N_2 plane
(**5**) Two N_2 bound end-on at Co cations; no K^+ counterions or THF solvent molecules considered
(**6**) Neutral complex, no K^+ counterion or THF solvent molecules considered
(**6•THF**) Neutral complex, THF molecule bound end-on at Co^1 center, N_2 is bound end-on at Co^2 center
(**6•N_2**) Neutral complex, two N_2 bound end-on at the cobalt cations; no K^+ counterions or THF solvent molecules considered
(VI) Dicobalt(I) complex of the pyrazolate ligand system with two PNN compartments

Table S12. Selected distances [Å] and angles [°] for all relevant computational models.

	**$[LCo_2(N_2)]^-$ (2^{bare})**	**$[LCo_2(N_2)]$ K•2THF (2•THF)**	**$[LCo_2(N_2)_2]^-$ (5)**	**$[LCo_2(N_2)]$ (6)**	**$[LCo_2(N_2)]$• THF (6•THF)**	**$[LCo_2(N_2)_2]$ (6•N_2)**	**(VI)**
Co···Co	4.006	4.009	4.226	4.003	4.43	4.182	4.179
N–$N^{(N2)}$	1.155	1.159	1.135, 1.135	1.147	1.138	1.125	1.137, 1.137
Co–$N^{(N2)}$	1.766, 1.765	1.769, 1.763	1.725, 1.725	1.775, 1.775	1.727	1.755, 1.755	1.710, 1.710
Co–N^{pz}	1.912, 1.912	2.730, 1.907	1.925, 1.925	1.892, 1,892	1.968, 1.934	1.898, 1.898	1.914, 1.914
Co···$O^{(THF)}$	–	–	–	–	1.986	–	–
Co···K	–	3.187, 3.525	–	–	–	–	–
$N^{(N2)}$···K	–	2.945, 3.021	–	–	–	–	–
K···$O^{(THF)}$	–	2.666, 2.681	–	–	–	–	–
Co–N–$N^{(N2)}$	143.89, 143.75	143.48, 143.91	170.32, 169.60	143.55, 143.5	162.16	169.95, 170.22	172.372, 172.343
Co–N–N^{pz}	133.83, 133.85	133.51, 133.70	134.52, 134.73	133.96, 133.98	136.09	133.47, 133.58	136.255, 136.239
$N^{(N2)}$–Co–N^{pz}	82.25, 82.29	82.46, 82.37	89.62, 89.83	82.32, 82.32		90.38, 90.44	98.45, 98.46

(2^{bare}) No K^+ counterion or THF solvent molecules considered
(**2•THF**) One K^+ counterion with two THF solvent molecules situated above the $(N_2)_{pz}$–Co_2–N_2 plane

(**5**) Two N_2 bound end-on at Co cations; no K^+ counterions or THF solvent molecules considered
(**6**) Neutral complex, no K^+ counterion or THF solvent molecules considered
(**6•THF**) Neutral complex, THF molecule bound end-on at Co^1 center, N_2 is bound end-on at Co^2 center
(**6•N$_2$**) Neutral complex, two N_2 bound end-on at the cobalt cations; no K^+ counterions or THF solvent molecules considered
(VI) Dicobalt(I) complex of the pyrazolate ligand system with two PNN compartments

Table S13. Selected vibrational modes (cm^{-1}) at the theory level of BP86/ZORA-def2-TZVP.

	**$[LCo_2(N_2)]^-$ (2^{bare})**	**$[LCo_2(N_2)]K$•2THF (2•THF)**	**$[LCo_2(N_2)_2]^-$ (5)**	**$[LCo_2(N_2)]$ (6)**	**$[LCo_2(N_2)(THF)]$ (6•THF)**	**$[LCo_2(N_2)_2]$ (6•N$_2$)**	**(VI)**
N-N stretch	1942	1922	2060 (anti-symm.)<br>2064 (symm.)	1983	2026	2078 (antisy-mmm.)<br>2122 (symm.)	2032 (anti-symm.)<br>2046 (symm.)
Co-N-N-Co zig-zag oop	348 / 349 / 362	355	–	311 / 328 / 394	–	–	–
Co-N-N-Co zig-zag ip	481	489	–	454 / 501	–	–	–

(**2^{bare}**) No K^+ counterion or THF solvent molecules considered
(**2•THF**) One K^+ counterion with two THF solvent molecules situated above the $(N_2)_{pz}$–Co_2–N_2 plane
(**5**) Two N_2 bound end-on at Co cations; no K^+ counterions or THF solvent molecules considered
(**6**) Neutral complex, no K^+ counterion or THF solvent molecules considered
(**6•THF**) Neutral complex, THF molecule bound end-on at Co^1 center, N_2 is bound end-on at Co^2 center
(**6•N$_2$**) Neutral complex, two N_2 bound end-on at the cobalt cations; no K^+ counterions or THF solvent molecules considered
(VI) Dicobalt(I) complex of the pyrazolate ligand system with two tridentate PNN compartments

Table S14. Mulliken spin populations of complexes $[LCo_2(N_2)]^-$ (singlet and triplet), $[LCo_2(N_2)]$, and $[LCo_2(N_2)]$•THF at the theory level of BP86/ZORA-def2-TZVP.

	**Multiplicity**	**Co^1**	**N^1**	**N^2**	**Co^2**
**$[LCo_2N_2]^-$** (**2^{bare}**)	singlet	0.00	0.00	0.00	0.00
**$[LCo_2N_2]^-$** (**2^{bare}**)	triplet	0.79	0.14	0.13	0.82
**$[LCo_2(N_2)]$** (**6**)	doublet	0.48	-0.05	-0.05	0.49
**$[LCo_2(N_2)]$•THF** (**6•THF**)	doublet	0.867	-0.001	-0.001	0.06

(**2^{bare}**) Singlet multiplicity and No K^+ counterion or THF solvent molecules considered
(**2^{bare}**) Triplet multiplicity and No K^+ counterion or THF solvent molecules considered
(**6**) Neutral complex, no K^+ counterion or THF solvent molecules considered
(**6•THF**) Neutral complex, THF molecule bound end-on at Co^1 center, N_2 is bound end-on at Co^2 center

8 Crystallographic Details

Crystal data and details of the data collections are given in Table S15, selected bond lengths angles in Table S16, molecular structures are shown in Figures S52 – S57. X-ray data were collected on a STOE IPDS II or a BRUKER D8-QUEST diffractometer (monochromated Mo-Kα radiation, λ = 0.71073 Å) by use of ω or ω and ϕ scans at low temperature. The structures were solved with SHELXT and refined on F^2 using all reflections with SHELXL.[15] Non-hydrogen atoms were refined anisotropically. Hydrogen atoms were placed in calculated positions and assigned to an isotropic displacement parameter of 1.5/1.2 U_{eq}(C).

Crystals of **1** and **2** were found to be twinned (twin law **1**: –1 0 0, 0 –1 0, 0 0 1; twin law **2**: –1 0 0, 0 –1 0, 0.505 0 1). **1** is also racemically twinned, so the three BASF parameters were refined to: 0.16(1), 0.33(1), and 0.12(1). BASF for **2** was refined to: 0.123(1). A potassium coordinating and a non-coordinating thf molecule were found to be disordered in **3**. The occupancy factors of the coordinating thf molecule are 0.751(16) / 0.249(16). The non-coordinating thf is disordered about a center of inversion and was refined at ½ occupancy. SAME and RIGU restraints were applied to model the disordered parts. In case of **4** parts of the potassium coordinating cryptand were found to be disordered (C51/C52A/B occupancy factors: 0.764(4) / 0.236(4); C57/C58A/B occupancy factors: 0.687(9) / 0.313(9)). SADI restraints and EADP constraints were applied to model the disorder. The crystals seem furthermore co-crystallized with the μ-Br-compound. After initial refinement the occupancy factors N_2 / Br were fixed at 0.95 / 0.05. Most bond lengths and angles are in good agreement with either the μ-N_2 or the μ-Br compound (Table S16). The Co···Co distance is closer to the μ-N_2 species, which is also the major component. **6** is probably also co-crystalized with LCo_2(μ-Br). The contribution of the second component is very small and could not be refined as disorder as in case of **4**. As a result, the distance between the nitrogen atoms, μ-N_2, is shorter than expected and cannot be considered reliable.

In case of [$LCo_2(N_2)$][$K(THF)_2$]·$KBEt_3H$ (**2**(THF)·$KBEt_3H$) (yw14) the arrangement of the atoms could be established, but the overall quality of the structure determinations prevents a detailed discussion of the bonding parameters (cell parameters: a = 14.1793(9) Å, b = 14.6000(10) Å, c = 17.0433(10) Å, α = 77.660(5)°, β = 83.293(5)°, γ = 106.256(4)°, V = 3409.2(4) Å^3, space group: P–1).

Face-indexed absorption corrections were performed numerically with the program X-RED[16] or by the multi-scan method with SADABS.[17]

CCDC 2245223 (**1**), 2245224 (**2**), 2245225 (**3**), 2245226 (**4**) and 2420497 (**6**) contain the supplementary crystallographic data for this paper. These data can be obtained free of charge from The Cambridge Crystallographic Data Centre via www.ccdc.cam.ac.uk/structures, or by emailing data_request@ccdc.cam.ac.uk, or by contacting The Cambridge Crystallographic Data Centre, 12 Union Road, Cambridge CB2 1EZ, UK; fax: +44 1223 336033.

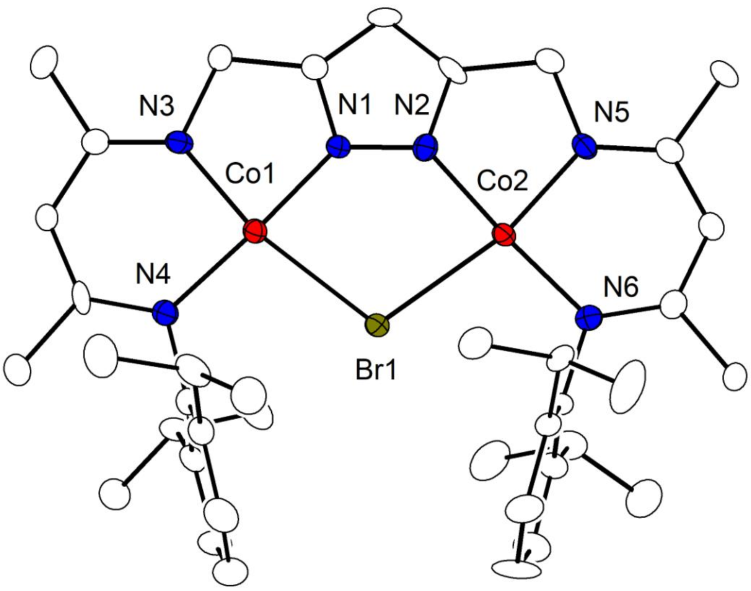

Figure S52. Plot (30% probability thermal ellipsoids) of the molecular structure of **1** (hydrogen atoms omitted for clarity). Only one of the four crystallographically independent molecules is shown. Selected bond lengths [Å] and angles [°]: Co1–N1 1.873(10), Co1–N3 1.870(13), Co1–N4 1.906(10), Co2–N2 1.866(10), Co2–N6 1.895(10), Co2–N5 1.898(9), Co1–Br1 2.4485(19), Co2–Br1 2.4544(19), Co1···Co2 3.890(2); N3–Co1–N1 83.3(5), N3–Co1–N4 93.3(5), N1–Co1–N4 176.1(5), N3–Co1–Br1 168.0(4), N1–Co1–Br1 84.7(3), N4–Co1–Br1 98.7(3), N2–Co2–N6 176.5(4), N2–Co2–N5 82.6(4), N6–Co2–N5 94.1(4), N2–Co2–Br1 85.2(3), N6–Co2–Br1 98.2(3), N5–Co2–Br1 167.7(3), Co1–Br1–Co2 105.02(7).

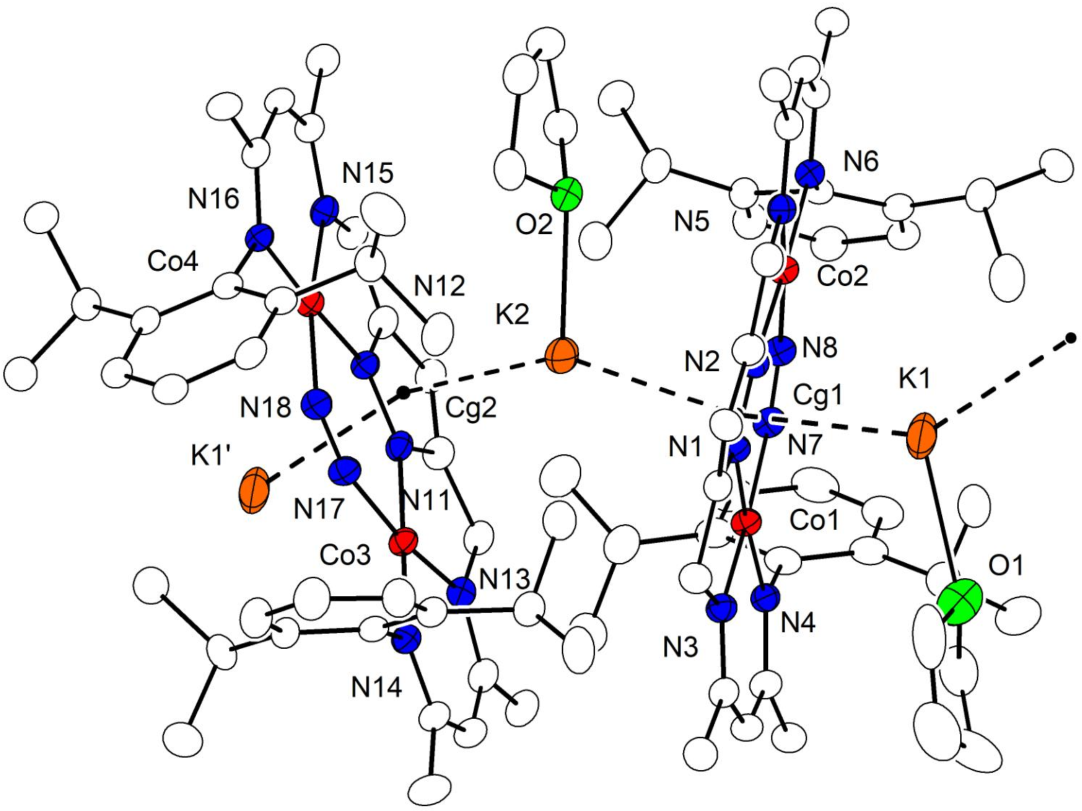

Figure S53. Plot (30% probability thermal ellipsoids) of the molecular structure of **2** (hydrogen atoms omitted for clarity). Cg is defined as the centroid of the five ring atoms. Selected bond lengths [Å] and angles [°]: Co1–N1 1.904(7), Co1–N3 1.886(7), Co1–N4 1.876(7), Co2–N2 1.895(7), Co2–N5 1.881(7), Co2–N6 1.879(6), Co1–N7 1.810(7), Co2–N8 1.805(7), Co3–N11 1.898(7), Co3–N13 1.871(7), Co3–N14 1.878(7), Co4–N12 1.909(6), Co4–N15 1.875(6), Co4–N16 1.888(6), Co3–N17 1.796(7), Co4–N18 1.802(7), Co1···Co2 4.0313(14), Co3···Co4 4.0133(14), N7–N8 1.144(10), N17–N18 1.135(9), K1–O1 2.660(10), K2–O2 2.688(7), K1–Cg1 2.920(2), K1'–Cg2 3.053(2), K2–Cg1 2.909(2), K2–Cg2 2.889(2); N7–Co1–N4 98.9(3), N7–Co1–N3 165.2(3), N4–Co1–N3 95.8(3), N7–Co1–N1 82.4(3), N4–Co1–N1 175.8(3), N3–Co1–N1 82.9(3), N8–Co2–N6 99.3(3), N8–Co2–N5 165.7(3), N6–Co2–N5 95.0(3), N8–Co2–N2 82.9(3), N6–Co2–N2 177.2(3), N5–Co2–N2 82.8(3), N17–Co3–N13 165.5(3), N17–Co3–N14 98.8(3), N13–Co3–N14 95.6(3), N17–Co3–N11 82.8(3), N13–Co3–N11 82.8(3), N14–Co3–N11 177.9(3), N18–Co4–N15 165.0(3), N18–Co4–N16 98.6(3), N15–Co4–N16 95.7(3), N18–Co4–N12 83.1(3), N15–Co4–N12 82.4(3), N16–Co4–N12 177.3(3), N8–N7–Co1 143.3(6), N7–N8–Co2 142.8(6), N18–N17–Co3 143.9(6), N17–N18–Co4 142.2(6). Symmetry transformation used to generate equivalent atoms: (') 1+*x*, *y*, *z*.

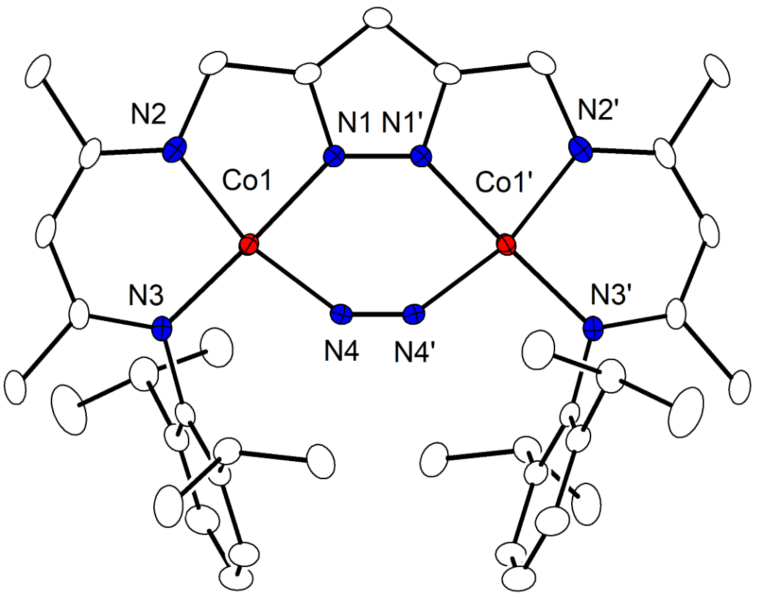

Figure S54. Plot (30% probability thermal ellipsoids) of the molecular structure of the anionic part of **3** (hydrogen atoms omitted for clarity). Selected bond lengths [Å] and angles [°]: Co1–N1 1.910(3), Co1–N2 1.883(3), Co1–N3 1.883(3), Co1–N4 1.803(3), Co1···Co2 4.0240(6), N4–N4' 1.138(5); N4–Co1–N3 99.33(11), N4–Co1–N2 164.86(12), N3–Co1–N2 95.52(12), N4–Co1–N1 82.67(11), N3–Co1–N1 177.94(11), N2–Co1–N1 82.50(12), C2–N1–Co1 117.9(2), N1'–N1–Co1 134.13(8). Symmetry transformation used to generate equivalent atoms: (') 1/2–*x*, 1/2–*y*, *z*.

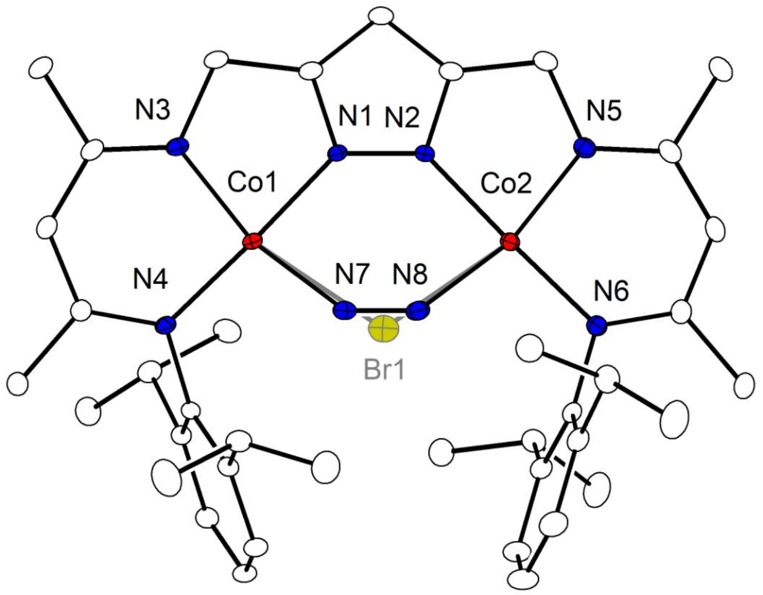

Figure S55. Plot (30% probability thermal ellipsoids) of the molecular structure of the anionic part of **4** (hydrogen atoms omitted for clarity, N7–N8 is disordered with Br1). Selected bond lengths [Å] and angles [°]: Co1–N1 1.9066(10), Co1–N3 1.8784(10), Co1–N4 1.8842(10), Co2–N2 1.9100(10), Co2–N5 1.8820(10), Co2–N6 1.8852(10), Co1–N7 1.7932(16), Co2–N8 1.7977(17), Co1–Br1 2.460(7), Co2–Br1 2.401(6), Co1···Co2 4.0199(7), N7–N8 1.148(2); N7–Co1–N3 165.25(6), N7–Co1–N4 99.19(6), N3–Co1–N4 95.35(4), N7–Co1–N1 82.94(6), N3–Co1–N1 82.63(4), N4–Co1–N1 176.89(4), N8–Co2–N5 165.15(7), N8–Co2–N6 99.00(7), N5–Co2–N6 95.71(5), N8–Co2–N2 82.81(7), N5–Co2–N2 82.53(4), N6–Co2–N2 177.79(4), N8–N7–Co1 143.28(11), N7–N8–Co2 142.92(13).

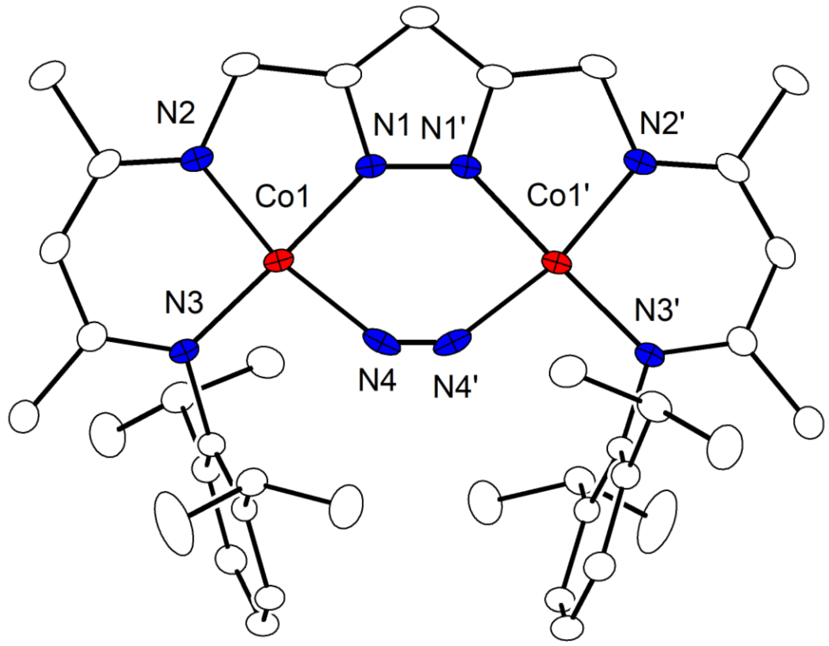

Figure S56. Plot (30% probability thermal ellipsoids) of the molecular structure of **6** (hydrogen atoms omitted for clarity). Selected bond lengths [Å] and angles [°]: Co1–N1 1.8834(18), Co1–N2 1.8755(18), Co1–N3 1.8730(19), Co1–N4 1.878(2), Co1···Co1' 3.9766(5), (N4–N4' 1.015(4))*; N3–Co1–N2 95.27(8), N3–Co1–N4 97.52(7), N2–Co1–N4 166.95(8), N3–Co1–N1 176.95(8), N2–Co1–N1 83.24(8), N4–Co1–N1 84.09(8), (N4'–N4–Co1 141.97(6))*. Symmetry transformation used to generate equivalent atoms: (') 1–*x*, *y*, 1/2–*z*.

*) **6** is probably co-crystalized with LCo_2(μ-Br). The contribution of the second component is very small and could not be refined as disorder as in case of **4**. As a result, the distance between the nitrogen atoms, μ-N_2 (N4–N4'), is shorter than expected and cannot be considered reliable.

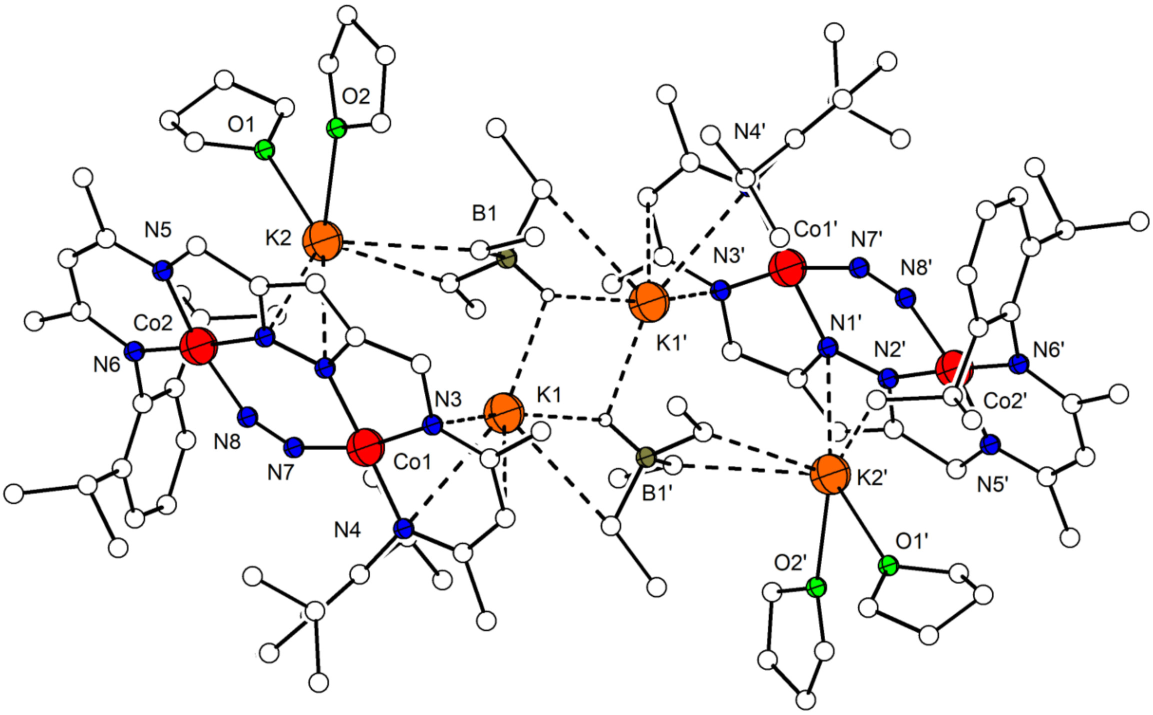

Figure S57. Plot of the molecular structure of $[LCo_2(N_2)][K(THF)_2]\cdot KBEt_3H$ (**2**(THF)·$KBEt_3H$) (most hydrogen atoms omitted for clarity). Symmetry transformation used to generate equivalent atoms: (') 2–*x*, 1–*y*, 1–*z*.

Table S15. Crystal data and refinement details for **1**, **2**, **3**, **4**, and **6**.

**compound**	**yw6 (1)**	**yw27 (2)**	**yw33_21 (3)**	**yw36_21 (4)**	**yw70a_23 (6)**
empirical formula	$C_{39}H_{53}BrCo_2N_6$	$C_{86}H_{122}Co_4K_2N_{16}O_2$	$C_{63}H_{101}Co_2KN_8O_9$	$C_{57}H_{89}Br_{0.05}Co_2KN_{9.90}O_6$	$C_{51}H_{65}Co_2N_8$
moiety formula	$C_{39}H_{53}BrCo_2N_6$	$C_{86}H_{122}Co_4K_2N_{16}O_2$	$C_{39}H_{53}Co_2N_8^-$, $C_{20}H_{40}KO_8^+$, C_4H_8O	$C_{39}H_{53}Br_{0.05}Co_2N_{7.90}^-$, $C_{18}H_{36}KN_2O^+$	$C_{51}H_{65}Co_2N_8$
formula weight	803.64	1725.91	1271.47	1169.93	907.97
T [K]	133(2)	133(2)	100(2)	100(2)	100(2)
crystal size [mm³]	0.380 x 0.220 x 0.200	0.500 x 0.190 x 0.140	0.340 x 0.283 x 0.123	0.354 x 0.335 x 0.331	0.349×0.177×0.080
crystal system	monoclinic	monoclinic	orthorhombic	monoclinic	monoclinic
space group	$P2_1$ (No. 4)	$P2_1/n$ (No. 14)	*Pccn* (No. 56)	$P2_1/n$ (No. 14)	*C*2/*c*
a [Å]	8.76540(10)	10.8273(2)	13.2422(4)	15.674(2)	11.2229(11)
b [Å]	28.7000(5)	15.7995(2)	21.9233(6)	14.7206(19)	29.479(3)
c [Å]	29.5346(5)	49.8052(9)	23.1518(7)	25.854(4)	14.3003(13)
β [°]	90.0710(10)	93.1460(10)	90	92.230(5)	95.507(3)
V [Å³]	7429.9(2)	8507.1(2)	6721.3(3)	5960.8(14)	4709.4(8)
Z	8	4	4	4	4
ρ [g·cm⁻³]	1.437	1.348	1.257	1.304	1.281
F(000)	3344	3648	2720	2492	1924
µ [mm⁻¹]	2.005	0.921	0.613	0.716	0.748
T_{min} / T_{max}	0.5620 / 0.6656*	0.6779 / 0.9349*	0.78 / 0.93$	0.72 / 0.80$	0.76 / 0.94
θ–range [°]	0.989 – 25.696	1.289 – 25.755	1.989 – 27.896	1.899 – 27.901	2.312 - 27.987$
hkl–range	–9 to 10, ±34, –35 to 36	–12 to 13, ±19, ±60	–16 to 17, ±28, ±30	±20, ±19, ±34	±14, ±38, ±18
measured refl.	66707	62531	74007	144574	54768
unique refl. [R_{int}]	27670 [0.0508]	15893 [0.0668]	8039 [0.0445]	14240 [0.0297]	5675 [0.0770]
observed refl. ($I > 2\sigma(I)$)	22848	13448	7023	13057	4389
data / restr. / param.	27670 / 1 / 1780	15893 / 0 / 1016	8039 / 100 / 445	14240 / 6 / 720	5675 / 0 / 283
goodness–of–fit (F^2)	1.040	1.074	1.259	1.025	1.040
*R*1, *wR*2 ($I > 2\sigma(I)$)	0.0470 / 0.1043	0.0876 / 0.2599	0.0649 / 0.1364	0.0274 / 0.0701	0.0445, 0.0971
*R*1, *wR*2 (all data)	0.0656 / 0.1147	0.1096 / 0.2990	0.0744 / 0.1402	0.0308 / 0.0721	0.0660, 0.1085
res. el. dens. [e·Å⁻³]	–0.615 / 1.311	–0.594 / 1.857	–0.509 / 0.524	–0.378 / 0.474	–0.578 / 0.487

*) X-RED. $) SADABS.

Table S16. Selected distances [Å] and angles [°].

	yw6* (1)	yw27 (2)	yw33_21 (3)	yw36_21** (4)	yw70a_23*** (6)
Co–N^{pz}	1.864(10) - 1.878(10)	1.895(7) - 1.909(6)	1.883(3)	1.9066(10) / 1.9100(10)	1.8834(18)
Co–N^{nacnac}	1.870(13) - 1.930(11)	1.871(7) - 1.888(6)	1.883(3) / 1.883(3)	1.8784(10) - 1.8852(10)	1.8730(19) / 1.8755(18)
Co–$N^{(N2)}$	-	1.796(7) - 1.810(7)	1.803(3)	1.7932(16) / 1.7977(17)	1.8834(18)
Co–Br	2.447(2) - 2.462(2)	-	-	2.401(6) / 2.460(7)	-
Co···Co	3.889(2) - 3.893(2)	4.0133(14) / 4.0313(14)	4.0240(6)	4.0199(7)	3.9766(5)
N–$N^{(N2)}$	-	1.135(9) / 1.144(10)	1.138(5)	1.148(2)	(1.015(4))***
Co–Br–Co	104.77(7) - 105.02(7)	-	-	111.5(3)	-
Co–N–$N^{(N2)}$	-	142.2(6) - 143.9(6)	143.18(8)	142.92(13) / 143.28(11)	(141.97(6))***
Co-Ct^{N2}-Co	-	123.44(4) / 123.32(4)	123.54(1)	123.60(1)	(119.73(1))***
τ_4	0.11 - 0.12	0.12 - 0.13	0.12	0.12 - 0.14	0.11

*) The asymmetric unit contains four crystallographically independent molecules.
**) Disorder of N7–N8 (-N_2-) and Br1.
***) **6** is probably co-crystalized with $LCo_2(\mu\text{-Br})$. The contribution of the second component is very small and could not be refined as disorder as in case of **4**. As a result, the distance between the nitrogen atoms, μ-N_2, is shorter than expected and cannot be considered reliable.

9 References

(1) Manz, D.-H.; Duan, P.-C.; Dechert, S.; Demeshko, S.; Oswald, R.; John, M.; Mata, R. A.; Meyer, F. Pairwise H_2/D_2 Exchange and H_2 Substitution at a Bimetallic Dinickel(II) Complex Featuring Two Terminal Hydrides. *J. Am. Chem. Soc.* **2017**, *139*, 16720–16731. DOI: 10.1021/jacs.7b08629.

(2) Bill, E. *julX, Max-Planck Institute for Chemical Energy Conversion, Mülheim/Ruhr*, **2008**.

(3) Kahn, O. *Molecular Magnetism*, VCH Publishers Inc., New York, **1993**.

(4) Stoll, S.; Schweiger A. EasySpin, A Comprehensive Software Package for Spectral Simulation and Analysis in EPR. *J. Magn. Res.* **2006**, *178*, 42-55. DOI: 10.1016/j.jmr.2005.08.013.

(5) Nishio, M. The CH/π Hydrogen Bond in Chemistry. Conformation, Supramolecules, Optical Resolution and Interactions Involving Carbohydrates. *Phys. Chem. Chem. Phys.* **2011**,*13*, 13873-13900. DOI: 10.1039/C1CP20404A.

(6) a) Banci, L.; Bencini, A.; Benelli, C.; Gatteschi, D.; Zanchini, C. Spectral-Structural Correlations in High-Spin Cobalt(II) Complexes. In: Structures Versus Special Properties. Structure and Bonding, **1982**, vol *52*. Springer, Berlin, Heidelberg. DOI: 10.1007/BFb0111296. b) Telser, J.; Goldfarb, D.; Stoll, S. EPR Interactions–Zero-Field Splittings. EPR Spectroscopy: Fundamentals and Methods. John Wiley & Sons, **2018**; pp 29-63. DOI: 10.1002/9780470034590.emrstm1501.

(7) Neese, F. The ORCA Program System. *WIREs Computational Molecular Science* **2012**, *2*, 73-78. DOI: 10.1002/wcms.81.

(8) a) Becke, A. D. Density-Functional Exchange-Rnergy Approximation With Correct Asymptotic Behavior. *Phys. Rev. A* **1988**, *38*, 3098-3100. DOI: 10.1103/PhysRevA.38.3098. b) Perdew, J. P. Density-Functional Approximation for The Correlation Energy of The Inhomogeneous Electron Gas. *Phys. Rev. B* **1986**, *33*, 8822-8824. DOI: 10.1103/PhysRevB.33.8822.

(9) Neese, F. An Improvement of The Resolution of The Identity Approximation for The Formation of The Coulomb Matrix. *J. Comp. Chem.* **2003**, *24*, 1740-1747. DOI: 10.1002/jcc.10318.

(10) a) Weigend, F.; Ahlrichs, R. Balanced Basis Sets of Split Valence, Triple Zeta Valence and Quadruple Zeta Valence Quality for H to Rn: Design and Assessment of Accuracy. *Phys. Chem. Chem. Phys.* **2005**, *7*, 3297. DOI: 10.1039/B508541A. b) Weigend, F. Accurate Coulomb-Fitting Basis Sets for H to Rn. *Phys. Chem. Chem. Phys.* **2006**, *8*, 1057. DOI: 10.1039/B515623H. c) Pantazis, D. A.; Chen, X. Y.; Landis, C. R.; Neese, F. All-Electron Scalar Relativistic Basis Sets for Third-Row Transition Metal Atoms. *J. Chem. Theory Comput.* **2008**, *4*, 908-919. DOI: 10.1021/ct800047t.

(11) a) Grimme, S.; Ehrlich, S.; Goerigk, L. Effect of the Damping Function in Ddispersion Corrected Density Functional Theory. *J. Comput. Chem.* **2011**, *32*, 1456-1465. DOI: 10.1002/jcc.21759. b) Grimme, S.; Antony, J.; Ehrlich, S.; Krieg, H. A Consistent and Accurate *ab initio* Parametrization of Density Functional Dispersion Correction (DFT-D) for the 94 Elements H-Pu. *J. Chem. Phys.* **2010**, *132*, 154104. DOI: 10.1063/1.3382344.

(12) Barone, V.; Cossi, M. Quantum Calculation of Molecular Energies and Energy Gradients in Solution by a Conductor Solvent Model. *J. Phys. Chem. A* **1998**, *102*, 1995-2001. DOI: 10.1021/jp9716997.

(13) a) Perdew, J. P.; Burke, K.; Ernzerhof, M. Generalized Gradient Approximation Made Simple. *Phys. Rev. Lett.* **1996**, *77*, 3865–3868. DOI: 10.1103/physrevlett.77.3865. b) Adamo, C.; Barone, V. Toward Reliable Density Functional Methods without Adjustable Parameters: The PBE0 Model. *J. Chem. Phys.* **1999**, *110*, 6158–6170. DOI: 10.1063/1.478522.

(14) Angeli, C.; Cimiraglia, R.; Evangelisti, S.; Leininger, T.; Malrieu, J.-P. Introduction of *n*-Electron Valence States for Multireference Perturbation. Theory. *J. Chem. Phys.* **2001**, *114*, 10252–10264. DOI: 10.1063/1.1361246.

(15) a) Sheldrick, G. M. SHELXT – Integrated Space-Group and Crystal-structure Determination. *Acta Cryst*. **2015**, *A71*, 3-8; b. DOI: 10.1107/S2053273314026370. b) Sheldrick, G. M. Crystal Structure Refinement with SHELXL. *Acta Cryst.* **2015**, *C71*, 3-8. DOI: 10.1107/S2053229614024218.

(16) *X-RED*; STOE & CIE GmbH, Darmstadt, Germany, **2002**.

(17) *SADABS*; BRUKER AXS GmbH, Karlsruhe, Germany, **2016**.

(18) Grimme, S.; Hansen, A. A Practicable Real-Space Measure and Visualization of Static Electron-Correlation Effects. *Angew. Chem. Int. Ed.* **2015**, *54*, 12308–12313. DOI: 10.1002/anie.201501887.
