# Supplementary material for: Severely Bent Dinitrogen Bridging in Highly Preorganized Dinuclear Cobalt Complexes Featuring an Intricate Electronic Structure
Source: JACS Au. 2025 Jun 20;5(7):3104–14. doi: 10.1021/jacsau.5c00129 (PMC12308450; doi:10.1021/jacsau.5c00129)
Supplement: Supplementary file 2 [file au5c00129_si_002.pdf]

Cartesian Coordinates in Å

$[LCo_2(N_2)]^-$ 2[bare]
(multiplicity = singlet)
FSPE= -4757.949024305736 Eh

Co	1.882211098	8.683894922	16.850710559
Co	1.164712787	9.213538971	20.757053677
N	1.554851586	10.212729603	17.937390355
N	1.269234170	10.388854520	19.262651694
N	2.091864982	9.971280269	15.509069082
N	2.140658777	7.207716374	15.737795818
N	0.723647182	10.787149989	21.668531156
N	1.125683151	8.107918914	22.260577209
N	1.704427220	7.844624265	18.395299064
N	1.496470054	7.996354754	19.521317653
C	1.231743165	12.418791762	18.279434199
H	1.141162197	13.488503588	18.117505780
C	1.541461468	11.425146844	17.336299292
C	1.067399025	11.709191021	19.480449148
C	1.853014097	11.379944210	15.885221618
H	1.020647436	11.798492870	15.290577089
H	2.737936715	12.000865483	15.658222079
C	2.445624439	9.733916633	14.235367744
C	2.641354203	8.437664979	13.732326125
H	2.933235034	8.356810221	12.685903794
C	2.461011165	7.233506206	14.433621253
C	2.637219647	10.889009175	13.279645548
H	2.921475740	10.518719018	12.287920199
H	3.421783031	11.579844510	13.628426791
H	1.716326213	11.484750841	13.174364351
C	2.595072329	5.942891600	13.655746217
H	3.117780177	6.126877598	12.708836682
H	1.606627955	5.516450628	13.419212509
H	3.141121621	5.175842393	14.220562425
C	1.867954037	5.930500615	16.325654955
C	0.602739718	5.331148777	16.127700319
C	0.353007983	4.089178258	16.724910316
H	-0.619352752	3.612825726	16.589788659
C	1.321930562	3.456191126	17.504441779
H	1.106528323	2.491507939	17.967902831
C	2.557469619	4.070466140	17.707683183
H	3.303744267	3.581257032	18.335739842
C	2.851724822	5.311186907	17.128230952
C	-0.476968026	6.052102456	15.333802871
H	0.032567626	6.714027704	14.618828949
C	-1.304992381	6.956862604	16.262631499
H	-2.054371703	7.521397941	15.686494424
H	-0.645668535	7.675893705	16.776063652

H	-1.829069573	6.358552714	17.022474044
C	-1.379059422	5.107185591	14.532120309
H	-2.058623873	5.689291514	13.892263861
H	-2.002693168	4.481425984	15.188057988
H	-0.788812439	4.439231794	13.886955202
C	4.199021993	5.976990161	17.351121935
H	4.046938710	7.050371830	17.153327788
C	4.697089685	5.837510360	18.792929700
H	5.598743963	6.450583481	18.939097623
H	4.961698140	4.798150583	19.040444534
H	3.938496827	6.170911336	19.512080019
C	5.252662947	5.459679288	16.357530951
H	6.217702753	5.962163237	16.525247792
H	4.949694115	5.644749353	15.318123401
H	5.405857548	4.375839478	16.480689061
C	0.732341326	12.041776609	20.888107170
H	1.467165393	12.751280330	21.310368366
H	-0.250264489	12.543188375	20.945223228
C	0.385623130	10.880689468	22.964935702
C	0.393317502	9.775738392	23.831430840
H	0.097540200	9.960119355	24.863553086
C	0.781589355	8.464588826	23.509243054
C	-0.020659737	12.216966839	23.543081805
H	-0.259765490	12.113257124	24.607786547
H	-0.904057678	12.629042123	23.029231625
H	0.781358848	12.965618355	23.441597615
C	0.845078918	7.455213849	24.634050584
H	0.310274472	7.836291835	25.512876895
H	1.887107864	7.254910752	24.931818157
H	0.410615097	6.488735587	24.345500833
C	1.625824600	6.779031446	22.071364273
C	0.779708015	5.786136653	21.530019363
C	1.297615876	4.498911298	21.338054037
H	0.658676619	3.722130090	20.914401948
C	2.618200411	4.196387737	21.668398247
H	3.007018690	3.189413768	21.505453594
C	3.446494937	5.191308674	22.189374677
H	4.484742767	4.954109475	22.426657238
C	2.972061993	6.492791456	22.395592050
C	-0.661009658	6.103513846	21.168864892
H	-0.726620215	7.200038437	21.088890863
C	-1.066799315	5.510395019	19.815595249
H	-2.065375600	5.871262444	19.527609032
H	-1.108596693	4.411048515	19.846268795
H	-0.359176285	5.794973904	19.026912962
C	-1.629342318	5.649757074	22.274343019
H	-2.666585731	5.900130404	22.003357129
H	-1.402440441	6.135734258	23.233134601
H	-1.569074958	4.559869506	22.422155928
C	3.895795412	7.591004468	22.902282447
H	3.265716408	8.349635406	23.388194992

C	4.594694821	8.288472150	21.722212159
H	5.220505711	9.119836490	22.081724009
H	3.844913527	8.693313868	21.023069705
H	5.236508246	7.580292997	21.177667006
C	4.914676310	7.099889916	23.937031743
H	5.470349176	7.954292385	24.350968147
H	5.652020941	6.413848053	23.493461947
H	4.420885611	6.576282646	24.769294890

[$LCo_2 (N_2)]^-$ 2^{bare}
(multiplicity = triplet)
FSPE= -4757.930942731703 Eh

Co	1.915486000	8.693654000	16.890478000
Co	1.102695000	9.203200000	20.721961000
N	1.618019000	10.202370000	17.957719000
N	1.257222000	10.371932000	19.268425000
N	2.203260000	9.984369000	15.529808000
N	2.131674000	7.201814000	15.744553000
N	0.644532000	10.785663000	21.665137000
N	1.101016000	8.097904000	22.258028000
N	1.628882000	7.699941000	18.390191000
N	1.413064000	7.847856000	19.541393000
C	1.304663000	12.408979000	18.302682000
H	1.236200000	13.481148000	18.145011000
C	1.653783000	11.421956000	17.366997000
C	1.061617000	11.695403000	19.487052000
C	2.032919000	11.395304000	15.929699000
H	1.257774000	11.882671000	15.310888000
H	2.964481000	11.966187000	15.761164000
C	2.550585000	9.747940000	14.256586000
C	2.688027000	8.444802000	13.750334000
H	2.975175000	8.357392000	12.702997000
C	2.460120000	7.241961000	14.439339000
C	2.794748000	10.898956000	13.308002000
H	3.076738000	10.525940000	12.316569000
H	3.597934000	11.560119000	13.671243000
H	1.895636000	11.526276000	13.197810000
C	2.559414000	5.960130000	13.640002000
H	3.061946000	6.148905000	12.683187000
H	1.560115000	5.549757000	13.423520000
H	3.108484000	5.179499000	14.183199000
C	1.840793000	5.915043000	16.298089000
C	0.565619000	5.342013000	16.092749000
C	0.293740000	4.093327000	16.666194000
H	-0.687129000	3.637141000	16.522905000
C	1.251503000	3.429186000	17.433336000
H	1.020883000	2.458485000	17.876629000
C	2.493941000	4.023082000	17.654920000
H	3.226940000	3.513590000	18.282097000
C	2.807615000	5.271520000	17.102487000
C	-0.508205000	6.100159000	15.326384000

H	0.004599000	6.781100000	14.632017000
C	-1.323650000	6.978949000	16.291485000
H	-2.080762000	7.559682000	15.742103000
H	-0.659032000	7.681668000	16.818455000
H	-1.837505000	6.358170000	17.040669000
C	-1.423146000	5.192258000	14.496565000
H	-2.097100000	5.802302000	13.877075000
H	-2.052890000	4.552528000	15.132865000
H	-0.841714000	4.538701000	13.829017000
C	4.151838000	5.929383000	17.362389000
H	3.980970000	7.015171000	17.271284000
C	4.682403000	5.660748000	18.773128000
H	5.578067000	6.271654000	18.959693000
H	4.968509000	4.606941000	18.914799000
H	3.934150000	5.910480000	19.536584000
C	5.192849000	5.531643000	16.302456000
H	6.156331000	6.023350000	16.507697000
H	4.871621000	5.824312000	15.293910000
H	5.357466000	4.442423000	16.306976000
C	0.651360000	12.033397000	20.875923000
H	1.343944000	12.770241000	21.321628000
H	-0.347524000	12.507316000	20.882197000
C	0.301160000	10.880256000	22.957505000
C	0.338014000	9.772540000	23.821290000
H	0.046364000	9.951798000	24.855656000
C	0.760998000	8.470673000	23.506434000
C	-0.133182000	12.207777000	23.535162000
H	-0.390625000	12.099391000	24.595209000
H	-1.011047000	12.609008000	23.003908000
H	0.661530000	12.966164000	23.449306000
C	0.869427000	7.483865000	24.648876000
H	0.354134000	7.872115000	25.536148000
H	1.922920000	7.305221000	24.917814000
H	0.443738000	6.505487000	24.389018000
C	1.629314000	6.778085000	22.100576000
C	0.808090000	5.752323000	21.582546000
C	1.357887000	4.473490000	21.420611000
H	0.736998000	3.670706000	21.018897000
C	2.686920000	4.212540000	21.751679000
H	3.099827000	3.211271000	21.614376000
C	3.495061000	5.243747000	22.234421000
H	4.542181000	5.041242000	22.464741000
C	2.987916000	6.537619000	22.407962000
C	-0.642418000	6.024591000	21.224837000
H	-0.733156000	7.116208000	21.113842000
C	-1.046694000	5.385307000	19.892128000
H	-2.057144000	5.713634000	19.605575000
H	-1.061884000	4.286233000	19.951364000
H	-0.353839000	5.669383000	19.090197000
C	-1.592071000	5.582275000	22.351304000
H	-2.636913000	5.801451000	22.082054000

H	-1.369126000	6.100566000	23.294060000
H	-1.506677000	4.498704000	22.530151000
C	3.892795000	7.680022000	22.845485000
H	3.257264000	8.439156000	23.323656000
C	4.529026000	8.346979000	21.612735000
H	5.133607000	9.217414000	21.910802000
H	3.743837000	8.688384000	20.918819000
H	5.179958000	7.636441000	21.081829000
C	4.961446000	7.262094000	23.861625000
H	5.504570000	8.148607000	24.221300000
H	5.704072000	6.580656000	23.419770000
H	4.513399000	6.758282000	24.731193000

[$LCo_2(N_2)$]K•2THF (2•THF)
FSPE= -5827.104860935605 Eh

Co	1.808056826	9.080984291	17.147030573
Co	0.992148761	9.373869048	21.061578036
N	1.250755649	10.514180910	18.275089794
N	0.894670806	10.591673398	19.597045832
N	1.829312666	10.428335697	15.849324705
N	2.267167407	7.693341147	15.990719133
N	0.231991796	10.812682043	21.991378143
N	1.087090431	8.216066334	22.528516670
N	1.746328850	8.171821972	18.664007209
N	1.544059900	8.269630970	19.801666979
C	0.460711285	12.592608589	18.649421633
H	0.145562916	13.620973155	18.504528373
C	1.003396721	11.715179665	17.698104526
C	0.407838115	11.832733495	19.829588556
C	1.340474352	11.762929398	16.253192328
H	0.454183184	12.041917628	15.656114668
H	2.100850522	12.539765286	16.056954433
C	2.241651946	10.289168753	14.578984277
C	2.652131420	9.057094328	14.048491914
H	2.983152063	9.056620099	13.011188125
C	2.617793353	7.814131763	14.701080491
C	2.258054012	11.486487152	13.658670308
H	2.623189895	11.198303570	12.666606204
H	2.905595302	12.289084763	14.045372399
H	1.251863219	11.919607505	13.544106356
C	2.945786806	6.581506312	13.890800044
H	3.401345905	6.865335443	12.934523735
H	2.037551870	5.996002864	13.677216034
H	3.630836007	5.912479573	14.428292045
C	2.078070501	6.368184110	16.507979936
C	0.853012631	5.706027924	16.258587369
C	0.662917505	4.431883356	16.806710875
H	-0.278545412	3.908120761	16.635669862
C	1.651865124	3.824207140	17.582217520
H	1.480541733	2.834203297	18.008563101
C	2.852179122	4.490937172	17.820392822

H	3.621149860	4.016221403	18.432614626
C	3.086187775	5.765900666	17.289819563
C	-0.245400029	6.392244136	15.459268189
H	0.246770027	7.073803302	14.749944588
C	-1.115556253	7.261923311	16.383319385
H	-1.875451930	7.805194052	15.801162347
H	-0.488636016	8.000150374	16.912263040
H	-1.628024921	6.639589034	17.131344064
C	-1.107352993	5.420882869	14.645562008
H	-1.798059258	5.984331452	14.001298894
H	-1.717873445	4.774149499	15.293244040
H	-0.490554427	4.774136911	14.004031519
C	4.402577422	6.474421843	17.551303888
H	4.272294032	7.505670892	17.193788183
C	4.750004742	6.523237928	19.044405182
H	5.634498884	7.158354547	19.209326171
H	4.993141607	5.526110997	19.438859042
H	3.914054529	6.905457182	19.644772354
C	5.557114738	5.843068209	16.756663172
H	6.495252446	6.387474436	16.943485544
H	5.362330726	5.863728602	15.675721677
H	5.706331066	4.794140554	17.055455640
C	-0.074243026	12.032722498	21.217715857
H	0.392670792	12.920314888	21.676998849
H	-1.162598937	12.223851868	21.219329655
C	-0.100445993	10.821682915	23.292468092
C	0.119023795	9.723994145	24.136200268
H	-0.195346825	9.834249610	25.172899564
C	0.674465375	8.484498533	23.777500273
C	-0.756400789	12.042963510	23.893498896
H	-0.970715036	11.874937585	24.954757470
H	-1.702473819	12.282911412	23.382759232
H	-0.117291539	12.936278504	23.809292263
C	0.804111287	7.443110318	24.865866720
H	0.175092378	7.712901163	25.722756389
H	1.842492805	7.365462863	25.220838533
H	0.516187874	6.443686735	24.514132006
C	1.637653674	6.914354458	22.276595859
C	0.836857949	5.951607589	21.616289921
C	1.390743392	4.696244407	21.342130367
H	0.790768499	3.947579714	20.823379853
C	2.701943323	4.388381935	21.708095345
H	3.118422912	3.405993455	21.478444531
C	3.480531268	5.347634759	22.351126553
H	4.512198657	5.112812337	22.620071993
C	2.968427290	6.619454554	22.644260689
C	-0.595452114	6.272091246	21.225667782
H	-0.647345014	7.367755773	21.111784496
C	-1.002941439	5.648962990	19.888213987
H	-1.991010576	6.024836218	19.584544466
H	-1.072340689	4.552013611	19.946265054

H	-0.284292505	5.898651462	19.096781532
C	-1.576098898	5.874783217	22.341702430
H	-2.607556479	6.131209837	22.055428370
H	-1.345071579	6.394978296	23.281028115
H	-1.533022533	4.790712272	22.531194868
C	3.871875409	7.662368768	23.284103119
H	3.244380882	8.520398466	23.563174441
C	4.899251175	8.159530139	22.258902367
H	5.495926018	8.991783546	22.656937881
H	4.369294795	8.502037878	21.362889513
H	5.580323454	7.351330237	21.956257816
C	4.584708571	7.151967390	24.544870859
H	5.161203386	7.965112443	25.010219309
H	5.287257875	6.340260575	24.304821999
H	3.871097779	6.769863472	25.288193445
K	3.982834985	9.980641205	19.297122362
O	4.624561609	11.692582814	21.258727542
C	4.590772516	13.108954305	20.957218655
H	4.688070238	13.224499637	19.868030123
H	5.446042271	13.612480211	21.442213763
C	3.262795272	13.599650529	21.515334194
H	2.449352385	13.332059707	20.824640222
H	3.244167237	14.684936200	21.678655922
C	3.164337630	12.789081450	22.816020931
H	2.135683587	12.670543125	23.176855443
H	3.752101948	13.272316011	23.609929098
C	3.779586779	11.443375195	22.427823612
H	4.398904797	11.006699475	23.223819067
H	3.004000257	10.712794989	22.127737096
H	4.488200986	9.456104777	16.206918619
O	5.827224603	10.477991335	17.437114243
C	6.122408097	11.873589824	17.177448308
C	5.464391073	12.177953217	15.839112783
C	5.714378980	10.870250463	15.075865678
C	5.530646430	9.814495980	16.165186909
H	5.729320274	12.467437034	18.015385925
H	7.216435721	12.016057151	17.130408892
H	5.891702763	13.060215069	15.345505973
H	4.384973913	12.340526197	15.979372320
H	6.741320902	10.851784696	14.681928335
H	5.017528084	10.712991824	14.244001249
H	6.205999881	8.953851705	16.059355509

$[LCo_2(N_2)_2]^-$ (5)
FSPE= -4867.638169148290 Eh

Co	1.663370380	8.396949395	16.792796741
Co	1.459803392	9.019803385	20.969064248
N	1.668872234	9.861687621	18.042549295
N	1.245964444	10.022827206	19.339192448
N	2.275600389	9.709782863	15.590375844
N	1.843182992	6.990198333	15.541824301

N	0.567730588	10.493184079	21.729382177
N	1.478335567	8.041914617	22.588850377
C	1.266840645	12.062537267	18.365645674
H	1.169250577	13.131029258	18.200948037
C	1.701016830	11.085602974	17.461108761
C	0.984658801	11.338682111	19.530754662
C	2.164705817	11.102401404	16.054841216
H	1.462761169	11.663666830	15.413125853
H	3.135536926	11.626611150	15.984190561
C	2.750690440	9.530911647	14.349435209
C	2.818906270	8.268869165	13.746591916
H	3.229881918	8.222281821	12.739357562
C	2.351699312	7.068046533	14.300225749
C	3.213779064	10.717911929	13.537476861
H	3.575020875	10.388821662	12.556481191
H	4.027208771	11.262699478	14.042144709
H	2.398568822	11.441940504	13.379457395
C	2.392421684	5.842643595	13.412577735
H	3.091794603	6.004152241	12.582683423
H	1.400198025	5.638749715	12.981505475
H	2.690783497	4.939867296	13.959716798
C	1.310316553	5.713606242	15.924253839
C	0.007872512	5.353203222	15.513906102
C	-0.506590116	4.117688212	15.930225836
H	-1.517866606	3.830170478	15.635426861
C	0.252397965	3.249837142	16.712657157
H	-0.161266970	2.289168906	17.025506356
C	1.539085547	3.619096040	17.107354073
H	2.118688147	2.940708117	17.733619266
C	2.087604512	4.852997743	16.736389686
C	-0.848326830	6.283506491	14.666717153
H	-0.189831826	7.068237828	14.269087701
C	-1.921206836	6.983140032	15.518714972
H	-2.537194477	7.644150212	14.889934094
H	-1.460583107	7.588682555	16.308944404
H	-2.586178324	6.244919012	15.993154929
C	-1.496002684	5.558923547	13.477048827
H	-2.009499873	6.282683833	12.826887282
H	-2.244265185	4.824761109	13.811620533
H	-0.748633489	5.024629056	12.872654693
C	3.470122026	5.272852852	17.212332503
H	3.400664504	6.354201261	17.422834958
C	3.907339923	4.570834097	18.501058520
H	4.844515385	5.015018549	18.863913963
H	4.089908135	3.496437371	18.340605983
H	3.162297415	4.677837134	19.300266734
C	4.548937097	5.082631239	16.130630884
H	5.534639912	5.360422782	16.534017964
H	4.361284541	5.705824285	15.248155458
H	4.597671543	4.030511636	15.808089400
C	0.478588696	11.690346488	20.877513922

H	1.065941776	12.515583491	21.318237534
H	-0.562129066	12.055496071	20.804666555
C	0.049392972	10.595387805	22.961269473
C	0.163531768	9.571352840	23.909704320
H	-0.301114383	9.739309535	24.880072153
C	0.884125033	8.379036290	23.746151082
C	-0.666970999	11.858560990	23.379331287
H	-1.016676075	11.771276914	24.414328528
H	-1.536796086	12.062622805	22.735050207
H	-0.007546102	12.737981439	23.309752343
C	1.022355134	7.490184854	24.963833432
H	0.237671902	7.729720430	25.692419020
H	1.995903470	7.645791058	25.455121462
H	0.963360743	6.424422137	24.712031463
C	2.300250236	6.866004472	22.623268207
C	1.798677335	5.649261939	22.104558701
C	2.634903014	4.525794671	22.124805299
H	2.269058594	3.578697589	21.727489495
C	3.935815262	4.600235241	22.624736533
H	4.575126371	3.715300781	22.619774787
C	4.418881998	5.811136370	23.118065856
H	5.441516177	5.869318310	23.495652232
C	3.613208183	6.957629199	23.134815094
C	0.398111780	5.577439529	21.516166903
H	0.229435042	6.548130548	21.019792910
C	0.241728604	4.481081328	20.459664267
H	-0.739690239	4.571779420	19.974134975
H	0.302720036	3.473049492	20.899537699
H	1.003945582	4.559594649	19.674070504
C	-0.685549306	5.414757474	22.597205054
H	-1.676853287	5.340817018	22.124567925
H	-0.707788078	6.264219859	23.290265535
H	-0.517960351	4.495785640	23.181202820
C	4.171678336	8.274421905	23.654069862
H	3.320246037	8.944059572	23.838028704
C	5.052926025	8.952721221	22.591723667
H	5.466573593	9.895502477	22.981562554
H	4.467552747	9.178918944	21.690752333
H	5.892716992	8.301178785	22.304963346
C	4.937110141	8.111790357	24.975311097
H	5.224478110	9.098007436	25.368872700
H	5.859620661	7.527612459	24.840250596
H	4.324571555	7.604280999	25.734994882
N	2.632224815	8.019626431	20.193747570
N	3.501938445	7.523951676	19.658972349
N	0.674138051	7.490382582	17.876894208
N	-0.109216345	7.049892372	18.570181088

[LCo$_2$(N$_2$)] (6)
FSPE= -4757.837979652674 Eh

Co	1.921051307	8.687703481	16.862931439

Co	1.148963856	9.215231149	20.756174409
N	1.559415412	10.201338222	17.940375369
N	1.235738818	10.373890370	19.262482753
N	2.114278697	9.958311443	15.520277120
N	2.171007881	7.222101398	15.752620489
N	0.669046758	10.765685752	21.661158652
N	1.132880821	8.124458723	22.256225908
N	1.749319382	7.839865861	18.412841558
N	1.547445554	7.993906456	19.531485430
C	1.174288177	12.395121234	18.271951091
H	1.061145168	13.461201398	18.105032116
C	1.527616515	11.409503620	17.338222843
C	1.000561038	11.687169858	19.470472313
C	1.864261730	11.367396808	15.895125097
H	1.040572601	11.779534932	15.287380281
H	2.752607175	11.986525306	15.684093485
C	2.478480945	9.726043445	14.248243961
C	2.684709815	8.434647528	13.747976394
H	2.978434319	8.352900285	12.703179918
C	2.496871944	7.237117928	14.450919235
C	2.665808941	10.883570517	13.298635254
H	2.959286445	10.517198633	12.309057217
H	3.442593062	11.576921581	13.656996766
H	1.739556924	11.468898867	13.191116501
C	2.623227604	5.941186523	13.687229737
H	3.130703703	6.115171513	12.731261760
H	1.629524864	5.516659705	13.474392719
H	3.176032578	5.183106090	14.255820819
C	1.871965222	5.945912314	16.346833548
C	0.578722067	5.402843282	16.179354199
C	0.291651037	4.181409103	16.800485747
H	-0.703016819	3.746403323	16.695019176
C	1.251620186	3.518812722	17.565913479
H	1.004843752	2.572498531	18.050346484
C	2.520583059	4.073561774	17.721815864
H	3.264676080	3.555818761	18.329112143
C	2.855335370	5.292026000	17.117165386
C	-0.484285633	6.149709072	15.387260273
H	0.036569355	6.809233332	14.678494191
C	-1.310653200	7.057019195	16.314930163
H	-2.028483498	7.653367132	15.731698339
H	-0.654119920	7.748532047	16.865516379
H	-1.872019297	6.457366772	17.045984741
C	-1.392264975	5.222196692	14.571200526
H	-2.059248341	5.818144685	13.931304637
H	-2.027887255	4.599185595	15.217499195
H	-0.804564930	4.553268188	13.925354529
C	4.247419205	5.877938621	17.285989774
H	4.208667926	6.910021424	16.907169259
C	4.674273483	5.934900748	18.758029382
H	5.637145684	6.458114118	18.853184674

H	4.797448328	4.928330817	19.183628108
H	3.935022237	6.464086973	19.371011427
C	5.282185807	5.103533026	16.452157960
H	6.280251795	5.552476568	16.566860772
H	5.027092491	5.109116200	15.383465544
H	5.339982538	4.054775037	16.781905880
C	0.632759288	12.014619883	20.868695258
H	1.330290133	12.755464730	21.295303457
H	-0.372757163	12.467009955	20.909858375
C	0.329483423	10.861773441	22.957322348
C	0.362777802	9.765003712	23.827528293
H	0.067818832	9.946748005	24.859239746
C	0.776140433	8.465949885	23.504550977
C	-0.103573135	12.192328571	23.522548895
H	-0.344278766	12.090928860	24.586233596
H	-0.991956907	12.579430673	23.000033347
H	0.686202552	12.952096878	23.416685413
C	0.846648409	7.445739895	24.614670286
H	0.365701332	7.836654100	25.518615281
H	1.892194121	7.201513455	24.857556005
H	0.361713378	6.502553545	24.330288737
C	1.644994547	6.794696723	22.055024010
C	0.795714657	5.795860255	21.536389664
C	1.335132316	4.523589329	21.305395614
H	0.697279926	3.739123571	20.894955732
C	2.672542372	4.247638027	21.583127233
H	3.077177570	3.252712703	21.389709899
C	3.496816669	5.249294264	22.096703094
H	4.545692590	5.029242733	22.299252144
C	3.004264717	6.537274772	22.339235767
C	-0.667305599	6.074963700	21.233665045
H	-0.814004436	7.161556692	21.323838322
C	-1.041182897	5.670013049	19.802337409
H	-2.073562998	5.976382914	19.578083704
H	-0.976490839	4.581443633	19.661387982
H	-0.377066824	6.138511734	19.066515716
C	-1.589881285	5.383315730	22.251291176
H	-2.642793762	5.620056683	22.036453547
H	-1.371705700	5.704093206	23.279406551
H	-1.470390927	4.289931611	22.204311119
C	3.919209900	7.641367555	22.848675062
H	3.286581264	8.395424998	23.338188616
C	4.623450811	8.346198804	21.676316874
H	5.239353823	9.180343543	22.044794699
H	3.886357005	8.749037541	20.964899816
H	5.276221294	7.644518277	21.137338892
C	4.936240676	7.149323753	23.885503975
H	5.485771353	8.004234604	24.305740679
H	5.677709910	6.469000984	23.440982889
H	4.440738354	6.619762347	24.712510343

[LCo$_2$ (N$_2$)].THF (6.THF)
FSPE= -4990.564622209300 Eh

Co	2.582448000	8.867321000	17.076321000
Co	1.083810000	9.041493000	21.243275000
N	1.962221000	10.103794000	18.477180000
N	1.082172000	9.967870000	19.544755000
N	2.598285000	10.325855000	15.958842000
N	2.495847000	7.659590000	15.659614000
N	-0.478891000	10.023410000	21.576480000
N	0.818111000	7.897060000	22.720385000
N	3.898838000	9.352310000	21.235979000
N	2.810624000	9.019250000	21.217937000
C	0.640823000	11.947387000	18.540365000
H	0.192780000	12.905468000	18.297852000
C	1.696734000	11.310179000	17.900202000
C	0.282319000	11.057108000	19.561350000
C	2.456274000	11.611826000	16.666039000
H	1.914935000	12.347170000	16.056004000
H	3.449963000	12.038041000	16.896614000
C	2.771891000	10.367094000	14.632616000
C	2.661790000	9.215162000	13.842829000
H	2.697435000	9.340237000	12.762134000
C	2.406140000	7.938573000	14.340527000
C	3.033563000	11.683476000	13.941781000
H	3.307386000	11.506776000	12.895560000
H	3.849480000	12.236965000	14.429241000
H	2.145669000	12.335122000	13.954507000
C	1.933801000	6.887390000	13.364087000
H	2.210043000	7.170901000	12.341378000
H	0.834869000	6.817092000	13.409232000
H	2.324326000	5.887846000	13.581335000
C	2.222062000	6.301560000	16.033448000
C	0.891610000	5.925828000	16.321320000
C	0.637867000	4.590897000	16.661292000
H	-0.382427000	4.280653000	16.890100000
C	1.671395000	3.658529000	16.725081000
H	1.458366000	2.624694000	17.002138000
C	2.981156000	4.048415000	16.439688000
H	3.781963000	3.310017000	16.494769000
C	3.284257000	5.368568000	16.085406000
C	-0.240862000	6.939249000	16.293313000
H	0.136682000	7.840317000	15.789180000
C	-0.636180000	7.349618000	17.721235000
H	-1.419067000	8.122152000	17.698519000
H	0.221239000	7.760804000	18.272276000
H	-1.020993000	6.486046000	18.283645000
C	-1.460786000	6.437258000	15.508718000
H	-2.211693000	7.236913000	15.429418000
H	-1.940370000	5.581207000	16.006530000
H	-1.182479000	6.125656000	14.491458000
C	4.711166000	5.777682000	15.748401000

H	4.788375000	6.852230000	15.972375000
C	5.766737000	5.030106000	16.573915000
H	6.754463000	5.484119000	16.409916000
H	5.838102000	3.974203000	16.273168000
H	5.550760000	5.057982000	17.648693000
C	5.034562000	5.601930000	14.252214000
H	6.095140000	5.832653000	14.072549000
H	4.436040000	6.264884000	13.617741000
H	4.852871000	4.563146000	13.936568000
C	-0.759215000	11.090554000	20.609688000
H	-0.770049000	12.073923000	21.108509000
H	-1.756816000	10.963950000	20.148146000
C	-1.282565000	9.958129000	22.646377000
C	-1.112497000	8.997021000	23.650019000
H	-1.817826000	9.013533000	24.478794000
C	-0.143578000	7.985907000	23.658543000
C	-2.408865000	10.950505000	22.808301000
H	-2.980340000	10.730201000	23.716770000
H	-3.098056000	10.925693000	21.950039000
H	-2.028844000	11.981954000	22.881359000
C	-0.263276000	6.941003000	24.748500000
H	-0.875465000	7.334009000	25.569292000
H	0.707522000	6.627358000	25.145553000
H	-0.753593000	6.033728000	24.365992000
C	1.637933000	6.722006000	22.800636000
C	1.182869000	5.537031000	22.165032000
C	1.879528000	4.348109000	22.416956000
H	1.541385000	3.419359000	21.957696000
C	3.018302000	4.331394000	23.223576000
H	3.544968000	3.392821000	23.405608000
C	3.498957000	5.520649000	23.767513000
H	4.406368000	5.505721000	24.373442000
C	2.820598000	6.732400000	23.574615000
C	0.017842000	5.559177000	21.178588000
H	0.121741000	6.509535000	20.623119000
C	0.079668000	4.400308000	20.177274000
H	-0.649237000	4.567443000	19.373046000
H	-0.174800000	3.442335000	20.656371000
H	1.069334000	4.295391000	19.715021000
C	-1.381971000	5.577295000	21.817544000
H	-2.143591000	5.524465000	21.024981000
H	-1.564163000	6.491369000	22.392699000
H	-1.522127000	4.707899000	22.478201000
C	3.342312000	8.003215000	24.232992000
H	2.852530000	8.850524000	23.734512000
C	4.862059000	8.152293000	24.065393000
H	5.181729000	9.147653000	24.406776000
H	5.164676000	8.037583000	23.016445000
H	5.406699000	7.406919000	24.664114000
C	2.980131000	8.079762000	25.727489000
H	3.434752000	8.975035000	26.177762000

H	3.358534000	7.197674000	26.266480000
H	1.896386000	8.139881000	25.883738000
O	3.325995000	7.489471000	18.299649000
C	4.716231000	7.729416000	18.693452000
H	4.804522000	8.767078000	19.039361000
H	5.326134000	7.590931000	17.792071000
C	5.024759000	6.713585000	19.796688000
H	5.077821000	7.219406000	20.767654000
H	5.984289000	6.211426000	19.619209000
C	3.843589000	5.726362000	19.770017000
H	3.609154000	5.341856000	20.768734000
H	4.052608000	4.872617000	19.111758000
C	2.691945000	6.539306000	19.205679000
H	1.991731000	5.954727000	18.602873000
H	2.144566000	7.100452000	19.977677000

[$LCo_2(N_2)_2$] ($6.N_2$)
FSPE= -4867.517858303883 Eh

Co	1.665013000	8.414181000	16.814396000
Co	1.439950000	9.032524000	20.945008000
N	1.669031000	9.857851000	18.047004000
N	1.193419000	10.009195000	19.335660000
N	2.291646000	9.710078000	15.609296000
N	1.851380000	7.004614000	15.574493000
N	0.520034000	10.474360000	21.718674000
N	1.487029000	8.050986000	22.556050000
C	1.214074000	12.046432000	18.363271000
H	1.103951000	13.112951000	18.197408000
C	1.695937000	11.083069000	17.471207000
C	0.905364000	11.319462000	19.517558000
C	2.174903000	11.104233000	16.071995000
H	1.477915000	11.661334000	15.423557000
H	3.146030000	11.625882000	16.013817000
C	2.814494000	9.524076000	14.393464000
C	2.890698000	8.257658000	13.799934000
H	3.326621000	8.202528000	12.804664000
C	2.384220000	7.073596000	14.340127000
C	3.314058000	10.702786000	13.596165000
H	3.699602000	10.367882000	12.627294000
H	4.118842000	11.234814000	14.126585000
H	2.511165000	11.434277000	13.416230000
C	2.400353000	5.851196000	13.451170000
H	3.111081000	5.996204000	12.629032000
H	1.404742000	5.685321000	13.012963000
H	2.662374000	4.938337000	13.998472000
C	1.302299000	5.729173000	15.953159000
C	-0.002157000	5.388420000	15.536872000
C	-0.529837000	4.159405000	15.955510000
H	-1.542957000	3.881682000	15.659479000
C	0.219299000	3.285552000	16.740508000

H	-0.205854000	2.330395000	17.053825000
C	1.510533000	3.637325000	17.134937000
H	2.082542000	2.951405000	17.759612000
C	2.073363000	4.864490000	16.764004000
C	-0.843507000	6.325017000	14.680955000
H	-0.175440000	7.094420000	14.269375000
C	-1.904319000	7.053800000	15.524289000
H	-2.522071000	7.699124000	14.881938000
H	-1.437921000	7.683346000	16.292166000
H	-2.567855000	6.332933000	16.025654000
C	-1.503740000	5.596200000	13.500811000
H	-2.002902000	6.321364000	12.841719000
H	-2.265443000	4.880576000	13.843884000
H	-0.765548000	5.040832000	12.904408000
C	3.466046000	5.263116000	17.230268000
H	3.436755000	6.353983000	17.391391000
C	3.869004000	4.606325000	18.553467000
H	4.818756000	5.032323000	18.904930000
H	4.014314000	3.520754000	18.443549000
H	3.120888000	4.775186000	19.338733000
C	4.541365000	4.986701000	16.163513000
H	5.534157000	5.250783000	16.557420000
H	4.378092000	5.571656000	15.250674000
H	4.554349000	3.919652000	15.892466000
C	0.385992000	11.663486000	20.859045000
H	0.945037000	12.509406000	21.293584000
H	-0.667163000	11.985792000	20.784651000
C	-0.014770000	10.554620000	22.940768000
C	0.130685000	9.531787000	23.886539000
H	-0.334109000	9.684772000	24.858328000
C	0.891562000	8.372398000	23.719776000
C	-0.776407000	11.789846000	23.352873000
H	-1.128627000	11.690748000	24.385125000
H	-1.647800000	11.956698000	22.700993000
H	-0.147114000	12.690296000	23.283953000
C	1.079994000	7.494670000	24.936083000
H	0.299694000	7.708648000	25.676029000
H	2.055083000	7.696364000	25.405010000
H	1.061640000	6.427285000	24.688629000
C	2.318429000	6.876049000	22.577319000
C	1.801891000	5.658995000	22.077488000
C	2.639971000	4.537241000	22.085339000
H	2.266126000	3.586581000	21.705285000
C	3.952541000	4.618892000	22.551560000
H	4.593017000	3.735299000	22.535641000
C	4.446336000	5.831947000	23.027244000
H	5.476978000	5.892863000	23.380863000
C	3.640136000	6.977756000	23.059888000
C	0.383808000	5.577459000	21.532467000
H	0.178667000	6.552324000	21.059637000
C	0.212256000	4.493943000	20.464757000

H	-0.782886000	4.577643000	20.007010000
H	0.295737000	3.482631000	20.891472000
H	0.954459000	4.589131000	19.662135000
C	-0.661625000	5.379242000	22.645499000
H	-1.664541000	5.287956000	22.202255000
H	-0.683613000	6.219671000	23.349343000
H	-0.454924000	4.458803000	23.213447000
C	4.208103000	8.292607000	23.574383000
H	3.365396000	8.975343000	23.750690000
C	5.113137000	8.955976000	22.522194000
H	5.546739000	9.884288000	22.923546000
H	4.548727000	9.208159000	21.615266000
H	5.938840000	8.286065000	22.238418000
C	4.960667000	8.122446000	24.902912000
H	5.253006000	9.105862000	25.298834000
H	5.878415000	7.530284000	24.773111000
H	4.338629000	7.619180000	25.657203000
N	2.652411000	8.029442000	20.166712000
N	3.524068000	7.550885000	19.638534000
N	0.663025000	7.471040000	17.904063000
N	-0.107536000	7.015323000	18.586989000

2.[LCo$_2$ (N$_2$)]$^-$. K•THF (optimized crystal structure of 2 with a K.THF)
FSPE= -10352.342089014610 Eh

Co	2.358253000	9.092359000	17.130840000
Co	1.313932000	9.471703000	20.978682000
N	1.880924000	10.567988000	18.229077000
N	1.426779000	10.679686000	19.517238000
N	2.499863000	10.414881000	15.820214000
N	2.599498000	7.650446000	15.969497000
N	0.712717000	10.992395000	21.884173000
N	1.222104000	8.325555000	22.444734000
N	2.173308000	8.209107000	18.651881000
N	1.883466000	8.319015000	19.764291000
C	1.362873000	12.735146000	18.587339000
H	1.216840000	13.799808000	18.447421000
C	1.847657000	11.797684000	17.660892000
C	1.104428000	11.975675000	19.738254000
C	2.355479000	11.811746000	16.266887000
H	1.681390000	12.379776000	15.606774000
H	3.345436000	12.311770000	16.224416000
C	2.758633000	10.222096000	14.518225000
C	2.916335000	8.942644000	13.964855000
H	3.139063000	8.894231000	12.900168000
C	2.805401000	7.719937000	14.642647000
C	2.861549000	11.407185000	13.590354000
H	3.129339000	11.075668000	12.580974000
H	3.633332000	12.114369000	13.926292000
H	1.909521000	11.959521000	13.533072000
C	2.881998000	6.455150000	13.816448000
H	3.380633000	6.663258000	12.861296000

H	1.876904000	6.063076000	13.592801000
H	3.426325000	5.656086000	14.335736000
C	2.251521000	6.374751000	16.520731000
C	0.940797000	5.879304000	16.326701000
C	0.593285000	4.659407000	16.922029000
H	-0.417882000	4.269451000	16.795443000
C	1.512896000	3.944775000	17.688395000
H	1.223181000	3.001034000	18.154059000
C	2.800144000	4.449935000	17.875107000
H	3.505297000	3.893052000	18.492171000
C	3.192760000	5.666250000	17.303158000
C	-0.092117000	6.681008000	15.546570000
H	0.452337000	7.340915000	14.856884000
C	-0.890850000	7.596274000	16.489756000
H	-1.588788000	8.226110000	15.916712000
H	-0.206098000	8.255982000	17.047025000
H	-1.472360000	7.003184000	17.210727000
C	-1.026993000	5.805013000	14.703974000
H	-1.667727000	6.438808000	14.073329000
H	-1.689772000	5.189956000	15.330860000
H	-0.459348000	5.129692000	14.046370000
C	4.586387000	6.230384000	17.528490000
H	4.459807000	7.323268000	17.611798000
C	5.237646000	5.728356000	18.818858000
H	6.166443000	6.284906000	19.010197000
H	5.503482000	4.661542000	18.756274000
H	4.575338000	5.861930000	19.684623000
C	5.511260000	5.965805000	16.328765000
H	6.518368000	6.360902000	16.530592000
H	5.136933000	6.449927000	15.418788000
H	5.599842000	4.885240000	16.136031000
C	0.612576000	12.244933000	21.110073000
H	1.220099000	13.033038000	21.581456000
H	-0.432605000	12.603173000	21.088390000
C	0.398443000	11.063934000	23.185932000
C	0.455289000	9.952096000	24.041552000
H	0.174413000	10.118855000	25.080475000
C	0.845673000	8.649954000	23.692832000
C	-0.024943000	12.384649000	23.783365000
H	-0.253229000	12.265581000	24.848699000
H	-0.915843000	12.793168000	23.280507000
H	0.770030000	13.140998000	23.683556000
C	0.847009000	7.595606000	24.775691000
H	0.453137000	8.011613000	25.710749000
H	1.863059000	7.216881000	24.965925000
H	0.236704000	6.724972000	24.493581000
C	1.621490000	6.967850000	22.208379000
C	0.681234000	6.038218000	21.716154000
C	1.113468000	4.739145000	21.419018000
H	0.400078000	4.017296000	21.016514000
C	2.439462000	4.357940000	21.619588000

H	2.762640000	3.344695000	21.374418000
C	3.353824000	5.281409000	22.127629000
H	4.392425000	4.981565000	22.280643000
C	2.963853000	6.591953000	22.431005000
C	-0.766980000	6.430009000	21.484918000
H	-0.885139000	7.458477000	21.855364000
C	-1.095979000	6.446006000	19.985323000
H	-2.136955000	6.763572000	19.819831000
H	-0.967998000	5.449468000	19.535586000
H	-0.434390000	7.143217000	19.455907000
C	-1.737163000	5.525022000	22.259056000
H	-2.772341000	5.875362000	22.129780000
H	-1.508128000	5.521680000	23.334863000
H	-1.687823000	4.485788000	21.899625000
C	3.980781000	7.606335000	22.920548000
H	3.418963000	8.466275000	23.310780000
C	4.824679000	8.104287000	21.740728000
H	5.520549000	8.887405000	22.067526000
H	4.182371000	8.518351000	20.950818000
H	5.413508000	7.283739000	21.307590000
C	4.874259000	7.075908000	24.048286000
H	5.530011000	7.878767000	24.417237000
H	5.519609000	6.254018000	23.703106000
H	4.276227000	6.705055000	24.893678000
Co	6.363553000	12.503260000	16.625637000
Co	7.232634000	13.344410000	20.449957000
K	4.183519000	11.661022000	19.566588000
O	3.588041000	12.482198000	22.023627000
N	6.666963000	11.443080000	18.179879000
N	7.054406000	11.731064000	19.462508000
N	6.262787000	10.826128000	15.803547000
N	6.231819000	13.495509000	15.046399000
N	7.487611000	12.168053000	21.878878000
N	7.435352000	14.898421000	21.461050000
N	6.620685000	13.825472000	17.771087000
N	6.858470000	14.071993000	18.876161000
C	6.934093000	9.482448000	19.270358000
H	6.963564000	8.422813000	19.497408000
C	6.594415000	10.095449000	18.054457000
C	7.216630000	10.559850000	20.125520000
C	6.186353000	9.654340000	16.698775000
H	6.824569000	8.829807000	16.349096000
H	5.140520000	9.275356000	16.714292000
C	6.271470000	10.589649000	14.483151000
C	6.264164000	11.622873000	13.534026000
H	6.255804000	11.323482000	12.487235000
C	6.227392000	12.999733000	13.796696000
C	6.309450000	9.169813000	13.970884000
H	6.284301000	9.163836000	12.875012000
H	5.446430000	8.593599000	14.333726000
H	7.223066000	8.648439000	14.299159000

C	6.172666000	13.940890000	12.615877000
H	6.270802000	13.380876000	11.678356000
H	6.968695000	14.697270000	12.659533000
H	5.218759000	14.489963000	12.590963000
C	6.316331000	14.920970000	15.195967000
C	5.145710000	15.695913000	15.327251000
C	5.269629000	17.069635000	15.579386000
H	4.367948000	17.674047000	15.701012000
C	6.521735000	17.672569000	15.684491000
H	6.602296000	18.739965000	15.896882000
C	7.671851000	16.900756000	15.519513000
H	8.653209000	17.372822000	15.599460000
C	7.592150000	15.526476000	15.266053000
C	3.768834000	15.070163000	15.216122000
H	3.917661000	14.013790000	14.952957000
C	3.036804000	15.115426000	16.561469000
H	2.032319000	14.678892000	16.471114000
H	2.926922000	16.150267000	16.917423000
H	3.593356000	14.553285000	17.323923000
C	2.921220000	15.721471000	14.113072000
H	1.963217000	15.190708000	14.007177000
H	3.435966000	15.695248000	13.141848000
H	2.699382000	16.773304000	14.349122000
C	8.865895000	14.716859000	15.089728000
H	8.567508000	13.679869000	14.882354000
C	9.694365000	14.710083000	16.380275000
H	10.609499000	14.112311000	16.251193000
H	9.115246000	14.283920000	17.209948000
H	9.990814000	15.732132000	16.659323000
C	9.704472000	15.225083000	13.905991000
H	10.586309000	14.583113000	13.760784000
H	10.060925000	16.251340000	14.083595000
H	9.124028000	15.225804000	12.972165000
C	7.629157000	10.738099000	21.540527000
H	8.677683000	10.418511000	21.688072000
H	7.020956000	10.106759000	22.206824000
C	7.574233000	12.504547000	23.175975000
C	7.525552000	13.836659000	23.613890000
H	7.554005000	14.000174000	24.690086000
C	7.490279000	14.980051000	22.799664000
C	7.733182000	11.428618000	24.224522000
H	7.806801000	11.878500000	25.221086000
H	8.637278000	10.824630000	24.048720000
H	6.880410000	10.730173000	24.224715000
C	7.484776000	16.330768000	23.477754000
H	7.799426000	16.236177000	24.524276000
H	6.472069000	16.764523000	23.466093000
H	8.142668000	17.049434000	22.971583000
C	7.676331000	16.081682000	20.687718000
C	6.612778000	16.941000000	20.342462000
C	6.878193000	18.025741000	19.495396000

H	6.061589000	18.687453000	19.200074000
C	8.164653000	18.268924000	19.018656000
H	8.351570000	19.112346000	18.351789000
C	9.213242000	17.423842000	19.388033000
H	10.216919000	17.617874000	19.007521000
C	8.993241000	16.320844000	20.221452000
C	5.202754000	16.693624000	20.851693000
H	5.276879000	15.938199000	21.644331000
C	4.572744000	17.955541000	21.458791000
H	3.596865000	17.713869000	21.906183000
H	4.405450000	18.730364000	20.695457000
H	5.212961000	18.385869000	22.242557000
C	4.306281000	16.111239000	19.750547000
H	3.291655000	15.921710000	20.133427000
H	4.720121000	15.166389000	19.377980000
H	4.226319000	16.804370000	18.899896000
C	10.129257000	15.389953000	20.620893000
H	9.677741000	14.384170000	20.698962000
C	10.704016000	15.754105000	22.001859000
H	11.523242000	15.067278000	22.263910000
H	9.946584000	15.690867000	22.791711000
H	11.107697000	16.778992000	21.992336000
C	11.266091000	15.320655000	19.597779000
H	11.984408000	14.543648000	19.897828000
H	11.819128000	16.270621000	19.533792000
H	10.894582000	15.075236000	18.596123000
C	4.075434000	13.686990000	22.649979000
H	3.476563000	14.518909000	22.257268000
H	5.131594000	13.834299000	22.368569000
C	3.923100000	13.462854000	24.164932000
H	3.035098000	13.976350000	24.556847000
H	4.803582000	13.844712000	24.696960000
C	3.776530000	11.923687000	24.308038000
H	2.768553000	11.654386000	24.649507000
H	4.502918000	11.495132000	25.010766000
C	3.999798000	11.404678000	22.885898000
H	5.069833000	11.191112000	22.711966000
H	3.389453000	10.532099000	22.613334000

VI (Dicobalt(I) complex of the pyrazolate ligand system with two tridentate PNN compartments)
FSPE= -5133.428307641489 Eh

Co	-2.076383000	-0.213774000	0.160386000
Co	2.088314000	-0.219770000	-0.186147000
P	-3.946908000	0.900256000	0.065330000
P	3.959949000	0.893356000	-0.101969000
N	-0.668034000	-1.490787000	-0.064994000
N	-3.157995000	-1.726837000	-0.257209000
N	-1.177409000	1.018323000	0.934334000
N	-0.611766000	1.766052000	1.579063000
N	0.677742000	-1.490997000	0.056250000
N	3.166690000	-1.727514000	0.258201000

N	1.192384000	1.001557000	-0.980463000
N	0.628371000	1.740135000	-1.637049000
C	-5.114311000	-0.377053000	-0.283114000
C	-3.904154000	2.080223000	-1.423718000
C	-4.579432000	1.777431000	1.625515000
C	-1.095151000	-2.793815000	-0.118165000
C	-2.525188000	-2.939603000	-0.291384000
C	-4.542987000	-1.638163000	-0.411915000
C	-3.614399000	1.166377000	-2.628112000
C	-5.240416000	2.800007000	-1.639923000
C	-2.757152000	3.093139000	-1.306802000
C	-6.106536000	1.947353000	1.596848000
C	-3.915405000	3.139860000	1.864788000
C	-4.216409000	0.825137000	2.779886000
C	1.102684000	-2.793691000	0.130435000
C	0.003031000	-3.654756000	0.013011000
C	-3.203618000	-4.134332000	-0.496946000
C	-5.258685000	-2.857012000	-0.674900000
C	2.532152000	-2.938816000	0.308910000
C	-4.601994000	-4.067708000	-0.704395000
C	5.124468000	-0.379616000	0.271283000
C	4.600119000	1.743578000	-1.673924000
C	3.912871000	2.097182000	1.367802000
C	4.551233000	-1.638072000	0.416571000
C	3.208437000	-4.131118000	0.534658000
C	3.935741000	3.100775000	-1.940319000
C	6.126824000	1.916093000	-1.640530000
C	4.243900000	0.769917000	-2.812479000
C	2.771222000	3.113480000	1.227917000
C	3.612121000	1.203849000	2.584730000
C	5.250828000	2.814874000	1.580248000
C	5.264519000	-2.853718000	0.700146000
C	4.606232000	-4.063048000	0.745484000
H	-6.185446000	-0.229577000	-0.406310000
H	6.195362000	-0.231523000	0.395768000
H	-3.504286000	1.785518000	-3.533292000
H	-4.425706000	0.443524000	-2.789055000
H	-2.680657000	0.603364000	-2.472338000
H	-5.217723000	3.324575000	-2.609883000
H	-5.433557000	3.551156000	-0.862352000
H	-6.082575000	2.093124000	-1.660322000
H	-2.710219000	3.689218000	-2.233455000
H	-1.792679000	2.583389000	-1.183818000
H	-2.889666000	3.788883000	-0.470562000
H	-6.431008000	2.394566000	2.551001000
H	-6.612343000	0.978781000	1.487405000
H	-6.439966000	2.607563000	0.786688000
H	-4.204926000	3.504777000	2.864210000
H	-4.243658000	3.890468000	1.133693000
H	-2.820364000	3.077759000	1.835283000
H	-4.646434000	1.212355000	3.718387000

H	-3.128936000	0.737444000	2.902208000
H	-4.622231000	-0.181400000	2.602344000
H	0.002046000	-4.739626000	0.022058000
H	-2.662266000	-5.078651000	-0.511553000
H	-6.336197000	-2.799029000	-0.833525000
H	-5.164400000	-4.985258000	-0.890987000
H	2.665838000	-5.074437000	0.562090000
H	4.228335000	3.448633000	-2.944952000
H	4.260602000	3.864422000	-1.221267000
H	2.840661000	3.038148000	-1.913543000
H	6.455518000	2.347302000	-2.600592000
H	6.633815000	0.950507000	-1.511800000
H	6.454907000	2.590918000	-0.840338000
H	4.677975000	1.140075000	-3.755981000
H	3.157087000	0.678491000	-2.938128000
H	4.650438000	-0.232416000	-2.614033000
H	2.720505000	3.723048000	2.145533000
H	1.805179000	2.606728000	1.105133000
H	2.914129000	3.796275000	0.382870000
H	3.502934000	1.837294000	3.480068000
H	4.417563000	0.477657000	2.759644000
H	2.675082000	0.645073000	2.433771000
H	5.224259000	3.355567000	2.541201000
H	5.451519000	3.552308000	0.791543000
H	6.090084000	2.105219000	1.617428000
H	6.341569000	-2.794695000	0.861511000
H	5.166890000	-4.978376000	0.947623000

N_2

FSPE= -109.677717673803 Eh

N	0.000000000	0.000000000	-0.001107000
N	0.000000000	0.000000000	1.101107000
